# Network Pharmacology Reveals the Mechanism of Activity of Tongqiao Huoxue Decoction Extract Against Middle Cerebral Artery Occlusion-Induced Cerebral Ischemia-Reperfusion Injury

**DOI:** 10.3389/fphar.2020.572624

**Published:** 2021-01-11

**Authors:** Si-peng Wu, Ning Wang, Li Zhao

**Affiliations:** ^1^ Key Laboratory of Chinese Medicinal Formula of Anhui Province, Anhui University of Chinese Medicine, Hefei, China; ^2^ State Key Laboratory of Cellular Stress Biology, School of Life Sciences, Xiamen University, Xiamen, China; ^3^ Institute for Pharmacodynamics and Safety Evaluation of Chinese Medicine, Anhui Academy of Chinese Medicine, Hefei, China; ^4^ Key Laboratory of Xin’an Medicine, Ministry of Education, Hefei, China

**Keywords:** Tongqiao Huoxue Decoction, cerebral ischemia reperfusion injury, angiogenesis, vascular endothelial growth factor, network pharmacology

## Abstract

Several clinical therapies such as tissue repair by replacing injured tissues with functional ones have been reported; however, there is great potential for exploring traditional herbal-induced regeneration with good safety. Tongqiao Huoxue Decoction (TQHXD), a well-known classical traditional Chinese medicinal formula, has been widely used for clinical treatment of stroke. However, biological activity and mechanisms of action of its constituents toward conferring protection against cerebral ischemia-reperfusion (I/R) injury remain unclear. In this present study, we evaluated TQHXD quality using HPLC; then, it was screened for its potential active ingredients using a series of indices, such as their drug-likeness and oral bioavailability. Subsequently, we analyzed the potential mechanisms of TQHXD anti-I/R using gene ontology functional enrichment analyses. The network pharmacological approach enabled us to screen 265 common targets associated with I/R, indicating that TQHXD had remarkable protective effects on infarction volume, neurological function scores, and blood-brain barrier (BBB) injury. In addition, TQHXD significantly promoted the recovery of regional cerebral blood flow (rCBF) 7 days after reperfusion compared to rats in the vehicle group. Immunofluorescence results revealed a significantly higher CD34 expression in TQHXD-treated rats 7 days after reperfusion. TQHXD is not merely effective but eventually develops a secretory profile composed of VEGF and cerebral blood flow, a typical signature termed the angiogenesis-associated phenotype. Mechanistically, our data revealed that TQHXD (6 g/kg) treatment resulted in a marked increase in expression of p-focal adhesion kinase (FAK) and p-Paxillin proteins. However, Ki8751-mediated inhibition of VEGFR2 activity repealed its angiogenesis and protective effects and decreased both p-FAK and p-Paxillin protein levels. Taken together, these findings affirmed the potential of TQHXD as a drug for the management of stroke, which might be exerted by increasing the angiogenesis via the VEGF pathway. Therefore, these results provide proof-of-concept evidence that angiogenesis is a major contributor to TQHXD-treated I/R and that TQHXD is a promising traditional ethnic medicine for the management of this condition.

## Introduction

Stroke is the second leading cause of death in the world owing to its high morbidity, mortality, and disability rates (Benjamin et al., 2018). Approximately four-fifths of all patients with stroke suffer from ischemia stroke, and most of them live with disabilities for a long time (Mozaffarian et al., 2015). One of the principles of clinical treatment of ischemic stroke is to restore blood flow in time. Although this approach is an effective protection measure, long-term accumulation of reperfusion can lead to cerebral ischemia-reperfusion (I/R) injury. Treatment of I/R injury-induced ischemic stroke is challenging. To date, recombinant plasminogen activator is still the only drug approved by the Food and Drug Administration (FDA) for treating stroke. Therefore, the development of novel drugs is necessary to aid in the management of patients with stroke.

Angiogenesis, a basic biological process that aids in the development of new blood vessels from existing capillaries, has been implicated in multiple pathological and physiological processes (Beck and Plate, 2009). Although the connection between the brain and blood is a new focus in scientific researches, theories have linked this connection to traditional Chinese medicine. For example, many studies have shown that angiogenesis plays a pivotal role in many pathological conditions, such as cerebrovascular and cardiovascular diseases and cancer (Ferrara and Kerbel, 2005). These diseases involve actions of multiple growth factors, receptors, and molecules, resulting in diverse signaling pathways that affect the pathogenicity of angiogenesis across different diseases (Carmeliet, 2000). In addition, angiogenesis mediates the production of new blood vessels, thereby contributing to patient recovery after ischemic injury (Liu et al., 2017). Although angiogenesis is elevated during ischemia and hypoxia, this process is too slow to meet the needs of body recovery (Krupinski et al., 1994). Therefore, accelerating angiogenesis during the establishment of collateral circulation has been proposed as an attractive approach for treating cerebral ischemia injury (Liu et al., 2014).

Traditional Chinese medicine (TCM), which has been used for more than 2000 years with the characteristics of multiple pathways and multiple targets, is likely to represent novel therapeutic targets for developing effective treatment against I/R (Wu et al., 2019; Peng et al., 2019). In fact, numerous TCM-based remedies for stroke have been introduced into the market, owing to their unique advantages (Wu et al., 2019; Chen et al., 2009; She et al., 2019). For example, Tongqiao Huoxue Decoction (TQHXD), a classic traditional Chinese herbal formula, has been used to treat stroke. It had subsequently been embodied in Corrections on the Errors of Medical Works prescribed by Qingren Wang, comprising eight Chinese herbs (Table 1). Currently, TQHXD is widely used to treat patients with ischemic stroke (Yang and Fan, 2009; Liu et al., 2003). The key active components for this drug include paeoniflorin, ferulic acid, and muscone (Xu et al., 2008; Wu et al., 2018). Collectively, TQHXD controls stroke via various mechanisms. For instance, the drug can module permeability of the blood-brain barrier (BBB) and activity of plasminogen activator inhibitor-1 (Li et al., 2017; Wang et al., 2012; Kim et al., 2016; Wu et al., 2019). However, the mechanisms involved in TQHXD have only partially been unraveled. There is still a thought-provoking question of whether TQHXD can protect against the I/R injury by targeting angiogenesis.

**TABLE 1:** The composition of TQHXD.

	Chinese medicine	Family	Weight (g)
1	Taoren (Semen Persicae)	Rosaceae	9
2	Chishao (Radix Paeoniae Rubra)	Ranunculaceae	3
3	Chuanxiong (Rhizoma Ligustici Chuanxiong)	Apiaceae	3
4	Honghua (*Flos carthami*)	Asteraceae	9
5	Dazao (Fructus Jujubae)	Rhamnaceae	5
6	Cong (Scallion)	Liliaceae	3
7	Shexiang (*Moschus*)	Cervidae	0.15
8	Shengjiang (*Zingiber officinale* Roscoe)	Zingiberaceae	9

The present study sought to provide new insights into TQHXD’s anti-I/R effects in rats. Summarily, we employed network pharmacology, an approach by using high-throughput data from public databases to identify interactions among “compound-target-disease,” and gave a holistic cognition of the relationship between formula and diseases (Hopkins, 2008). This approach’s systemic and holistic traits are in line with the concept of TCM (Pan et al., 2020; Mou et al., 2020). In this work, the network pharmacology approach was used to provide a new understanding of TQHXD that exert anti-I/R effects, and TQHXD was intragastrically administered to rats with MCAO for 7 days to explore the mechanism.

## Methods and Materials

### Animals

Male healthy Sprague Dawley rats weighing 240–260 g were purchased from the Laboratory Animal Center of Anhui Medical University (Hefei, certificate of quality: No. SXCK 2011-002, China). All rats had free access to standard diet and water. All experimental rats were approved by the Institutional Animal Care and Use Committee at Anhui University of Chinese Medicine. In addition, rats had undergone treatments according to “3 Rs” rules (Reduction, Refinement, and Replacement) in all experiments.

### Herbs and Reagents

Herbs of TQHXD were purchased from Xin He Traditional Chinese Medicines Group Corporation (Hefei, China). Furthermore, each herb was authenticated by the herbal medicinal botanist, Professor Shoujin Liu, at the Anhui University of Chinese Medicine. Nimodipine Tablets (NMDP) were purchased from Bayer (Batch Number: H20003010), and 2% triphenyltetrazolium chloride (TTC) dye solution was purchased from Sigma Chemical. Ki8751 (VEGFR2 special inhibitor) was selected from Selleck Chemicals Co., Ltd. Rabbit primary antibodies for VEGF-A, p-FAK (Tyr576), p-Paxillin (Tyr118), and β-actin were bought from CST (Boston, United States). The mouse antibodies against FAK and Paxillin were bought from Abcam (Cambridge, MA, United States). The secondary horseradish peroxidase- (HRP-) labeled antibodies were acquired from Santa Cruz Biotechnology (Santa Cruz, CA, United States). All of the reagents or herbs were standard and commercially available.

### Network Pharmacology

In this section, the intersect target enrichment analysis and protein-protein interaction (PPI) network were utilized to reveal the multitarget, multifunction, and multipathway therapeutic advantages of TQHXD in protecting I/R.

#### Collection of Potential Active Ingredients and Disease Targets in TQHXD

Three TCM databases were used to collect the active ingredients of TQHXD, including the traditional Chinese medicine systems pharmacology database (TCMSP) (Ru et al., 2014), (TCMSP™, http://lsp.nwsuaf.edu.cn/tcmsp.php), the Traditional Chinese Medicine Integrated Database (Xue et al., 2013) (TCMID, http://www.megabionet.org/tcmid/), and the TCM Database @Taiwan (Chen et al., 2014) (http://tcm.cmu.edu.tw/). Importantly, the interrelated scientific literature databases (PubMed, MEDLINE, and China National Knowledge Infrastructure) were also employed in this work. Subsequently, we set an OB (oral bioavailability) value of ≥30% and DL (drug-like quality) value of ≥0.18 as standard to screen out the potential ingredients of TQHXD. Secondly, the 2D structure of components was transformed into SDF structure format through the PubChem database (https://Pubchem.ncbi.nlm.nih.gov/). Subsequently, the targets were obtained using SwissTargetPrediction (http://new.swisstargetprediction.ch/index.php) and TCMSP, an online tool for predicting targets. In addition, related targets belonging to *Homo sapiens* were renamed retrieved from the UniProt database (https://www.uniprot.org/uniprot/). Finally, the NIMNT database (http://www.idrug.net.cn/NIMNT/) was used to enrich the possible indications of TQHXD.

### Disease Target

The keyword “cerebral ischemia reperfusion injury” was used in GeneCards (https://www.genecards.org/), OMIM (Online Mendelian Inheritance in Man, https://omim.org/) and DrugBank databases (https://www.drugbank.ca/) to search for I/R-related targets. These databases (Wishart et al., 2018) are free bioinformatics resources that combine disease data with comprehensive targets. Finally, these targets were intersected with related targets of TQHXD by the online tool (http://bioinfogp.cnb.csic.es/tools/venny/index.html).

### Gene Ontology Functional Enrichment Analysis

The Database for Annotation, Visualization, and Integrated Discovery (DAVID; http://david.abcc.ncifcrf.gov/home.jsp, version 6.8), together with pathway data obtained from the KEGG database, was used to find the identification of characteristic biological attributes of the potential target for I/R in TQHXD. Finally, FunRich tool (http://www.funrich.org/) was used to analyze more biological characteristics of a common target.

#### Protein-Protein Interaction Network

Generally speaking, it is difficult for a single protein to complete a series of complex biological processes. Hence, it is important to find the PPI network. The overlapped components-targets belonging to *Homo sapiens* were imported to the STRING database (https://string-db.org/, version 11.0), and an organism entry was set with the minimum required interaction score = 0.900. Finally, the PPI network was visualized in Cytoscape (https://cytoscape.org/, version 3.7.2). A higher degree value node represented putative crucial targets of herbs in the PPI network.

### Experimental Verification of the Protective Effect of Tongqiao Huoxue Decoction Against Ischemia-Reperfusion

#### Establishment of Ischemia-Reperfusion Model

The cerebral I/R injury model *in vivo* was performed according to Longa’s method (E. Z. Longa et al., 1989). Firstly, rats were anesthetized with 4% isoflurane (4% for anesthesia induction; 2% for anesthesia maintenance, 3 ml/kg). Then, the internal carotid artery (ICA), external carotid artery (ECA), and left common carotid artery (CCA) were carefully separated by tweezers. Subsequently, the left CCA and ECA were blocked by microvascular aneurysm clips, followed by occluding the middle cerebral artery (MCA) through inserting a filament (Doccol, United States) coated through the ECA (18 ± 2 mm). Two hours later, the filament was removed to achieve reperfusion. Sham group received the same procedures, but no filament was inserted into the MCA. The temperature of the operation chamber was maintained at 37 ± 0.5 °C throughout the whole surgery. After waking up, the rats were put back into the cages and had free access to water and food.

### Preparation of Tongqiao Huoxue Decoction Sample

The ethanol extract of TQHXD was prepared as previously described in (Chao-Liang et al., 2015). *Prunus persica* (L.) Batsch 90 g (Xin He Traditional Chinese Medicines Co., Ltd., Hefei, China, product batch number: 1702181), *Paeonia anomala* subsp. *veitchii* (Lynch) D.Y.Hong and K.Y.Pan [Paeoniaceae] 30 g (Xin He Traditional Chinese Medicines Co., Ltd., Hefei, China, product batch number: 1709201), *Conioselinum anthriscoides* “Chuanxiong” [Apiaceae] 30 g (Xin He Traditional Chinese Medicines Co., Ltd., Hefei, China, product batch number: 1701033), *Carthamus tinctorius* L. 90 g (Xin He Traditional Chinese Medicines Co., Ltd., Hefei, China product batch number:1707213) 90 g, *Ziziphus jujuba* Mill. 50 g (Huidu Traditional Chinese Medicines Co., Ltd., Bozhou, China, product batch number: 1707223), *Zingiber officinale* Roscoe 90 g (Zhejiang Chinese Medical University prepared pieces Co., Ltd., Hangzhou, Zhejiang, China, product batch number: 190601), and fresh *Allium fistulosum* L [Amaryllidaceae] 30 g (WalMart Inc. Hefei, China) were extracted in 3200 ml 75% ethanol for 2 h and filtered. After the confluent solutions pump vacuum, 1,600 ml 75% ethanol was added to it and then pump-vacuumed again until there is no alcoholic taste. Finally, 1.5 g *Moschus* (Lasa Chinese Medicines Co., Ltd., Lasa, China, product batch number: 62523635662) was added to the solutions to form the extract solutions with a concentration of 2 g/ml (equivalent to the dry weight of raw materials). The medicinal plant scientific names have been unified using the Kew Science database (https://mpns.science.kew.org/mpns-portal/?_ga=1.111763972.1427522246.1459077346%20or%20http://www.plantsoftheworldonline.org/%20or%20www.theplantlist.org).

#### High-Performance Liquid Chromatography (HPLC)

The quality control reproducible experiments of TQHXD were conducted using HPLC. The quality control of TQHXD was completed by paeoniflorin (a characteristic pharmaceutically active component of TQHXD) in our work. Firstly, TQHXD solutions were put into the HPLC facilities (Agilent 1100 HPLC system, United States). The separations were carried out on a Phenomenex C18 column (4.6 mm × 250 mm, 5 μm) with the following conditions: temperature, 30 °C; flow rate, 1.0 ml min^−1^. The mobile phase consisted of methanol (A)-H_2_O (0.05% V/V phosphoric acid), and gradient elution was set as follows: 0–15 min, 10% A−28% A; 15–30 min, 28% A−40% A; 30–55 min, 40% A−48% A; 55–70 min, 48% A−54% A. Finally, the detection wavelength and the injection volume were 254 nm and 20 μL, respectively.

#### Experimental Groups and Drug Administration

Firstly, to investigate whether the protective effects of TQHXD were involved in angiogenesis (experiment 1), rats were randomly divided into six groups: the sham group, vehicle group, TQHXD group (3 g/kg, 6 g/kg, and 12 g/kg), and positive group (NMDP, 0.02 g/kg). Dosages of TQHXD (3, 6, and 12 g/kg) and NMDP (0.02 g/kg) groups were equivalent to the dose of clinical adult patients. Rats in the TQHXD and NMDP groups were treated with respective doses by intragastric administration once a day for 7 days after reperfusion. Rats in sham or vehicle groups were given normal saline.

**FIGURE 1:**
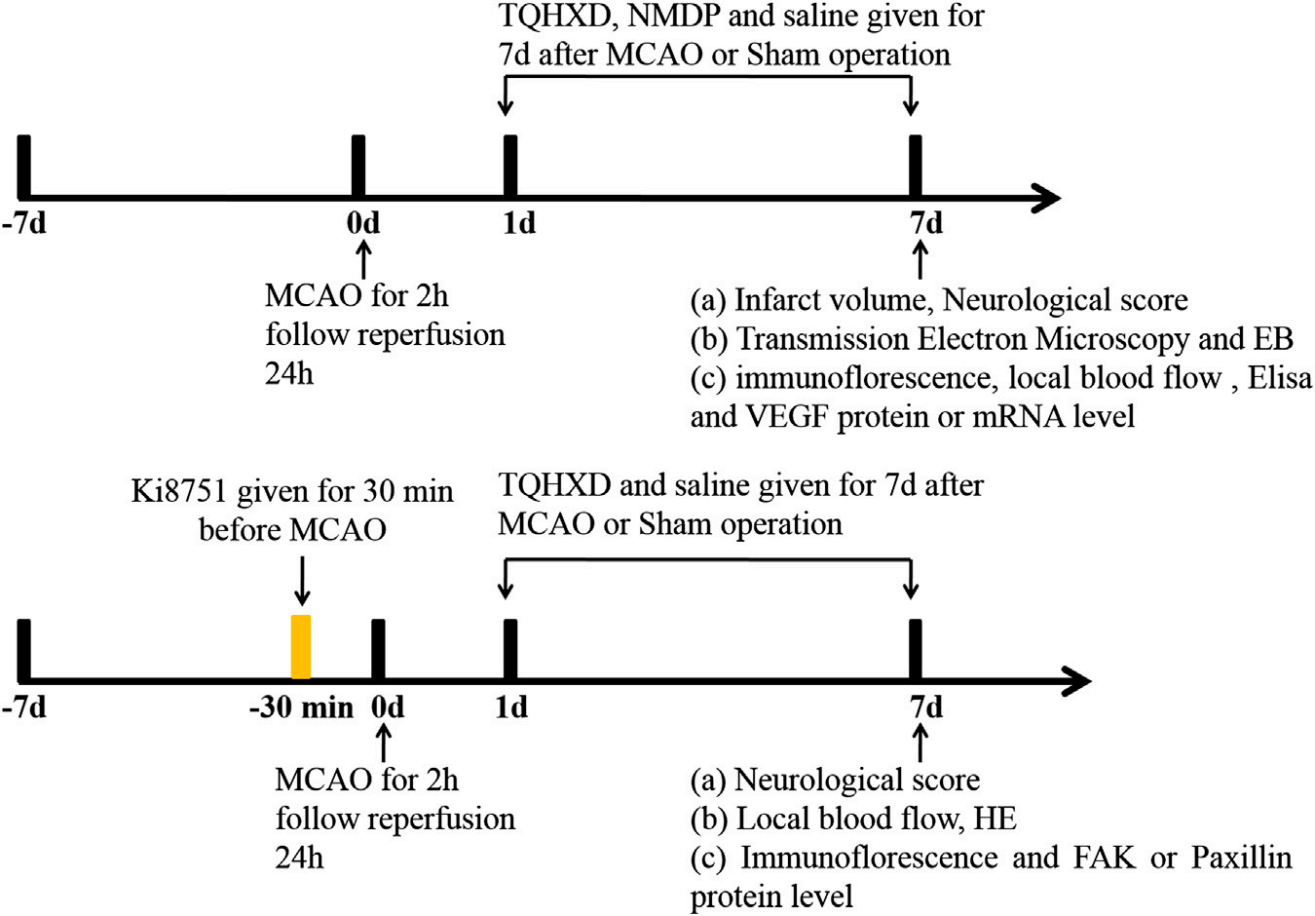
Experimental design and schedules. MCAO: middle cerebral artery occlusion; TQHXD: Tongqiao Huoxue Decoction; EB: Evans blue; ELISA: enzyme-linked immunosorbent assay; HE: hematoxylin-eosin staining; VEGF: vascular endothelial growth factor; FAK: focal adhesion kinase.

Secondly, to explore the mechanisms of TQHXD-associated angiogenesis (experiment 2), rats were randomly divided into five groups: the sham group, vehicle group, TQHXD group (6 g/kg), Ki8751 (0.5 mg/kg) group, and combine group (TQHXD + Ki8751). As mentioned in experiment 1, TQHXD and TQHXD + Ki8751 combine groups were treated with TQHXD by intragastric administration for 7 days. Rats in sham, vehicle, and Ki8751 groups were given the same volume of normal saline for 7 days. Tail vein injection Ki8751 (final dose: 0.5 mg/kg (Hou et al., 2015)) was administered to rats 30 min prior to operation (Figure 1).

### Neurological Score

Neurological recovery was examined at day 1 and day 7 after 24 h reperfusion using Longa’s method (Longa et al., 1989): 0, no neurologic deficit; 1, failure to extend forepaw fully; 2, circling to the left; 3, falling to the left or no spontaneous motor activity; 4, not walking spontaneously and experiencing a lowered stage of consciousness. Rats with scores of 0 and 4 were excluded from the experiment.

#### Monitoring Local Blood Flow Changes by Laser Doppler Flow

The rCBF of the left brain was measured by laser Doppler flow (LDF). Rats were anesthetized with 4% isoflurane; the skin and subcutaneous tissue were cut slowly. The fascia on the skull of rats was separated by nipper to fully expose the area of the skull. The position of assessment was recorded in the following coordinates relative to Bregma: ML: +2.0 mm, AP: +1.0 mm (Schmid-Elsaesser et al., 1998). Routine zero-calibration with front probe and monitoring the blood flow during the operation were performed. Finally, the blood flow was detected through LDF at days 1 and 7.

### Triphenyltetrazolium Chloride Staining

Rats were sacrificed at 30 min after the last drug administration; then frozen brains were sliced into coronal sections of 2 mm thickness. The slices were stained with 2% TTC (pH = 7.4, Sigma Chemical Co., United States) at room temperature. The change of cerebral infarct volume in all groups was analyzed by ImageJ. The infarct volume was calculated by infarct size * average slice thickness (2 mm).

### Immunofluorescence

Rats were sacrificed after the last drug treatment; brain tissues were isolated after being perfused with 0.9% normal saline followed by 4% cold paraformaldehyde. The brains were quickly frozen after gradient elution with sucrose and cut into 5 µm coronal thick slices. The sections were transparent with 0.5% Triton X-100 for 5 min and then blocked with 10% BSA for 1 h. Slices were incubated with primary anti-CD34 antibody (1:100, Abcam, United States) at 4 °C overnight. Then, the slices were washed with PBST 3 times. Slices were incubated with anti-rabbit secondary antibody (1:1,000, Cell Signal, United States) for 50 min at 37 °C. After counterstaining with 4, 6-diamidino-2-phenylindole (DAPI) and covering slices with anti-fluorescent quenching, the slices were observed and photographed under fluorescent microscopy (Leica, Germany) and then analyzed by Image-Pro 6.0 software.

#### Blood-Brain Barrier Permeability

The BBB permeability was determined by measuring the amount of Evans blue (EB). EB dye (2%, 4 ml/kg body weight) was slowly injected by tail vein injection intravenously 30 min after last drug treatment and allowed to circulate for 2 h. Rats were anesthetized with 4% isoflurane and then transcardially perfused with 0.9% normal saline to wash away any remaining dye in the blood vessels. The brain was incubated in 3 ml formamide at 45 °C for 72 h. The solutions were centrifuged for 40 min at 25,000 g. Finally, the absorption of each well was measured at 632 nm with a microplate reader (Thermo Scientific, United States). The content of EB in all groups was expressed as μg/g of the brain according to the standardized curve (Ren et al., 2015).

#### Hematoxylin and Eosin Staining

At 24 h after reperfusion, rats were anesthetized with 4% isoflurane and perfused transcardially with 0.9% saline and then reperfused with pre-ice cold 4% paraformaldehyde. The brain was embedded in paraffin after being dehydrated in graded ethanol and cleared in xylene. Finally, the slices of 4 µm sections were stained with hematoxylin and eosin (HE) staining following histochemical procedures.

#### Ultrastructural of Tight Junction Detection Through Transmission Electron Microscopy

In brief, rats were anesthetized with 4% isoflurane and then perfused with 0.9% normal saline, followed by precold 4% paraformaldehyde. The frontoparietal cortex of the ischemia brain was separated into pieces of 1 mm^3^ and then blended with 2.5% glutaraldehyde at 4 °C. Then, these pieces were dunked in 2% OsO_4_ for 2 h. The pieces were embedded in Epon 812 after being incubated with OsO_4_. Finally, ultrathin pieces of cortex were stained with citramalic acid and acetic acid uranium and then scanned by transmission electron microscopy (TEM).

#### Detection of Vascular Endothelial Growth Factor in Serum by Enzyme-Linked Immunosorbent Assay

At 30 min after the last drug treatment, rats were anesthetized with 4% isoflurane. Blood was collected from the abdominal aorta by a one-time negative pressure tube without anticoagulant. Blood was stood at room temperature for 3 h and then centrifuged at 2000 rpm for 20 min to collected serum. The expression of vascular endothelial growth factors (VEGF) in rats was detected using a VEGF ELSIA kit (RRV00. R&D Systems, Minneapolis, MN, USA). The absorbance of each well was measured at 450 nm through a microplate reader (Thermo Scientific, United States). The content of VEGF-A in the samples was calculated according to the standardized curve.

#### Western Blot

The protein levels of VEGF-A, FAK, Paxillin, and β-actin were detected using Western blot. Cortex tissues were lyzed with precold RIPA lysis buffer and immediately mixed with phenylmethanesulfonyl fluoride (PMSF, Beyotime, Nanjing, China) centrifuged at 12,000 rpm at 4 °C for 15 min. The concentration of protein was quantified by bicinchoninic acid (BCA) (Beyotime, Nanjing, China). All proteins were run on 12% (*w/v*) or 10% SDS-PAGE gel and then transferred to polyvinylidene difluoride (PVDF) membrane with Bio-Rad electrophoretic transfer system. The membrane was blocked with 5% (*w/v*) nonfat milk for 2 h at 37 °C and incubated with the primary antibody to VEGF-A (1: 800), p-FAK (1: 1,000), Paxillin (1: 1,000), and β-actin (1: 1,000) at 4 °C overnight, respectively. After washing with Tris-Hcl Buffered Saline containing 0.1% Tween-20 (TBS-T) 3 times, the membrane was incubated with coherent secondary (1:5,000) antibody for 2 h at 37 °C. β-Actin was used as an internal reference. The protein bands were developed using enhanced chemiluminescence (ECL) reagents (Thermo, United States). Finally, images were scanned by an Amersham Imager 600 (GE, United States).

#### Quantitative Real-Time PCR

In brief, RNA was isolated using a tissue RNA kit (Omega Bio-Tek, GA) and then was reverse-transcribed using a TAKARA reverse transcription kit. Real-time quantitative PCR (qRT-PCR) was performed on the Mx3000P qPCR System (Agilent Technologies, Santa Clara, CA, United States) using SYBR Premix Ex Taq TM (TAKARA). Primer sequences used to amplify fragments were synthesized by Sangon Biotech, Shanghai. The relative mRNA expression was normalized to GAPDH using the relative quantification method 2^−ΔΔCt^. The sequences of primers in this experiment were as follows: GAPDH Forward, 5-GGG​TGT​GAA​CCA​TGA​GAA​GTA​TG-3, GAPDH Reverse, 5-GAT​GGC​ATG​GAC​TGT​GGT​CAT-3; VEGF Forward, 5-CTG​CCA​TCC​AAT​CGA​GAC​CC-3, VEGF Reverse, 5-TGC​ATT​CAC​ATT​TGT​TGT​GCT​G-3.

### Statistical Analysis

The Shapiro–Wilk normality test was used to analyze whether the data were normally distributed. Then, the *t*-test was utilized to analyze the significance of differences. Statistical significance between groups was evaluated by one-way analysis of variance (ANOVA). All data were presented as mean ± standard deviation (SD). Statistical analysis was performed using SPSS (SPSS, Inc., version20.0). A significant difference was accepted at *p* values lower than 0.05.

## Results

### Genes Associated with Ischemia-Reperfusion

A total of 929 significant genes were obtained from the GeneCards, OMIM, and DrugBank databases after screening out some targets with low credibility (Supplementary Table S1).

### Drug Targeting

According to the ADME thresholds of OB ≥ 30% and DL ≥ 0.18, 94 compounds of TQHXD were obtained from the three following databases: TCMSP, TCM Database@Taiwan, and TCMID after eliminating the common compounds, as listed in Supplementary Table S2. In addition, we also achieved quality control, that is, the HPLC system of herbs in TQHXD, such as precision, stability, repeatability, and wavelength, as shown in Supplementary Table S3. More importantly, some compounds with low OB or HL values but with significant protective effects on I/R injury, such as ligustrazine, muscone, and hydroxysafflor yellow A, were also explored in our work (Wu et al., 2018; Li et al., 2017).

### Functional Enrichment Analysis of Intersection Targets of Tongqiao Huoxue Decoction

**FIGURE 2:**
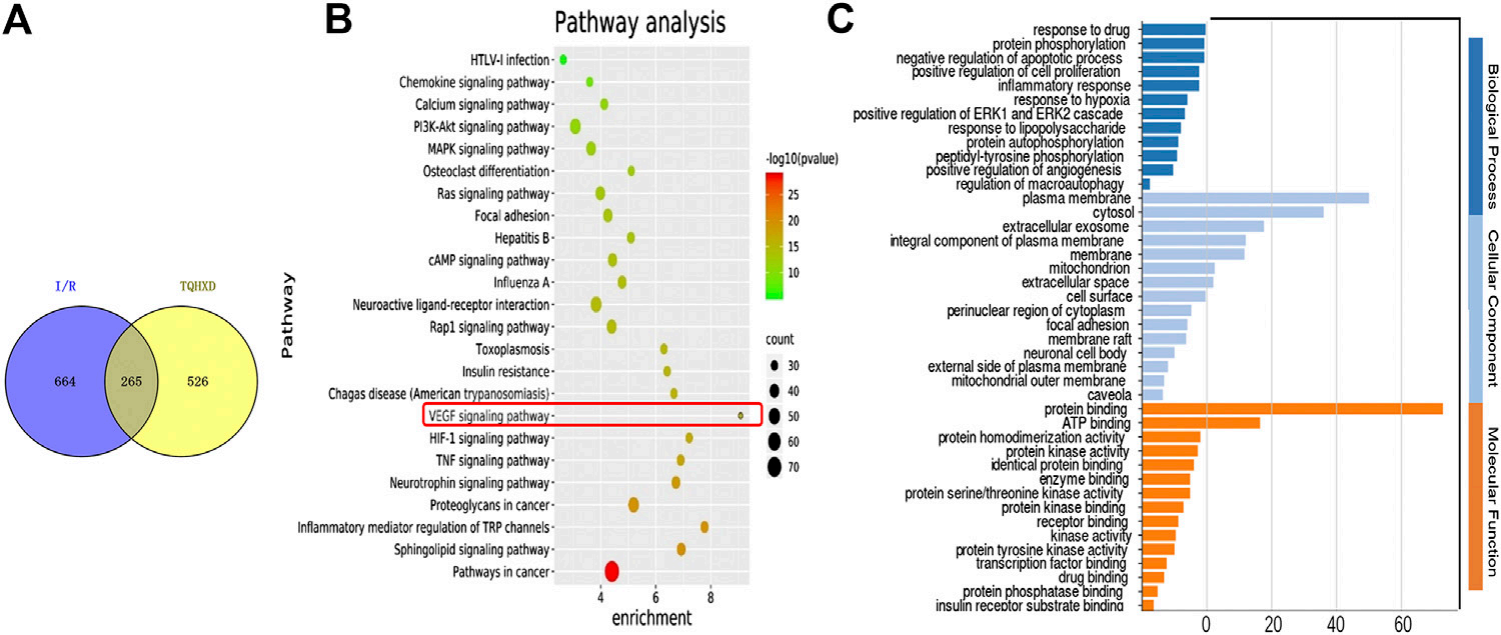
Network pharmacology analysis of the ingredients of TQHXD. **(A)** Common targets between TQHXD and targets associated with I/R. **(B,C)** GO enrichment analysis of 265 common targets.

A total of 265 matching targets as the related targets of I/R and TQHXD are shown in Figure 2A. The multiple biological senses of TQHXD on I/R from a systematic level were assessed using the GO enrichment analysis (Figure 2C). For biological processes, potential targets of TQHXD were remarkably enriched in “response to hypoxia” (GO: 0001666), protein phosphorylation (GO: 0006468), and inflammatory response (GO: 0006954). For cellular components, mainly, plasma membrane (GO: 0005886), cytosol (GO: 0005829), and extracellular exosome (GO: 0070062) were enriched. Subsequently, the crucial pathways among the 265 potential targets in TQHXD treatment were also elucidated in this work. The threshold of FDR <0.01 was used as an enrichment screening criterion, and the top 24 pathways were screened out (Figure 2B), including the VEGF signaling pathway (hsa04370) and TNF signaling pathway (hsa04668).

### PPI Network and Disease Analysis of Intersection Targets of Tongqiao Huoxue Decoction

**FIGURE 3:**
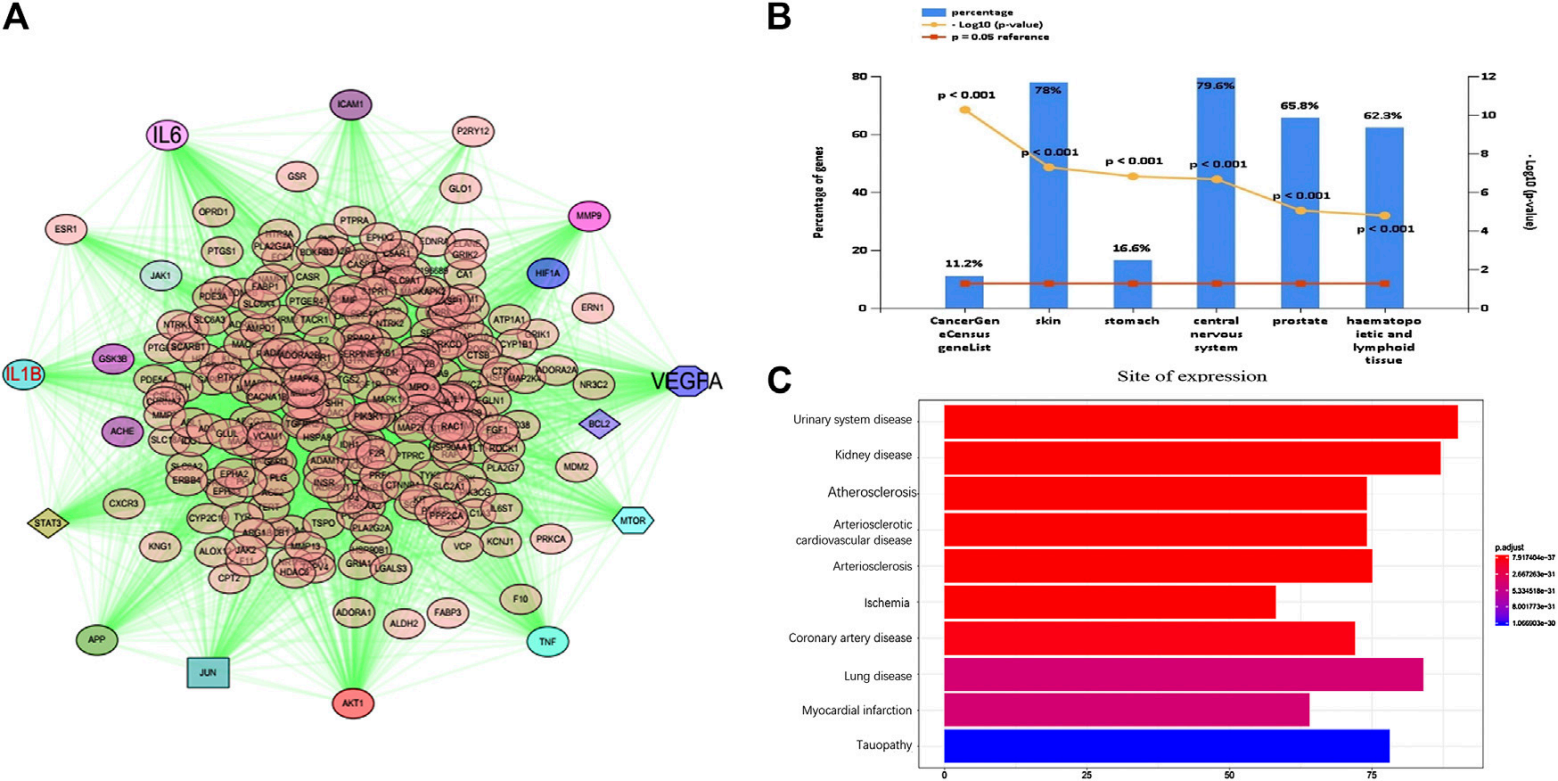
PPI network and disease analysis of intersecting target of TQHXD. **(A)** PPI network of 265 common targets. **(B,C)** Clinical synopsis phenotypic terms.

Furthermore, the PPI network of those common targets was found in Figure 3 (Figure 3A). Ultimately, TNF, AKT1, IL-6, VEGF-A, JUN, EGFR, IL1B, IGF1, MMP9, and TGFB1 were identified as major nodes. Similarly, we analyzed the diseases of these targets. For the site of expression, the targets were enriched in the central nervous system and skin, etc. Finally, we used the disease analysis function of the NIMNT database and entered 265 intersection targets of TQHXD to enrich the possible indications of TQHXD. After setting p.adjust <0.001, the top 10 diseases were observed, as shown in Figure 3, which mainly include ischemia, urinary system disease, and arteriosclerotic cardiovascular disease (Figure 3C).

### The Quality Control of Tongqiao Huoxue Decoction by HPLC

**FIGURE 4:**
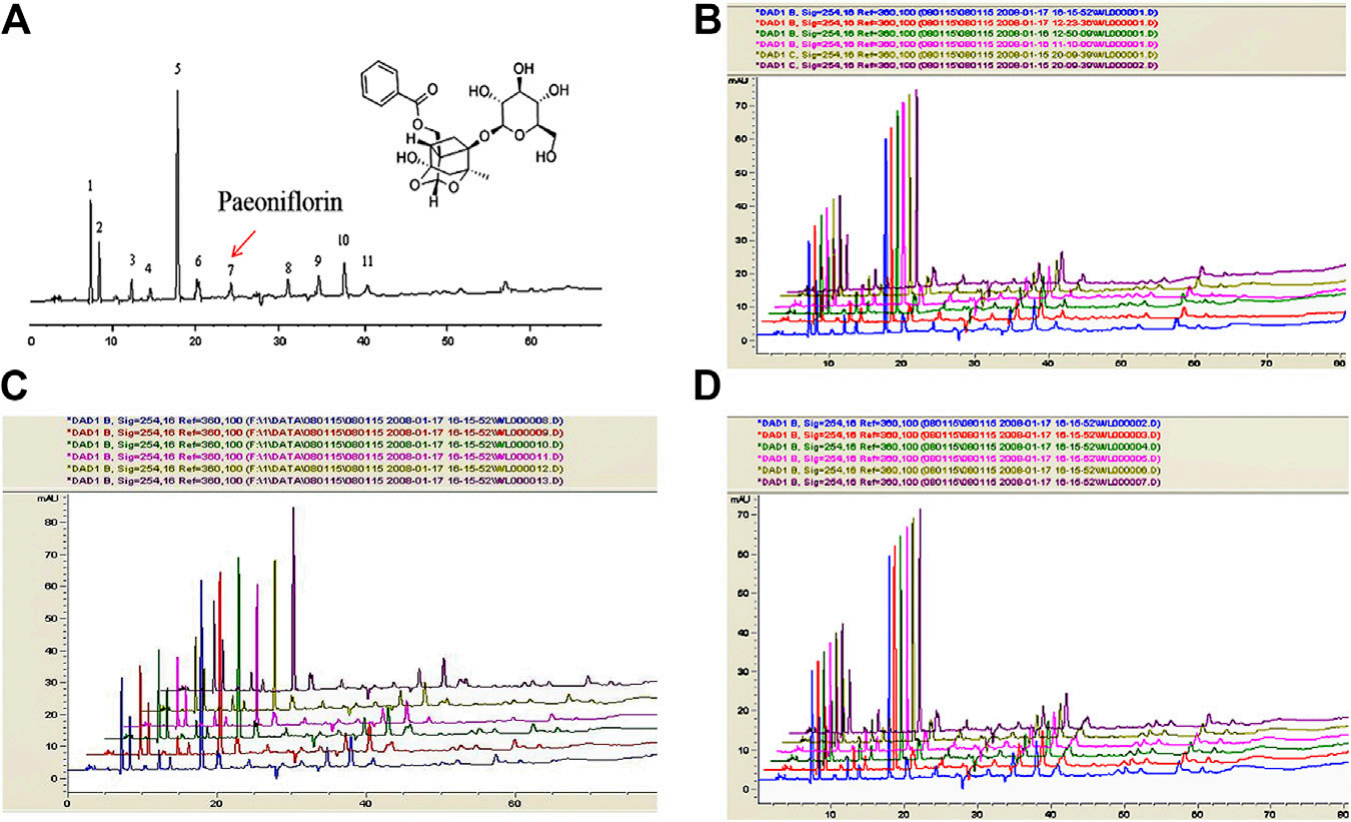
The fingerprints of TQHXD for quality control by HPLC. Conditions: column, C18 column (250 × 4.6 mm, 5 μm); mobile phase, methanol. **(A)** H_2_O (0.05% V/V phosphoric acid). Flow rate: 1 ml min^−1^; column temperature: 25 °C; injection volume: 10 μL. **(A)** Representative HPLC chromatogram of TQHXD. 7, Paeoniflorin. **(B–D)** The quality control of six batches of TQHXD evaluated by HPLC. The TQHXD solution from six separated raw crude extractions was analyzed by the HPLC method. Colors represent different groups of TQHXD test solutions. The *X*-axis represents acquisition time (Min) and the *Y*-axis indicates intensity (mAU).

As shown in Figure 4A, the retention time of paeoniflorin of TQHXD was about 24.2 min. Subsequently, the HPLC method was validated by reproducibility, stability, and precision. It is worth noticing that there was an identical chromatographic pattern in six batches of TQHXD solutions. This suggested a good reproducibility for the HPLC system or TQHXD solutions. Finally, the relative standard deviation (RSD) of the instrument was less than 3%, indicating that the precision of the instrument was good. Similarly, the RSDs of relative retention time and relative peak area of each characteristic peak were less than 5%, indicating that it has good repeatability. The RSDs of relative retention time and relative peak area of characteristic peaks were less than 5% after 0, 2, 4, 8, 12, and 24 h injection, respectively, indicating that the solution of the test sample was stable within 24 h (Figure 4B–D, Supplementary Table S3).

### Tongqiao Huoxue Decoction Ameliorated Ischemia-Reperfusion Injury and Attenuated Blood-Brain Barrier Damage

**FIGURE 5:**
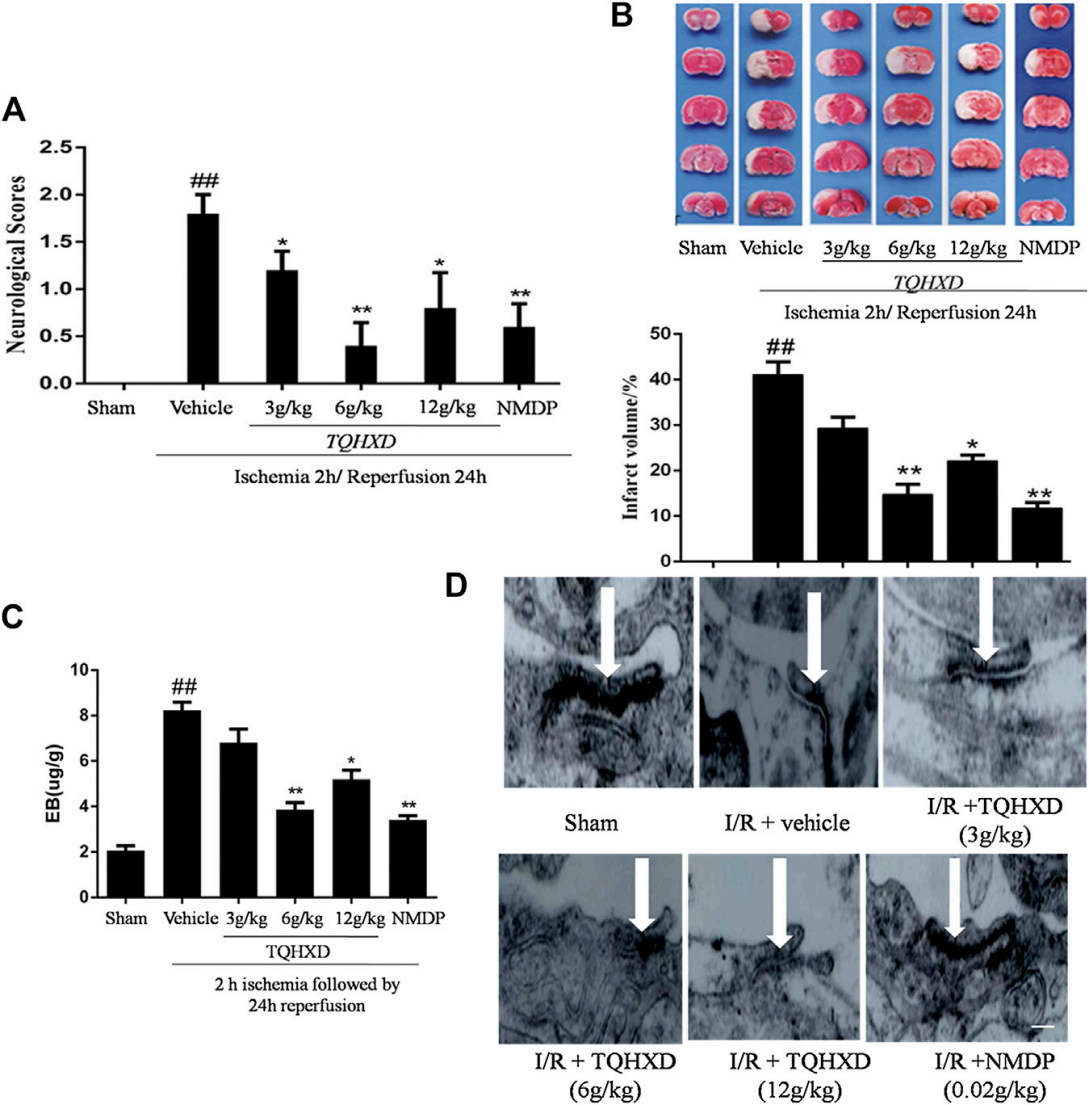
TQHXD ameliorated I/R injury and attenuated blood-brain barrier damage. **(A)** The neurological scores were evaluated by Longa’s methods. **(B)** Representative TTC staining **(top)** and quantitative analysis **(bottom)** of brain infarct volumes, *n* = 6. **(C)** Quantitative analysis of the content of EB, *n* = 6. **(D)** Representative TEM images of ultrastructural changes in I/R-induced tight junction (*n* = 3). *Arrows* imply electronically dense bands; the electronically dense bands represent TJ; the open state of electronically dense bands represents the injury degrees of TJ; scale bar = 200 μm (×20,000 magnification). Data are presented as means ± SD. ^##^ *p* < 0.01 vs. sham group; *p < 0.05, **p < 0.01 vs. vehicle group.

Neurological scores remarkably increased in the vehicle group compared with the sham group at 7 days. After reperfusion, treatment with TQHXD or NMDP showed significant neurological recovery, characterized by a decrease in neurological scores, compared with that in the vehicle group; however, TQHXD (6 g/kg) reduced the score compared to NMDP. These data showed that TQHXD might exert an effect on improving the neural function of MCAO rats (Figure 5A).

Infarct volume was not observed in brain slices of the sham group, whereas a large area of infarct volume (41.10%) was seen in the vehicle group. The infarct volumes in the TQHXD groups were 29.3, 14.83, and 22.20%, respectively. Furthermore, infarct volume in the NMDP group (11.79%) was close to that observed in the TQHXD group at the dose of 6 g/kg, compared with the vehicle group (Figure 5B).

The protective effect of TQHXD on the permeability of BBB was quantified by EB dye (Figure 5C). On the dye solution, we found that there was a remarkable (*p* < 0.01) increase in BBB permeability in rats from the vehicle group compared to that in sham rats. TQHXD (3, 6, and 12 g/kg) or NMDP (0.02 g/kg) reduced the content of EB under I/R. The treatment of TQHXD (6 g/kg) and NMDP made a significant difference to the BBB permeability when compared with the vehicle group (*p* < 0.01). Subsequently, we also detected the situation of tight junctions (TJ) by TEM. The TJ were normal in the sham group, whereas they were in an open state in the vehicle group (Figure 5D). However, different degrees of TJ reduction were observed in the TQHXD or NMDP group, indicating that TQHXD might repair the poststroke TJ completeness.

### Tongqiao Huoxue Decoction Strengthened Angiogenesis in MCAO/R Rats

**FIGURE 6:**
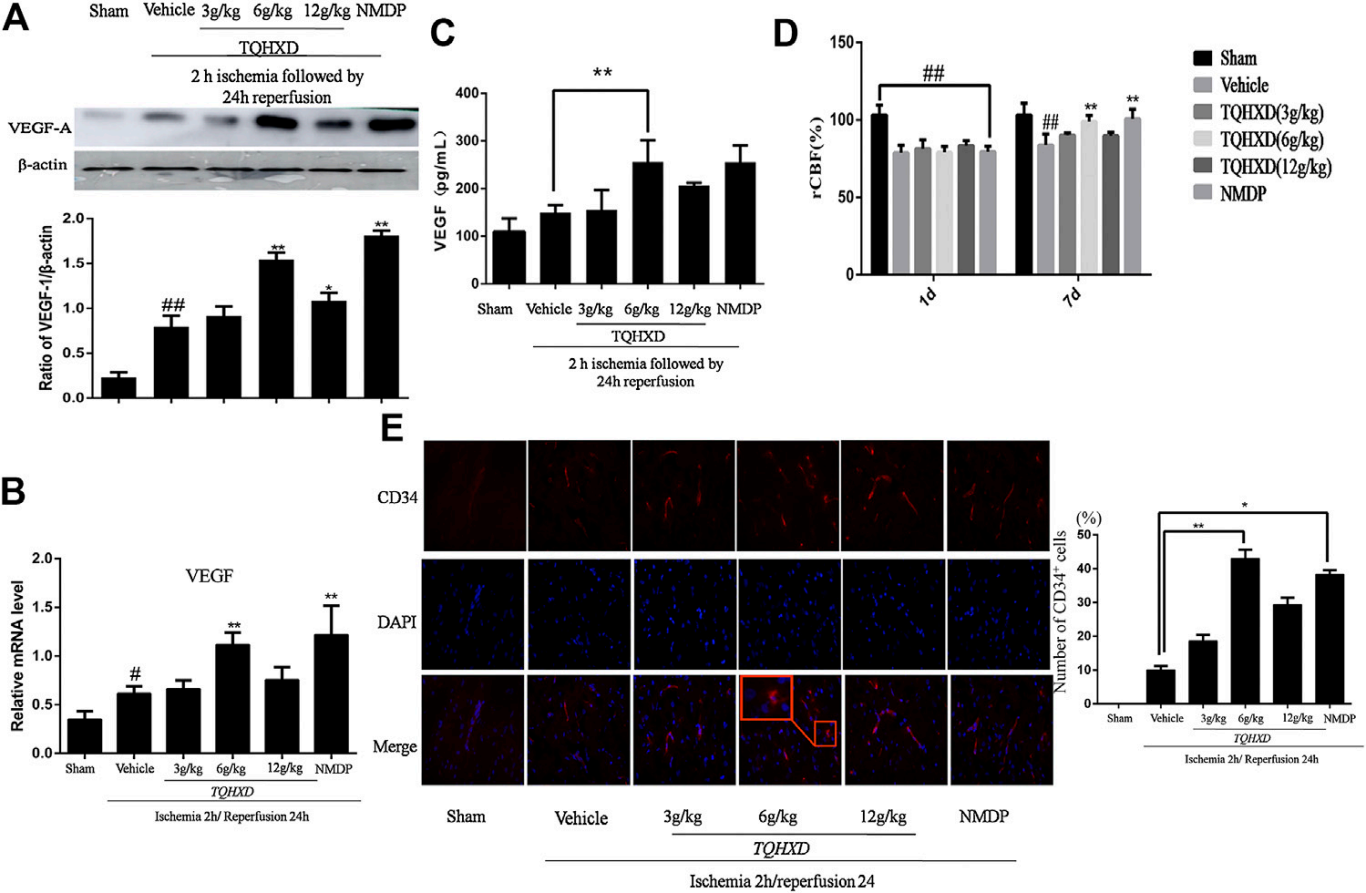
TQHXD strengthened angiogenesis in MCAO rats. **(A)** Representative band image **(top)** and quantitative analysis **(bottom)** of VEGF-A (*n* = 3). **(B)** The mRNA expression of VEGF in cortex detected by qPCR. **(C)** Quantitative analysis for ELISA results of VEGF at 7 days. **(D)** The changes of rCBF for all groups at 1 and 7 d after reperfusion, *n* = 3. **(E)** Immunofluorescence of brain section. Angiogenesis was visualized by CD34 staining (red), and DNA was stained by 4′6,-diamidino-2-phenylindole (DAPI; blue). *Red box* implies zoom-in fluorescence. Right, the histograms indicate the percentages of CD34-expressing cells that were positive for indicated markers. Scale bar = 50 μm (×200 magnification). Data are presented as means ± SD. ^##^ *p* < 0.01 vs. sham group; ^*^ *p* < 0.05, ^**^ *p* < 0.01 vs. vehicle group.

The data of Western blot and ELISA demonstrated that an increased expression of VEGF exposed to I/R as compared to the sham group. Then, the TQHXD or NMDP treatment further upregulated the expressions of VEGF-A in both serum and cortex tissue, as compared with the vehicle group, which showed that TQHXD could be involved in angiogenesis. Finally, qPCR analysis of VEGF mRNA expression provided further proof of the effects of TQHXD on angiogenesis (Figure 6).

The cerebral blood flow was reduced rapidly to 30% during ischemia after 2 h and then was restored to 80% of the baseline after reperfusion. The rCBF showed that blood flow had no significant difference in the sham group between the time points of 1 and 7 days. The rCBF of the vehicle group or drug groups was lower than that of the sham group at day 1 (*p* < 0.01). Similarly, the rCBF of the vehicle group rats had no significant difference at 7 days, as compared to the sham group. Instead, compared with the vehicle group, TQHXD (6 g/kg) and NMDP groups significantly increased the rCBF at 7 days (Figure 6D).

Subsequently, we used CD34 immunofluorescence to mark the MVD at 7 days after TQHXD treatment. In the vehicle group, MVD increased at 7 days after I/R when compared with the sham group. More importantly, the expression of CD34 in the TQHXD group was significantly higher than that in the sham and vehicle groups (Figure 6E).

### Exogenous VEGFR2 Inhibition Reversed Angiogenesis in Tongqiao Huoxue Decoction

**FIGURE 7:**
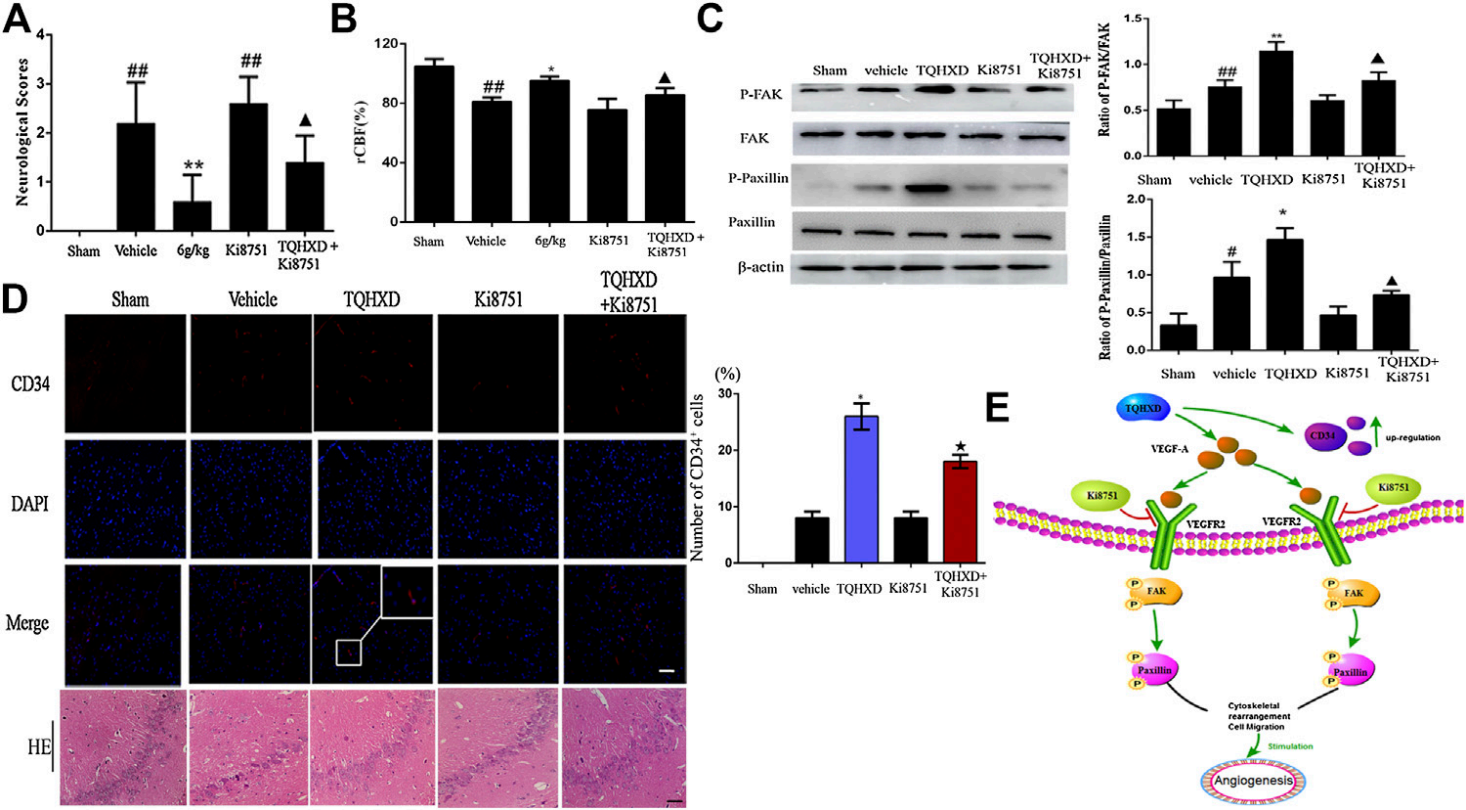
TQHXD enhanced I/R-induced angiogenesis via activation of VEGF-A/VEGFR2-FAK-Paxillin signaling. **(A)** Neurological scores measured by Longa’s method (*n* = 5). **(B)** The changes of rCBF for all groups at 7 days after reperfusion, *n* = 6. **(C)** Representative Western blot band **(top)** and quantitative analysis **(bottom)** of the expressions of FAK, p-FAK, Paxillin, and p-Paxillin (*n* = 3). β-Actin was used as an internal reference. **(D)** Left, serial sections of the brain were subjected to hematoxylin and eosin (HE) staining, immunofluorescence for angiogenesis markers (CD34, red), and DNA was stained by 4′6-diamidino-2-phenylindole (DAPI; blue). *White box* implies zoom-in fluorescence. Right, the histograms indicate the percentages of CD34-positive cells. Scale bar = 50 μm (×200 magnification). *White box* implies zoom-in fluorescence. **(E)** The model illustrates the molecular mechanism of TQHXD on angiogenesis in MCAO injury rats. Data were expressed as mean ± SD. ^##^ *p* < 0.01 vs. sham group; *p < 0.05, **p < 0.01 vs. vehicle group; ▲, ★*p* < 0.05 vs. TQHXD (6 g/kg) group.

To further explore the molecular mechanisms associated with the phenotype observed upon TQHXD administration, we profiled angiogenesis from rats after being injected with Ki8751 (a specific VEGFR2 inhibitor). Firstly, we investigated whether Ki8751 could reverse the effect of TQHXD on neurological function score and cerebral blood flow. Notably, the neurological and neuronal death and rCBF were substantially protected by the TQHXD treatment in MCAO rats (Figures 7A,B,D). Subsequently, as shown in Figure 7C, the expression of p-FAK and p-Paxillin proteins in the cortex of brain induced by I/R was greater than that in the sham group (*p* < 0.05). However, TQHXD (6 g/kg) remarkably promoted the expression of p-FAK and p-Paxillin proteins (*p* < 0.01 vs. vehicle group) (Figure 7C). Conversely, the treatment of rats with VEGFR2 inhibitors, such as Ki8751, substantially reduced the TQHXD-induced angiogenesis. For example, the upregulating effect of TQHXD on p-FAK or p-Paxillin was impeded by the cotreatment with Ki8751 (*p* < 0.05). Similarly, the expression of CD34 induced by TQHXD was also inhibited by Ki8751. These results demonstrated that TQHXD enhanced angiogenesis in a VEGFR2-FAK-Paxillin-dependent manner.

## Discussion

In the present study, a total of 94 potential compounds were screened according to OB and DL. The results revealed that TQHXD regulated the intersection of 265 targets with I/R, including VEGF-A, IL6, JUN, mTOR, TNF, and AKT1. These 265 targets were mainly enriched in cancer, VEGF, and neurotrophin signaling pathways. To ascertain TQHXD’s role, our results of the network indicated that TQHXD regulated I/R through the pattern multiple components-multiple targets-multiple pathways. However, given the importance of angiogenesis in I/R, we linked the role of TQHXD with angiogenesis by the results of network pharmacology and enrichment analysis of the KEGG pathway.

A huge percentage of elderly people reportedly suffer from ischemic stroke worldwide. Neurovascular unit (NVU), a conceptual model of stroke depending on the structure of blood vessels and brain, has been proposed by the National Institutes of Neurological Disorders and Stroke (Zhao et al., 2017). Previous studies have shown that angiogenesis is a key process in NVU recovery following ischemia stroke. This process leads to restoration of rCBF after cerebral ischemia and redounds to the neurological recovery of patients with stroke (Liu et al., 2017). In fact, recent research shows that patients with angiogenesis exhibit lower morbidity and mortality rates relative to those without the condition (Ducruet et al., 2010). In the present study, we show that TQHXD is a key mediator of angiogenesis. Specifically, our results show that the amount of blood flow in ischemic brain tissue is the most intuitive indicator for evaluating the maturity of neovascularization. We found no significant increase in rCBF in the MCA and cortical regions in rats from the sham group. In contrast, rats treated with TQHXD or NMDP exhibited significantly higher CD34 or rCBF levels 7 days after reperfusion, compared to those in the vehicle group.

Ischemic injury causes endothelial progenitor cells to participate in the repair of new blood vessels. In fact, numerous cytokines reportedly play a critical role in this process, with a combination of these factors found to promote angiogenesis. Previous studies had shown that under specific environmental conditions, brain neurovascular cells produced and oozed VEGF, a central angiogenesis mediator following stroke (Yin et al., 2015). VEGF was upregulated both *in vivo* and *in vitro* during the I/R or oxygen-glucose deprivation induced by ischemia injury and continued 3–14 days poststroke (Zheng et al., 2018; Liu et al., 2018). This expression pattern indicates that ischemic injury induces angiogenesis, which was enhanced by TQHXD, to repair damaged blood vessels after stroke. In the present study, a significantly higher VEGF expression was observed in the TQHXD, compared to the vehicle group (*p* < 0.05), with a concentration of 6 g/kg generating the highest levels. These results strongly indicated that TQHXD protects against I/R injury by upregulating angiogenesis either directly or indirectly.

Compared with NMDP, our results showed the therapeutic effects of TQHXD on the MCAO model. Notably, upon TQHXD administration, remarkably better levels of the neurological score and MVD were observed in the TQHXD group than those in the NMDP groups. These results illustrated that the nerve recovery activity of TQHXD was better than that of NMDP. As we all know, nerve recovery is a complex process involved in multiple-target and multiple-pathway. Compared with a single target drug (NMDP), the multitarget advantage of TQHXD could effectively regulate multiple processes to accelerate nerve recovery, which was also in agreement with our network pharmacology results. More importantly, despite the encouraging progress to apply NMDP in clinical trials, various adverse side-effects associated with NMDP may limit its clinical application. The relevant example is a reduction in blood pressure and a reversible increase in the serum concentrations of liver enzymes during intravenous therapy. On the contrary, compared with chemical medicines, TCMs are considered to be safe, with fewer side-effects. As such, the established safety and drug tolerability are the advantages of TCM over several Western medicines, which is exactly also the biggest benefit of TCM. Interestingly, our results showed that TQHXD’s protective effect on I/R was not in a dose-dependent manner. Generally speaking, the effect of drugs may show a dose-dependent manner; previous studies had shown that the protective effect of some classical TCM does not always follow this trend (Yang et al., 2013; Xin-Xin et al., 2015). For example, some chemical and physical reactions may occur during the preparation of the formula, resulting in changes in some active components, which may not reflect dose-dependency (Xu et al., 2008). Herein, in-depth studies are required to elucidate the mechanisms of TQHXD action to provide valuable insights into its effects on I/R.

Previous studies have shown that VEGF combines with its receptors to promote angiogenesis against I/R (Beck and Plate, 2009; Greenberg and Jin, 2005). Specifically, the VEGF signaling pathway plays a fundamental role in angiogenesis response by regulating the release of growth factors. VEGFR2 regulates the expression of vascular endothelial cell receptor proteins, thereby playing a critical role in the development of many diseases mediated by vascular endothelial cells. Notably, FAK is considered the key signaling molecule in cells and has been reported to regulate cell proliferation, migration, spreading, adhesion, and key processes in angiogenesis (You et al., 2014).In addition, Paxillin, a key downstream FAK protein that acts on adhesion plaque, has been found to mediate the formation of adhesion plaque, activate downstream proteins, and promote angiogenesis. Some studies have shown its phosphorylation directly affects the formation of adhesion plaques and ultimately affects the motility of endothelial cells (Blackstone et al., 2015). In addition, blocking FAK phosphorylation can significantly reduce the degree of angiogenesis (Masakazu et al., 2010). To determine whether TQHXD provokes angiogenesis through the VEGF machinery, we evaluated the effect of TQHXD with Ki8751, a potent VEGFR2 inhibitor known to block angiogenesis (Lu et al., 2017). Our results indicated that I/R-induced angiogenesis increased CD34 expression and decreased neuronal death in rats. However, TQHXD treatment promoted this effect. In addition, Ki8751 decreased the levels of FAK and Paxillin expression, with its Ki8751 treatment clearly attenuating TQHXD-induced angiogenesis. Overall, these results indicate that TQHXD confers protection against I/R in rats via angiogenesis by activating the VEGF-A/VEGFR2-FAK-Paxillin signaling pathway.

Although network pharmacology has revealed the effects of TQHXD, there are still some limitations to be addressed. First, the biggest challenge is the results from the database on Chinese herbs. The public database information on herbal medicine remains incomplete to some extent. Subsequently, in order to further substantiate the idea that TQHXD provokes angiogenesis through promoting the VEGF machinery, we could test whether the angiogenesis effect of TQHXD could be blocked by shVEGF or hVEGF-IN-1 (VGEF inhibitor), a potent VEGF interference known to block VEGF. Finally, although our study shows that TQHXD has an impact on angiogenesis, our results are not capable of distinguishing which angiogenesis cell types are responsible. Angiogenesis cells can be labeled in a specific manner, and this issue will be addressed in future research.

## Conclusion

In conclusion, the present study revealed that TQHXD administration was able to ameliorate the ischemic stroke of MCAO rats through promoting angiogenesis to active the VEGF-A/VEGFR2-FAK-Paxillin pathway.

## Data Availability Statement

The raw data supporting the conclusions of this article will be made available by the authors, without undue reservation, to any qualified researcher.

## Ethics Statement

The animal study was reviewed and approved and animal experiments were approved by the Animal Care and Use Committee of Anhui University of Chinese Medicine, China, and implemented in accordance with the Guidelines of the National Institutes of Health on the Care and Use of Animals.

## Author Contributions

SW and NW conceived the project and designed research. SW and LZ performed the experiments and wrote the manuscript. NW and SW revised the manuscript.

## Funding

The study was supported by the National Natural Science Foundation of China (Nos. 81773933 and 30973979) and academic assistance program for the top-notch innovative talents from universities in 2017 provided by Anhui Province Office of Education (gxbjZD15).

## Conflict of Interest

The authors declare that the research was conducted in the absence of any commercial or financial relationships that could be construed as a potential conflict of interest.

## References

[B1] Beck H. Plate K. H. (2009). Angiogenesis after cerebral ischemia. Acta Neuropathol. 117, 481–496. 10.1007/s00401-009-0483-6 19142647

[B2] Benjamin E. J. Virani S. S. Callaway C. W. Chang A. R. Muntner P. (2018). Heart disease and stroke statistics—2018 update: a report from the American heart association. Circulation, 137, e67–e492. 10.1161/CIR.0000000000000558 29386200

[B3] Blackstone B. N. Li R. Ackerman W. E. Ghadiali S. N. Powell H. M. Kniss D. A. (2015). Myoferlin depletion elevates focal adhesion kinase and paxillin phosphorylation and enhances cell-matrix adhesion in breast cancer cells. Am. J. Physiol. Cell Physiol. 308, C642 10.1152/ajpcell.00276.2014 25631868

[B4] Carmeliet P. (2000). Mechanisms of angiogenesis and arteriogenesis. Nat. Med. 6, 389–395. 10.1038/74651 10742145

[B5] Ge C.-L. Wang X.-M. Huang Z.-G. Xia Q. Wang N. Xu D.-J. (2015). Tongqiao Huoxue Decoction ameliorates learning and memory defects in rats with vascular dementia by up-regulating the Ca(2+)-CaMKII-CREB pathway. Chin. J. Nat. Med. 13, 823–830. 10.1016/S1875-5364(15)30086-8 26614457

[B6] Chen C. Venketasubramanian N. Gan R. N. Lambert C. Picard D. Chan B. P. (2009). Danqi piantang jiaonang (DJ), a traditional Chinese medicine, in poststroke recovery. Stroke 40, 859–863. 10.1161/STROKEAHA.108.531616 19164787

[B7] Chen F. P. Chang C. M. Hwang S. J. Chen Y. C. Chen F. J. (2014). Chinese herbal prescriptions for osteoarthritis in Taiwan: analysis of National Health Insurance dataset. BMC Compl. Alternative Med. 14, 91 10.1186/1472-6882-14-91 PMC3973832 24606767

[B8] Ducruet A. F. Gigante P. R. Hickman Z. L. Zacharia B. E. Arias E. J. Grobelny B. T. (2010). Genetic determinants of cerebral vasospasm, delayed cerebral ischemia, and outcome after aneurysmal subarachnoid hemorrhage. J. Cerebr. Blood Flow Metabol. 30, 676 10.1038/jcbfm.2009.278 PMC2949164 20068580

[B9] Ferrara N. Kerbel R. S. (2005). Angiogenesis as a therapeutic target. Nature 438, 967–974. 10.1038/nature04483 16355214

[B10] Greenberg D. A. Jin K. (2005). From angiogenesis to neuropathology. Nature 438, 954–959. 10.1038/nature04481 16355213

[B11] Hopkins A. L. (2008). Network pharmacology: the next paradigm in drug discovery. Nat. Chem. Biol. 4, 682–690. 10.1038/nchembio.118 18936753

[B12] Hou S. T. Nilchi L. Li X. Gangaraju S. Jiang S. X. Aylsworh A. (2015). Semaphorin3A elevates vascular permeability and contributes to cerebral ischemia-induced brain damage. Sci. Rep. 5, 7890 10.1038/srep07890 25601765 PMC4298747

[B13] Kim S. H. Park H. S. Hong M. J. Yoo J. Y. Lee H. Lee J. A (2016). Tongqiaohuoxue decoction ameliorates obesity-induced inflammation and the prothrombotic state by regulating adiponectin and plasminogen activator inhibitor-1. J. Ethnopharmacol. 192, 201–209. 10.1016/j.jep.2016.07.023 27404230

[B14] Krupinski J. Kaluza J. Kumar P. Kumar S. Wang J. M. (1994). Role of angiogenesis in patients with cerebral ischemic stroke. Stroke 25, 1794–1798. 10.1161/01.str.25.9.1794 7521076

[B15] Li L. Wang N. Jin Q. Wu Q. Liu Y. Wang Y. (2017). Protection of tong-qiao-huo-xue decoction against cerebral ischemic injury through reduction blood-brain barrier permeability. Chem. Pharm. Bull. 65, 1004–1010. 10.1248/cpb.c17-00267 29093286

[B16] Liu C. Zhou L. Shui Z. (2003). Tongqiao huoxue tang and buyang huanwu tang for treatment of vascular dementia—a report of 36 cases. J. Tradit. Chin. Med. 23, 243–245. 14719286

[B17] Liu J. Li Q. Zhang K.-S. Hu B. Niu X. Zhou S.-M. (2017). Downregulation of the long non-coding RNA Meg3 promotes angiogenesis after ischemic brain injury by activating notch signaling. Mol. Neurobiol. 54, 8179–8190. 10.1007/s12035-016-0270-z 27900677 PMC5684256

[B18] Liu J. Wang Y. Akamatsu Y. Lee C. C. Stetler R. A. Lawton M. (2014). Vascular remodeling after ischemic stroke: mechanisms and therapeutic potentials. Prog. Neurobiol. 115, 138–156. 10.1016/j.pneurobio.2013.11.004 24291532 PMC4295834

[B19] Liu Y. Li Y. Zhan M. Liu Y. Li Z. Li J. (2018). Astrocytic cytochrome P450 4A/20-hydroxyeicosatetraenoic acid contributes to angiogenesis in the experimental ischemic stroke. Brain Res. 1708, 84–70. 10.1016/j.brainres.2018.12.023 30571981

[B20] Longa E. Z. Weinstein P. R. Carlson S. Cummins R. (1989). Reversible middle cerebral artery occlusion without craniectomy in rats. Stroke 20, 84 10.1161/01.str.20.1.84 2643202

[B21] Lu X. Horner J. W. Paul E. Shang X. Troncoso P. Deng P. (2017). Effective combinatorial immunotherapy for castration-resistant prostate cancer. Nature 543, 728–732. 10.1038/nature21676 28321130 PMC5374023

[B22] Masakazu H. Masahiro O. Yuji K. Yushi I. (2010). Sequential activation of RhoA and FAK/paxillin leads to ATP release and actin reorganization in human endothelium. J. Physiol. 558, 479–488. 10.1113/jphysiol.2004.065334 PMC1664968 15155793

[B23] Mou X. Zhou D. Y. Zhou D. Liu K. Chen L. J. Liu W. H. (2020). A bioinformatics and network pharmacology approach to the mechanisms of action of Shenxiao decoction for the treatment of diabetic nephropathy. Phytomedicine 69, 153192 10.1016/j.phymed.2020.153192 32200292

[B24] Mozaffarian D. Benjamin E. J. Go A. S. Amett D. K. Blaha M. J. Cushman M. (2015). Heart disease and stroke statistics—2015 update: a report from the American heart association. Circulation 131, e29–322. 10.1161/CIR.0000000000000152 25520374

[B25] Pan L. Li Z. Wang Y. Zhang B. Lu G. Liu J. (2020). Network pharmacology and metabolomics study on the intervention of traditional Chinese medicine Huanglian Decoction in rats with type 2 diabetes mellitus. J. Ethnopharmacol. 258, 112842 10.1016/j.jep.2020.112842 32333952

[B26] Peng T. Jiang Y. Farhan M. Lazarovici P. Chen L. Zheng W. (2019). Anti-inflammatory effects of traditional Chinese medicines on preclinical *in vivo* models of brain ischemia-reperfusion-injury: prospects for neuroprotective drug discovery and therapy. Front. Pharmacol. 10, 204 10.3389/fphar.2019.00204 30930774 PMC6423897

[B27] Ren C. Li N. Wang B. Yang Y. Gao J. Li S. (2015). Limb ischemic perconditioning attenuates blood-brain barrier disruption by inhibiting activity of MMP-9 and occludin degradation after focal cerebral ischemia. Aging Dis. 6, 406–417. 10.14336/AD.2015.0812 26618042 PMC4657812

[B28] Ru J. Li P. Wang J. Zhou W. Li B. Huang C. (2014). TCMSP: a database of systems pharmacology for drug discovery from herbal medicines. J. Cheminf. 6, 13 10.1186/1758-2946-6-13 PMC4001360 24735618

[B29] Schmid-Elsaesser R. Zausinger S. Hungerhuber E. Baethmann A. Reulen H. J. (1998). A critical reevaluation of the intraluminal thread model of focal cerebral ischemia: evidence of inadvertent premature reperfusion and subarachnoid hemorrhage in rats by laser-Doppler flowmetry. Stroke 29, 2162–2170. 10.1161/01.str.29.10.2162 9756599

[B30] She Y. Shao L. Zhang Y. Hao Y. Cai Y. Cheng Z. (2019). Neuroprotective effect of glycosides in Buyang Huanwu Decoction on pyroptosis following cerebral ischemia-reperfusion injury in rats. J. Ethnopharmacol. 242, 112051 10.1016/j.jep.2019.112051 31279072

[B31] Wang N. Deng Y. Wei W. Song L. Wang Y. (2012). Serum containing Tongqiaohuoxue decoction suppresses glutamate-induced PC12 cell injury. Neural Regen Res. 7, 1125–1131. 10.3969/j.issn.1673-5374.2012.15.001 25722704 PMC4340028

[B32] Wishart D. S. Feunang Y. D. Guo A. C. Lo E. J. Marcu A. Grant J. R. (2018). DrugBank 5.0: a major update to the DrugBank database for 2018. Nucleic Acids Res. 46, D1074–d1082. 10.1093/nar/gkx1037 29126136 PMC5753335

[B33] Wu S. Wang N. Li J. Wang G. Seto S. W. Chang D. (2019). Ligustilide ameliorates the permeability of the blood-brain barrier model in vitro during oxygen-glucose deprivation injury through HIF/VEGF pathway. J. Cardiovasc. Pharmacol. 73, 316–325. 10.1097/FJC.0000000000000664 30855407

[B34] Wu S. Wang N. He Q. Chang G. Seto S. W. Chang D. (2018). The establishment of the method of cell biochromatograpy and analysis of the active ingredients from TongQiaoHuoXue decoction acting on the neurocytes. Chem. Pharm. Bull. (Tokyo). 66, 983–991. 10.1248/cpb.c18-00455 30270244

[B35] Wu S. P. Li D. Wang N. Hou J. C. Zhao L. (2019). YiQi tongluo granule against cerebral ischemia/reperfusion injury in rats by freezing GluN2B and CaMK II through NMDAR/ERK1/2 signaling. Chem. Pharm. Bull. (Tokyo). 67, 244–252. 10.1248/cpb.c18-00806 30606894

[B36] Xin-Xin P. Ning W. Guang-yun W. Fei-xue Y. Zi-hua X. Rong-feng H. (2015). Effects of naoluo xintong capsules on platelet aggregation and thrombus formation in rats with focal cerebral ischemia/reperfusion. Chin. J. Exp. Trad. Med. Form. 24, 1–4. 10.13422/j.cnki.syfjx.2015220026

[B37] Xu F. Q. Wang N. Liu J. Q. Zhou A. Duan J. A. Ding A. W. (2008). Studies on fingerprints of Tongqiao huoxue decoction by HPLC. Chin. J. Exp. Trad. Med. Form. 14, 1–4.

[B38] Xue R. Fang Z. Zhang M. Yi Z. Wen C. Shi T. (2013). TCMID: traditional Chinese Medicine integrative database for herb molecular mechanism analysis. Nucleic Acids Res. 41, D1089–D1095. 10.1093/nar/gks1100 23203875 PMC3531123

[B39] Yang H. Wang F. Wang Q. L. Zhong Z. D. Li B. Yang K. (2013). Effects of buyang huanwu decoction on expression of TGF-β_1/Smad3 in pulmonary fibrosis rats. Chin. J. Exper. Trad. Med. 19, 240–244. 10.11653/syfj2013240240

[B40] Yang J. L. Fan Y. P. (2009). Clinical effect observation on nervous tinnitus treated with self-made Huoxue Tongqiao Decoction. J Tradit Chin Med. 24, 1476–1477.

[B41] Yin K. J. Hamblin M. Chen Y. E. (2015). Angiogenesis-regulating microRNAs and ischemic stroke. Curr. Vasc. Pharmacol. 13, 352 10.2174/15701611113119990016 26156265 PMC4079753

[B42] You J.-J. Yang C.-H. Yang C.-M. Chen M.-S. (2014). Cyr61 induces the expression of monocyte chemoattractant protein-1 via the integrin ανβ3, FAK, PI3K/Akt, and NF-κB pathways in retinal vascular endothelial cells. Cell. Signal. 26, 133–140. 10.1016/j.cellsig.2013.08.026 24063814

[B43] Zhao Z. Ong L. K. Johnson S. Nilsson M. Walker F. R. (2017). Chronic stress induced disruption of the peri-infarct neurovascular unit following experimentally induced photothrombotic stroke. J. Cerebr. Blood Flow Metabol. 37 (12), 3709–3724. 10.1177/0271678X17696100 PMC5718325 28304184

[B44] Zheng X. W. Shan C. S. Xu Q. Q. Wang Y. Shi Y. H. Wang Y. (2018). Buyang huanwu decoction targets SIRT1/VEGF pathway to promote angiogenesis after cerebral ischemia/reperfusion injury. Front. Neurosci. 12, 911 10.3389/fnins.2018.00911 30564092 PMC6288378

