# Supplementary material for: Network Pharmacology Reveals the Mechanism of Activity of Tongqiao Huoxue Decoction Extract Against Middle Cerebral Artery Occlusion-Induced Cerebral Ischemia-Reperfusion Injury
Source: Front Pharmacol. 2021 Jan 11;11:572624. doi: 10.3389/fphar.2020.572624 (PMC7844429; doi:10.3389/fphar.2020.572624)
Supplement: Supplementary file 1 [file table1.doc]

**Table S1**: The 929 significant genes associated with I/R

| **Gene symbol** | **UNIPROT ID** | **Gene name** |
| --- | --- | --- |
| DRD2  SAE1 UBA2  POLB  AKR1B10  NOX4  AVPR2  AKR1B1  XDH  MAOA  IGF1R  FLT3  CYP19A1  EGFR  F2  CA2  PIM1  ALOX5  AURKB  DRD4  ADORA1  CA7  GLO1  MPO  PIK3R1  ADORA2A  DAPK1  PYGL  CA1  GSK3B  SRC  PTK2  HSD17B2  KDR  MMP13  MMP3  CA3  ALOX15  ABCC1  PLK1  CA6  CDK1  MMP9  CA12  MMP2  PKN1  CA14  CA9  CSNK2A1  ALOX12  MET  CA4  NEK2  CXCR1  CAMK2B  ALK  AKT1  ABCB1  NEK6  PLA2G1B  CA5A  BACE1  CYP1B1  AXL  ABCG2  NUAK1  AKR1C2  AKR1C1  AKR1C3  AKR1C4  CA13  AKR1A1  CDK5R1 CDK5  CCNB3 CDK1 CCNB1 CCNB2  APP  SYK  PARP1  TTR  MMP12  CD38  TNKS2  TNKS  TOP1  ARG1  KDM4E  GRK6  ESR1  ESR2  GPR35  ERBB2  CCND1 CDK4  PDGFRB  FLT4  INSR  CDK2 CCNA1 CCNA2  PLK4  TEK  AURKA  MAP3K8  BRAF  EPHB4  HSPA1A  SQLE  FGR  LYN  HTR2B  BCHE  ADRA2C  ADRA2B  CHRM1  ACHE  SIGMAR1  CYP2D6  PTPN1  DRD5  DRD1  DRD3  F3  PTPRS  NPC1L1  HSD11B1  NR1H3  MAPT  TOP2A  MYLK  PIK3CG  APEX1  HSD17B1  CHRNA4 CHRNB2  HTR7  RORC  HTR6  CDK6  HTR1A  HTR2A  ADRA1D  ADRA1A  ADRA1B  PTPN2  CDC25B  PTPRF  ACP1  HTR5A  MPG  SLC22A12  AR  CDK2  PDE4D  HMGCR  SLC6A4  CYP17A1  PTPRCAP  CD81  PFKFB3  SHBG  SLC6A3  FAAH  TYR  AHR  ESRRA  OPRM1  SREBF2  CYP51A1  PTGS2  CFTR  STAT3  AMY1A  CBR1  TERT  SLC6A2  HTR1D  HTR2C  HTR1B  CYP2C19  ADRB2  ADRB1  ADRA2A  HRH2  ATP1A1  TBXA2R  ADRB3  RORA  IKBKB  NTRK2  PTGES  PTGS1  NOS2  CHRM2  MAOB  GSR  TAS2R31  MMP1  KCNH2  CNR1  CNR2  ADORA3  LCK  PLG  MCL1  LGALS3  LGALS9  NR1I3  ST6GAL1  CES2  CDC25A  PREP  FABP1  PTPN6  HSP90AA1  TH  OPRD1  CALM1  PRKCA  PPARG  FNTA FNTB  SCD  HSD11B2  LTB4R  PRKCH  PTPN11  PPARA  PPARD  FABP4  FABP3  FABP5  NR3C2  SERPINA6  G6PD  PTGER2  NR3C1  GPBAR1  NR1H4  PTGER4  TLR9  ALOX5AP  SRD5A2  PGR  FFAR1  PTGER1  GRIK1  GRIK2  PTGDR2  PTGIR  PTGDR  IL6  GLUL  SLC10A1  SLC10A2  SSTR5  SSTR2  SSTR4  SSTR1  SSTR3  VEGFA  FGF1  FGF2  HPSE  F10  SERPINE1  PTAFR  SELP  P2RX3  PSMB8  EDNRB  CCKBR  LRRK2  CCNA2 CDK2  MAPK1  REN  CCNC CDK8  CDK8  PDE10A  CCR1  ITK  CHEK1  CTSK  CTSS  CTSL  PDE9A  PDE1C  MAPK14  EZH2  NR1I2  PABPC1  FYN  YES1  MDM2  IMPDH2  TACR1  ROCK2  PIK3CB  PIK3CA  FASN  PDE5A  PSEN2 PSENEN NCSTN APH1A PSEN1 APH1B  NTRK1  PDE1A  PDE1B  TNNC1 TNNT2 TNNI3  DHCR7  ATP12A  MAPK3  NR1H2  VDR  UGT2B7  GLRA1  TBXAS1  TNF  IL2  KIF11  CFD  MAPKAPK2  PANK3  RHOA  PIK3CD  OPRK1  CPT1A  CPT2  S1PR3  PPIA  HRH4  GRM5  CAPN1  CTSB  HCRTR2  HCRTR1  HDAC4  MAP3K12  NPY5R  BRD4  FLT1  JAK3  MAPK8  JAK2  FGFR1  BDKRB1  TBK1  CACNA1B  CSF1R  SIRT2  P2RX7  PDGFRA  PDE4B  PDE7A  TGFBR1  CDC25C  DYRK1A  MGAT3  EPHX2  SMO  WNT3A  FKBP1A  SCN9A  MMP8  ELANE  SLC9A1  CMA1  PSMB5  GABRB3 GABRA3 GABRG2  GABRB3 GABRG2 GABRA1  GABRA2 GABRB3 GABRG2  PDE3A  CHRM4  PLAT  MME  TSPO  CHRNB3 CHRNA6 CHRNB2 CHRNA3  ROCK1  IRAK4  HTR1F  HTR1E  CHRM3  CHRNA7  PHLPP2  THRA  HTR3A  THRB  CHRNA3 CHRNB4  SCN4A  CYP1A2  PIM2  CDK9  PRKCQ  LTA4H  NR4A1  XIAP  BIRC2  FDFT1  CHRNB1 CHRNA1 CHRNG CHRND  DPP4  HASPIN  HPGDS  WEE1  PIM3  PARP2  JAK1  PRKCD  PRKCE  PRKD1  PRKX  CDC7  CHRNA4  CDK1 CCNB1  MTNR1B  PAOX  BDKRB2  CHRM5  MC4R  MAP2K1  TTK  ROCK2 ROCK1  HPGD  HDAC6  HDAC1  PKN2  RPS6KA5  FAP  PARP3  RPS6KB1  TYK2  MTNR1A  DPP9  CDC42  MALT1  WDR5  KCNJ1  DAO  HSD17B3  CA5B  SNCA  PORCN  PLAU  F2R  CASR  EPHX1  ATIC  HRH3  ABL1  ACACB  GCGR  CCNE1 CDK2  GRM2  LIPE  NLRP3  AVPR1A  AGTR1  CHEK2  CYP26A1  ICMT  ACE  ECE1  HCAR2  AMPD2  KIT  TRHR  MKNK2  PDPK1  NEK1  TYMS  ERN1  DUSP3  SIRT3  SIRT1  RET  EPHA2  BLK  CSK  EPHB2  BMX  EPHA5  EPHA4  TXK  EPHA6  PTK6  EPHB3  EPHA3  BTK  TYRO3  COQ8B  EPHA1  CTSV  CYP11B1  CYP11B2  HDAC7  ANPEP  PLA2G7  HDAC3  CXCR2  HDAC11  HDAC10  PTK2B  EGLN1  MAP2K2  PRKCG  NCOR1 HDAC3  TACR3  TRPM8  MMP7  STAT6  RAF1  HSP90AB1  ADAM17  TAAR1  ADAM9  ADORA2B  FBP1  ACLY  IL1B  PPP2CA  GSTM1  AMPD3  ATP2A1  PPP1CC  BCL2L1  F2RL1  PCSK7  KCNA3  JUN  CTSA  SELL  SELE  IDO1  PRSS1  CTRC  NAE1  CYP1A1  RPS6KA3  NQO2  NMUR2  ALDH2  DNM1  SCARB1  CAPN1 CAPNS1  CAPN2  HCK  MAPK10  SLC6A9  KCNJ5 KCNJ3  KCNJ6 KCNJ3  GRM1  S1PR1  CTRB1  GABRB3 GABRG2 GABRA5  TGFBR2  PGGT1B FNTA  PDE4A  BCL2  RAD51  OXTR  DBF4 CDC7  CXCR3  SLC2A1  GRK2  HIF1A  SLC2A3  SHH  VCP  MDM4  CRHR1  GABRA3  GABRA2  GABRA5  DYRK1B  PDE4C  PTGFR  PDE11A  PDE3B  TRPV1  KCNK2  CCND3 CCND1  CDK4  CCND2  MAPK9  KCNA5  SLC2A2  NAMPT  CPB1  APLNR  P2RY12  GPR18  CHRNB4 CHRNA2  CHRNA3 CHRNB2  RBBP9  KCNN1  KCNN3  KCNN2  SGK1  CLK1  EZR  HSD17B14  PDK1  ESRRB  LIMK1  CLK3  DYRK2  HDAC5  HDAC9  ALPL  MTOR  SLC37A4  NEU4  PTPN22  CPA1  AMPD1  PYGM  EDNRA  ACE2  ITGAL ICAM1 ITGB2  ITGB1 ITGA4  CCR9  CTNNB1  ITGAL  POLA1  TRPV4  PRKCB  GLI2  PRKCZ  BMP1  CCNE1 CDK3  SLC5A1  MMP14  PNMT  CDK4  TRAP1  HSP90B1  ADAM10  MIF  MMP16  AKT2  EPHA8  EPHA7  PLA2G2A  GABBR1  GABRA2 GABRB2 GABRG2  FABP2  HSF1  CYP2C9  CYP3A4  PRKDC  PI4KB  BRD2  ICAM1  KNG1  TUBB1  FKBP5  VCAM1  F9  ERBB4  NR5A1  BACE2  FPR1  FPR2  SCN10A  BRD9  MKNK1  CDK9 CCNT1  PDE2A  ATM  MAPK11  AGPAT2  CCNE2 CDK2 CCNE1  GCK  CACNA1H  LGMN  RPS6KB2 RPS6KB1  TTL  TACR2  HSPA8  HSPA5  ADK  PPM1A  SLC29A1  LGALS7  SLC28A2  HK2  HK1  GBA  SLC5A11  GAPDH  GLRA2  PLA2G4A  NFKB1  PTP4A3  SLC47A1  SLC18A2  KDM1A  CES1  KLK1  KLK2  SLC5A2  PLA2G5  PLA2G10  STS  PGD  RXRA  DNMT1  STAT1  LPAR6  LPAR5  ENPP2  RBP4  S1PR5  S1PR4  LIMK2  ADCY1  IMPDH1  GABRA1 GABRB2 GABRG2  DHFR  GART  CREBBP  LANCL2  PNP  IL6ST  TRPA1  OPRL1  PER2  RASGRP1  ABCC9  MAP3K20  PRCP  ADA  KCNE1 KCNQ1  PGGT1B  C5AR1  MAP2K4  MAPK13  MAP2K7  MAPK12  MAP3K5  FOLH1  RAC1  GRIA1  SLC1A3  PRF1  BCAT2  TRPC6  PARP10  RPS27  ZAP70  IARS  PTPA  KMT5A  CTSD  GRB2  MLNR  ITGA4  ITGAV ITGB3  ITGA2B ITGB3  ITGB5 ITGAV  ITGAV ITGB6  HDAC2  NCOR2 | P14416  Q9UBE0 Q9UBT2  P06746  O60218  Q9NPH5  P30518  P15121  P47989  P21397  P08069  P36888  P11511  P00533  P00734  P00918  P11309  P09917  Q96GD4  P21917  P30542  P43166  Q04760  P05164  P27986  P29274  P53355  P06737  P00915  P49841  P12931  Q05397  P37059  P35968  P45452  P08254  P07451  P16050  P33527  P53350  P23280  P06493  P14780  O43570  P08253  Q16512  Q9ULX7  Q16790  P68400  P18054  P08581  P22748  P51955  P25024  Q13554  Q9UM73  P31749  P08183  Q9HC98  P04054  P35218  P56817  Q16678  P30530  Q9UNQ0  O60285  P52895  Q04828  P42330  P17516  Q8N1Q1  P14550  Q15078 Q00535  Q8WWL7 P06493 P14635 O95067  P05067  P43405  P09874  P02766  P39900  P28907  Q9H2K2  O95271  P11387  P05089  B2RXH2  P43250  P03372  Q92731  Q9HC97  P04626  P24385 P11802  P09619  P35916  P06213  P24941 P78396 P20248  O00444  Q02763  O14965  P41279  P15056  P54760  P0DMV8  Q14534  P09769  P07948  P41595  P06276  P18825  P18089  P11229  P22303  Q99720  P10635  P18031  P21918  P21728  P35462  P13726  Q13332  Q9UHC9  P28845  Q13133  P10636  P11388  Q15746  P48736  P27695  P14061  P43681 P17787  P34969  P51449  P50406  Q00534  P08908  P28223  P25100  P35348  P35368  P17706  P30305  P10586  P24666  P47898  P29372  Q96S37  P10275  P24941  Q08499  P04035  P31645  P05093  Q14761  P60033  Q16875  P04278  Q01959  O00519  P14679  P35869  P11474  P35372  Q12772  Q16850  P35354  P13569  P40763  P04745  P16152  O14746  P23975  P28221  P28335  P28222  P33261  P07550  P08588  P08913  P25021  P05023  P21731  P13945  P35398  O14920  Q16620  O14684  P23219  P35228  P08172  P27338  P00390  P59538  P03956  Q12809  P21554  P34972  P0DMS8  P06239  P00747  Q07820  P17931  O00182  Q14994  P15907  O00748  P30304  P48147  P07148  P29350  P07900  P07101  P41143  P62158  P17252  P37231  P49354 P49356  O00767  P80365  Q15722  P24723  Q06124  Q07869  Q03181  P15090  P05413  Q01469  P08235  P08185  P11413  P43116  P04150  Q8TDU6  Q96RI1  P35408  Q9NR96  P20292  P31213  P06401  O14842  P34995  P39086  Q13002  Q9Y5Y4  P43119  Q13258  P05231  P15104  Q14973  Q12908  P35346  P30874  P31391  P30872  P32745  P15692  P05230  P09038  Q9Y251  P00742  P05121  P25105  P16109  P56373  P28062  P24530  P32239  Q5S007  P20248 P24941  P28482  P00797  P24863 P49336  P49336  Q9Y233  P32246  Q08881  O14757  P43235  P25774  P07711  O76083  Q14123  Q16539  Q15910  O75469  P11940  P06241  P07947  Q00987  P12268  P25103  O75116  P42338  P42336  P49327  O76074  P49810 Q9NZ42 Q92542 Q96BI3 P49768 Q8WW43  P04629  P54750  Q01064  P63316 P45379 P19429  Q9UBM7  P54707  P27361  P55055  P11473  P16662  P23415  P24557  P01375  P60568  P52732  P00746  P49137  Q9H999  P61586  O00329  P41145  P50416  P23786  Q99500  P62937  Q9H3N8  P41594  P07384  P07858  O43614  O43613  P56524  Q12852  Q15761  O60885  P17948  P52333  P45983  O60674  P11362  P46663  Q9UHD2  Q00975  P07333  Q8IXJ6  Q99572  P16234  Q07343  Q13946  P36897  P30307  Q13627  Q09327  P34913  Q99835  P56704  P62942  Q15858  P22894  P08246  P19634  P23946  P28074  P28472  P18507  P28472  P14867  P47869  P18507  Q14432  P08173  P00750  P08473  P30536  Q05901  P32297  Q13464  Q9NWZ3  P30939  P28566  P20309  P36544  Q6ZVD8  P10827  P46098  P10828  P32297  P35499  P05177  Q9P1W9  P50750  Q04759  P09960  P22736  P98170  Q13490  P37268  P11230  P07510  P27487  Q8TF76  O60760  P30291  Q86V86  Q9UGN5  P23458  Q05655  Q02156  Q15139  P51817  O00311  P43681  P06493  P49286  Q6QHF9  P30411  P08912  P32245  Q02750  P33981  O75116  P15428  Q9UBN7  Q13547  Q16513  O75582  Q12884  Q9Y6F1  P23443  P29597  P48039  Q86TI2  P60953  Q9UDY8  P61964  P48048  P14920  P37058  Q9Y2D0  P37840  Q9H237  P00749  P25116  P41180  P07099  P31939  Q9Y5N1  P00519  O00763  P47871  P24864  Q14416  Q05469  Q96P20  P37288  P30556  O96017  O43174  O60725  P12821  P42892  Q8TDS4  Q01433  P10721  P34981  Q9HBH9  O15530  Q96PY6  P04818  O75460  P51452  Q9NTG7  Q96EB6  P07949  P29317  P51451  P41240  P29323  P51813  P54756  P54764  P42681  Q9UF33  Q13882  P54753  P29320  Q06187  Q06418  Q96D53  P21709  O60911  P15538  P19099  Q8WUI4  P15144  Q13093  O15379  P25025  Q96DB2  Q969S8  Q14289  Q9GZT9  P36507  P05129  O75376  P29371  Q7Z2W7  P09237  P42226  P04049  P08238  P78536  Q96RJ0  Q13443  P29275  P09467  P53396  P01584  P67775  P09488  Q01432  O14983  P36873  Q07817  P55085  Q16549  P22001  P05412  P10619  P14151  P16581  P14902  P07477  Q99895  Q13564  P04798  P51812  P16083  Q9GZQ4  P05091  Q05193  Q8WTV0  P07384  P17655  P08631  P53779  P48067  P48544  P48051  Q13255  P21453  P17538  P28472  P31644  P37173  P53609  P27815  P10415  Q06609  P30559  Q9UBU7  P49682  P11166  P25098  Q16665  P11169  Q15465  P55072  O15151  P34998  P34903  P47869  P31644  Q9Y463  Q08493  P43088  Q9HCR9  Q13370  Q8NER1  O95069  P30281  P11802  P45984  P22460  P11168  P43490  P15086  P35414  Q9H244  Q14330  P30926  P32297  O75884  Q92952  Q9UGI6  Q9H2S1  O00141  P49759  P15311  Q9BPX1  Q15118  O95718  P53667  P49761  Q92630  Q9UQL6  Q9UKV0  P05186  P42345  O43826  Q8WWR8  Q9Y2R2  P15085  P23109  P11217  P25101  Q9BYF1  P20701  P05107  P05556  P51686  P35222  P20701  P09884  Q9HBA0  P05771  P10070  Q05513  P13497  P24864  P13866  P50281  P11086  P11802  Q12931  P14625  O14672  P14174  P51512  P31751  P29322  Q15375  P14555  Q9UBS5  P47869  P18507  P12104  Q00613  P11712  P08684  P78527  Q9UBF8  P25440  P05362  P01042  Q9H4B7  Q13451  P19320  P00740  Q15303  Q13285  Q9Y5Z0  P21462  P25090  Q9Y5Y9  Q9H8M2  Q9BUB5  P50750  O00408  Q13315  Q15759  O15120  O96020  P24864  P35557  O95180  Q99538  Q9UBS0  Q8NG68  P21452  P11142  P11021  P55263  P35813  Q99808  P47929  O43868  P52789  P19367  P04062  Q8WWX8  P04406  P23416  P47712  P19838  O75365  Q96FL8  Q05940  O60341  P23141  P06870  P20151  P31639  P39877  O15496  P08842  P52209  P19793  P26358  P42224  P43657  Q9H1C0  Q13822  P02753  Q9H228  O95977  P53671  Q08828  P20839  P14867  P18507  P00374  P22102  Q92793  Q9NS86  P00491  P40189  O75762  P41146  O15055  O95267  O60706  Q9NYL2  P42785  P00813  P15382  P53609  P21730  P45985  O15264  O14733  P53778  Q99683  Q04609  P63000  P42261  P43003  P14222  O15382  Q9Y210  Q53GL7  P42677  P43403  P41252  Q15257  Q9NQR1  P07339  P62993  O43193  P13612  P06756  P08514  P18084  P06756  Q92769  Q9Y618  P08575  Q92523  P14867  P42574  Q14790  P29466  P19784  P0DJD9  P03951  Q15046  O75907  P30968  P49146  Q9Y6K9  O15111  P33032  Q92934  P23526  P51677  O75874  Q96P09  Q9Y3Q4  O60741  Q13085  P25929 | Dopamine D2 receptor  SUMO-activating enzyme  DNA polymerase beta  Aldo-keto reductase family 1 member B10  NADPH oxidase 4  Vasopressin V2 receptor  Aldose reductase  Xanthine dehydrogenase  Monoamine oxidase A  Insulin-like growth factor I receptor  Tyrosine-protein kinase receptor FLT3  Cytochrome P450 19A1  Epidermal growth factor receptor erbB1  Thrombin  Carbonic anhydrase II  Serine/threonine-protein kinase PIM1  Arachidonate 5-lipoxygenase  Serine/threonine-protein kinase Aurora-B  Dopamine D4 receptor  Adenosine A1 receptor (by homology)  Carbonic anhydrase VII  Glyoxalase I  Myeloperoxidase  PI3-kinase p85-alpha subunit  Adenosine A2a receptor (by homology)  Death-associated protein kinase 1  Liver glycogen phosphorylase  Carbonic anhydrase I  Glycogen synthase kinase-3 beta  Tyrosine-protein kinase SRC  Focal adhesion kinase 1  Estradiol 17-beta-dehydrogenase 2  Vascular endothelial growth factor receptor 2  Matrix metalloproteinase 13  Matrix metalloproteinase 3  Carbonic anhydrase III  Arachidonate 15-lipoxygenase  Multidrug resistance-associated protein 1  Serine/threonine-protein kinase PLK1  Carbonic anhydrase VI  Cyclin-dependent kinase 1  Matrix metalloproteinase 9  Carbonic anhydrase XII  Matrix metalloproteinase 2  Protein kinase N1  Carbonic anhydrase XIV  Carbonic anhydrase IX  Casein kinase II alpha  Arachidonate 12-lipoxygenase  Hepatocyte growth factor receptor  Carbonic anhydrase IV  Serine/threonine-protein kinase NEK2  Interleukin-8 receptor A  CaM kinase II beta  ALK tyrosine kinase receptor  Serine/threonine-protein kinase AKT  P-glycoprotein 1  Serine/threonine-protein kinase NEK6  Phospholipase A2 group 1B  Carbonic anhydrase VA  Beta-secretase 1  Cytochrome P450 1B1  Tyrosine-protein kinase receptor UFO  ATP-binding cassette sub-family G member 2  NUAK family SNF1-like kinase 1  Aldo-keto reductase family 1 member C2 (by homology)  Aldo-keto reductase family 1 member C1 (by homology)  Aldo-keto-reductase family 1 member C3 (by homology)  Aldo-keto reductase family 1 member C4 (by homology)  Carbonic anhydrase XIII (by homology)  Aldehyde reductase (by homology)  Cyclin-dependent kinase 5/CDK5 activator 1  Cyclin-dependent kinase 1/cyclin B  Beta amyloid A4 protein  Tyrosine-protein kinase SYK  Poly [ADP-ribose] polymerase-1  Transthyretin  Matrix metalloproteinase 12  Lymphocyte differentiation antigen CD38  Tankyrase-2  Tankyrase-1  DNA topoisomerase I (by homology)  Arginase-1 (by homology)  Lysine-specific demethylase 4D-like  G protein-coupled receptor kinase 6  Estrogen receptor alpha  Estrogen receptor beta  G-protein coupled receptor 35  Receptor protein-tyrosine kinase erbB-2  Cyclin-dependent kinase 4/cyclin D1  Platelet-derived growth factor receptor beta  Vascular endothelial growth factor receptor 3  Insulin receptor  Cyclin-dependent kinase 2/cyclin A  Serine/threonine-protein kinase PLK4  Tyrosine-protein kinase TIE-2  Serine/threonine-protein kinase Aurora-A  Mitogen-activated protein kinase kinase kinase 8  Serine/threonine-protein kinase B-raf  Ephrin receptor  Heat shock 70 kDa protein 1  Squalene monooxygenase (by homology)  Tyrosine-protein kinase FGR (by homology)  Tyrosine-protein kinase Lyn (by homology)  Serotonin 2b (5-HT2b) receptor  Butyrylcholinesterase  Adrenergic receptor alpha-2  Alpha-2b adrenergic receptor  Muscarinic acetylcholine receptor M1  Acetylcholinesterase  Sigma opioid receptor  Cytochrome P450 2D6  Protein-tyrosine phosphatase 1B  Dopamine D5 receptor  Dopamine D1 receptor  Dopamine D3 receptor  Coagulation factor VII/tissue factor  Receptor-type tyrosine-protein phosphatase S  Niemann-Pick C1-like protein 1  11-beta-hydroxysteroid dehydrogenase 1  LXR-alpha  Microtubule-associated protein tau  DNA topoisomerase II alpha  Myosin light chain kinase, smooth muscle  PI3-kinase p110-gamma subunit  DNA-(apurinic or apyrimidinic site) lyase  Estradiol 17-beta-dehydrogenase 1  Neuronal acetylcholine receptor; alpha4/beta2  Serotonin 7 (5-HT7) receptor  Nuclear receptor ROR-gamma  Serotonin 6 (5-HT6) receptor  Cyclin-dependent kinase 6  Serotonin 1a (5-HT1a) receptor  Serotonin 2a (5-HT2a) receptor  Alpha-1d adrenergic receptor  Alpha-1a adrenergic receptor  Alpha-1b adrenergic receptor  T-cell protein-tyrosine phosphatase  Dual specificity phosphatase Cdc25B  Receptor-type tyrosine-protein phosphatase F (LAR)  Low molecular weight phosphotyrosine protein phosphatase  Serotonin 5a (5-HT5a) receptor  DNA-3-methyladenine glycosylase  Solute carrier family 22 member 12  Androgen Receptor  Cyclin-dependent kinase 2  Phosphodiesterase 4D  HMG-CoA reductase  Serotonin transporter  Cytochrome P450 17A1  Protein tyrosine phosphatase receptor type C-associated protein  CD81 antigen  6-phosphofructo-2-kinase/fructose-2,6-bisphosphatase 3  Testis-specific androgen-binding protein  Dopamine transporter (by homology)  Anandamide amidohydrolase  Tyrosinase  Aryl hydrocarbon receptor  Estrogen-related receptor alpha  Mu opioid receptor  Sterol regulatory element-binding protein 2  Cytochrome P450 51 (by homology)  Cyclooxygenase-2  Cystic fibrosis transmembrane conductance regulator  Signal transducer and activator of transcription 3  AMY1C  Carbonyl reductase [NADPH] 1  Telomerase reverse transcriptase  Norepinephrine transporter  Serotonin 1d (5-HT1d) receptor  Serotonin 2c (5-HT2c) receptor  Serotonin 1b (5-HT1b) receptor  Cytochrome P450 2C19  Adrenergic receptor beta  Beta-1 adrenergic receptor  Alpha-2a adrenergic receptor  Histamine H2 receptor  Sodium/potassium-transporting ATPase alpha-1 chain  Thromboxane A2 receptor  Beta-3 adrenergic receptor  Nuclear receptor ROR-alpha  Inhibitor of nuclear factor kappa B kinase beta subunit  Neurotrophic tyrosine kinase receptor type 2  Prostaglandin E synthase  Cyclooxygenase-1  Nitric oxide synthase, inducible (by homology)  Muscarinic acetylcholine receptor M2  Monoamine oxidase B  Glutathione reductase  Taste receptor type 2 member 31  Matrix metalloproteinase 1  HERG  Cannabinoid receptor 1 (by homology)  Cannabinoid receptor 2  Adenosine A3 receptor  Tyrosine-protein kinase LCK  Plasminogen  Induced myeloid leukemia cell differentiation protein Mcl-1  Galectin-3  Galectin-9  Nuclear receptor subfamily 1 group I member 3 (by homology)  Beta-galactoside alpha-2,6-sialyltransferase 1  Carboxylesterase 2  Dual specificity phosphatase Cdc25A  Prolyl endopeptidase  Fatty acid-binding protein, liver  Protein-tyrosine phosphatase 1C  Heat shock protein HSP 90-alpha  Tyrosine 3-hydroxylase  Delta opioid receptor  Calmodulin  Protein kinase C alpha  Peroxisome proliferator-activated receptor gamma  Protein farnesyltransferase  Acyl-CoA desaturase  11-beta-hydroxysteroid dehydrogenase 2  Leukotriene B4 receptor 1  Protein kinase C eta  Protein-tyrosine phosphatase 2C  Peroxisome proliferator-activated receptor alpha  Peroxisome proliferator-activated receptor delta  Fatty acid binding protein adipocyte  Fatty acid binding protein muscle  Fatty acid binding protein epidermal  Mineralocorticoid receptor  Corticosteroid binding globulin  Glucose-6-phosphate 1-dehydrogenase  Prostanoid EP2 receptor (by homology)  Glucocorticoid receptor  G-protein coupled bile acid receptor 1  Bile acid receptor FXR  Prostanoid EP4 receptor (by homology)  Toll-like receptor (TLR7/TLR9)  5-lipoxygenase activating protein  Steroid 5-alpha-reductase 2  Progesterone receptor  Free fatty acid receptor 1  Prostanoid EP1 receptor (by homology)  Glutamate receptor ionotropic kainate 1  Glutamate receptor ionotropic kainate 2  G protein-coupled receptor 44  Prostanoid IP receptor  Prostanoid DP receptor  Interleukin-6  Glutamine synthetase  Bile acid transporter  Ileal bile acid transporter  Somatostatin receptor 5  Somatostatin receptor 2  Somatostatin receptor 4  Somatostatin receptor 1  Somatostatin receptor 3  Vascular endothelial growth factor A  Acidic fibroblast growth factor  Basic fibroblast growth factor  Heparanase  Thrombin and coagulation factor X  Plasminogen activator inhibitor-1  Platelet activating factor receptor (by homology)  P-selectin  P2X purinoceptor 3  Proteasome subunit beta type-8  Endothelin receptor ET-B  Cholecystokinin B receptor (by homology)  Leucine-rich repeat serine/threonine-protein kinase 2  CDK2/Cyclin A  MAP kinase ERK2  Renin  CDK8/Cyclin C  Cell division protein kinase 8  Phosphodiesterase 10A  C-C chemokine receptor type 1  Tyrosine-protein kinase ITK/TSK  Serine/threonine-protein kinase Chk1  Cathepsin K  Cathepsin S  Cathepsin L  Phosphodiesterase 9A  Phosphodiesterase 1C  MAP kinase p38 alpha  EZH2/SUZ12/EED/RBBP7/RBBP4  Pregnane X receptor  Polyadenylate-binding protein 1  Tyrosine-protein kinase FYN  Tyrosine-protein kinase YES  p53-binding protein Mdm-2  Inosine-5'-monophosphate dehydrogenase 2  Neurokinin 1 receptor  Rho-associated protein kinase 2  PI3-kinase p110-beta subunit  PI3-kinase p110-alpha subunit  Fatty acid synthase  Phosphodiesterase 5A  Gamma-secretase  Nerve growth factor receptor Trk-A  Phosphodiesterase 1A  Phosphodiesterase 1B  Troponin, cardiac muscle  Anti-estrogen binding site (AEBS) (by homology)  Potassium-transporting ATPase alpha chain 2  MAP kinase ERK1  LXR-beta  Vitamin D receptor  UDP-glucuronosyltransferase 2B7  Glycine receptor subunit alpha-1  Thromboxane-A synthase  TNF-alpha  Interleukin-2  Kinesin-like protein 1  Complement factor D  MAP kinase-activated protein kinase 2  Pantothenate kinase 3  Transforming protein RhoA  PI3-kinase p110-delta subunit  Kappa Opioid receptor  Carnitine O-palmitoyltransferase 1, liver isoform  Carnitine palmitoyltransferase 2  Sphingosine 1-phosphate receptor Edg-3  Cyclophilin A  Histamine H4 receptor  Metabotropic glutamate receptor 5  Calpain 1  Cathepsin (B and K)  Orexin receptor 2  Orexin receptor 1  Histone deacetylase 4  Mitogen-activated protein kinase kinase kinase 12  Neuropeptide Y receptor type 5 (by homology)  Bromodomain-containing protein 4  Vascular endothelial growth factor receptor 1  Tyrosine-protein kinase JAK3  c-Jun N-terminal kinase 1  Tyrosine-protein kinase JAK2  Fibroblast growth factor receptor 1  Bradykinin B1 receptor  Serine/threonine-protein kinase TBK1  Voltage-gated N-type calcium channel alpha-1B subunit  Macrophage colony stimulating factor receptor  NAD-dependent deacetylase sirtuin 2  P2X purinoceptor 7  Platelet-derived growth factor receptor alpha  Phosphodiesterase 4B  Phosphodiesterase 7A  TGF-beta receptor type I  Dual specificity phosphatase Cdc25C  Dual-specificity tyrosine-phosphorylation regulated kinase 1A  Beta-1,4-mannosyl-glycoprotein 4-beta-N-acetylglucosaminyltransferase  Epoxide hydratase  Smoothened homolog  Protein Wnt-3a  FK506-binding protein 1A  Sodium channel protein type IX alpha subunit  Matrix metalloproteinase 8  Leukocyte elastase  Sodium/hydrogen exchanger 1  Chymase  Proteasome Macropain subunit MB1  GABA-A receptor; alpha-3/beta-3/gamma-2  GABA-A receptor; alpha-1/beta-3/gamma-2  GABA-A receptor; alpha-2/beta-3/gamma-2  Phosphodiesterase 3  Muscarinic acetylcholine receptor M4  Tissue-type plasminogen activator  Neprilysin  Translocator protein  Neuronal acetylcholine receptor; alpha3/alpha6/beta2/beta3  Rho-associated protein kinase 1  Interleukin-1 receptor-associated kinase 4  Serotonin 1f (5-HT1f) receptor  Serotonin 1e (5-HT1e) receptor  Muscarinic acetylcholine receptor M3  Neuronal acetylcholine receptor protein alpha-7 subunit  PH domain leucine-rich repeat-containing protein phosphatase 2  Thyroid hormone receptor alpha  Serotonin 3a (5-HT3a) receptor  Thyroid hormone receptor beta-1  Neuronal acetylcholine receptor; alpha3/beta4  Sodium channel protein type IV alpha subunit  Cytochrome P450 1A2  Serine/threonine-protein kinase PIM2  Cyclin-dependent kinase 9  Protein kinase C theta  Leukotriene A4 hydrolase  Nuclear receptor subfamily 4 group A member 1  Inhibitor of apoptosis protein 3  Baculoviral IAP repeat-containing protein 2  Squalene synthetase (by homology)  Acetylcholine receptor; alpha1/beta1/delta/gamma  Dipeptidyl peptidase IV  Serine/threonine-protein kinase haspin  Hematopoietic prostaglandin D synthase  Serine/threonine-protein kinase WEE1  Serine/threonine-protein kinase PIM3  Poly [ADP-ribose] polymerase 2  Tyrosine-protein kinase JAK1  Protein kinase C delta  Protein kinase C epsilon  Protein kinase C mu  Serine/threonine-protein kinase PRKX  CDC7/DBF4 (Cell division cycle 7-related protein kinase/Activator of S phase kinase)  Neuronal acetylcholine receptor protein alpha-4 subunit  Cyclin-dependent kinase 1/cyclin B1  Melatonin receptor 1B  Polyamine oxidase  Bradykinin B2 receptor  Muscarinic acetylcholine receptor M5 (by homology)  Melanocortin receptor 4  Dual specificity mitogen-activated protein kinase kinase 1  Dual specificity protein kinase TTK  Rho-associated protein kinase  15-hydroxyprostaglandin dehydrogenase [NAD+]  Histone deacetylase 6  Histone deacetylase 1  Protein kinase N2  Ribosomal protein S6 kinase alpha 5  Fibroblast activation protein alpha  Poly [ADP-ribose] polymerase 3  Ribosomal protein S6 kinase 1  Tyrosine-protein kinase TYK2  Melatonin receptor 1A  Dipeptidyl peptidase IX  Cell division control protein 42 homolog  Mucosa-associated lymphoid tissue lymphoma translocation protein 1  WD repeat-containing protein 5  ATP-sensitive inward rectifier potassium channel 1  D-amino-acid oxidase  Estradiol 17-beta-dehydrogenase 3  Carbonic anhydrase VB  Alpha-synuclein  Probable protein-cysteine N-palmitoyltransferase porcupine  Urokinase-type plasminogen activator  Proteinase-activated receptor 1  Calcium sensing receptor  Epoxide hydrolase 1  AICAR transformylase  Histamine H3 receptor  Tyrosine-protein kinase ABL  Acetyl-CoA carboxylase 2  Glucagon receptor  Cyclin-dependent kinase 2/cyclin E1  Metabotropic glutamate receptor 2  Hormone sensitive lipase  NACHT, LRR and PYD domains-containing protein 3  Vasopressin V1a receptor  Type-1 angiotensin II receptor (by homology)  Serine/threonine-protein kinase Chk2  Cytochrome P450 26A1  Isoprenylcysteine carboxyl methyltransferase  Angiotensin-converting enzyme  Endothelin-converting enzyme 1  Hydroxycarboxylic acid receptor 2  AMP deaminase 2  Stem cell growth factor receptor  Thyrotropin-releasing hormone receptor (by homology)  MAP kinase signal-integrating kinase 2  3-phosphoinositide dependent protein kinase-1  Serine/threonine-protein kinase Nek1  Thymidylate synthase  Serine/threonine-protein kinase/endoribonuclease IRE1  Dual specificity protein phosphatase 3  NAD-dependent deacetylase sirtuin 3  NAD-dependent deacetylase sirtuin 1  Kinesin-1 heavy chain/ Tyrosine-protein kinase receptor RET  Ephrin type-A receptor 2  Tyrosine-protein kinase BLK  Tyrosine-protein kinase CSK  Ephrin type-B receptor 2  Tyrosine-protein kinase BMX  Ephrin type-A receptor 5  Ephrin type-A receptor 4  Tyrosine-protein kinase TXK  Ephrin type-A receptor 6  Tyrosine-protein kinase BRK  Ephrin type-B receptor 3  Ephrin type-A receptor 3  Tyrosine-protein kinase BTK  Tyrosine-protein kinase receptor TYRO3  Uncharacterized aarF domain-containing protein kinase 4  Ephrin type-A receptor 1  Cathepsin (V and K)  Cytochrome P450 11B1  Cytochrome P450 11B2  Histone deacetylase 7  Aminopeptidase N  LDL-associated phospholipase A2  Histone deacetylase 3  Interleukin-8 receptor B  Histone deacetylase 11  Histone deacetylase 10  Protein tyrosine kinase 2 beta  Egl nine homolog 1  Dual specificity mitogen-activated protein kinase kinase 2  Protein kinase C gamma (by homology)  Histone deacetylase 3/NCoR1  Neurokinin 3 receptor  Transient receptor potential cation channel subfamily M member 8 (by homology)  Matrix metalloproteinase 7  Signal transducer and activator of transcription 6  Serine/threonine-protein kinase RAF  Heat shock protein HSP 90-beta  ADAM17  Trace amine-associated receptor 1 (by homology)  ADAM9  Adenosine A2b receptor  Fructose-1,6-bisphosphatase  ATP-citrate synthase  Interleukin-1 beta  Serine/threonine protein phosphatase 2A, catalytic subunit, alpha isoform  Glutathione S-transferase Mu 1  AMP deaminase 3  Sarcoplasmic/endoplasmic reticulum calcium ATPase 1  Serine/threonine protein phosphatase PP1-gamma catalytic subunit  Apoptosis regulator Bcl-X  Proteinase-activated receptor 2  Subtilisin/kexin type 7  Voltage-gated potassium channel subunit Kv1.3  Proto-oncogene c-JUN  Lysosomal protective protein  Leukocyte adhesion molecule-1  Selectin E  Indoleamine 2,3-dioxygenase  Trypsin I  Chymotrypsin C  NEDD8-activating enzyme E1 regulatory subunit  Cytochrome P450 1A1  Ribosomal protein S6 kinase alpha 3  Quinone reductase 2  Neuromedin-U receptor 2  Aldehyde dehydrogenase  Dynamin-1  Scavenger receptor class B member 1 (by homology)  Calpain 1  Calpain 2  Tyrosine-protein kinase HCK  c-Jun N-terminal kinase 3  Glycine transporter 1  Kir3.1/Kir3.4  Kir3.1/Kir3.2  Metabotropic glutamate receptor 1  Sphingosine 1-phosphate receptor Edg-1  Beta-chymotrypsin  GABA-A receptor; alpha-5/beta-3/gamma-2  TGF-beta receptor type II  Geranylgeranyl transferase type I  Phosphodiesterase 4A  Apoptosis regulator Bcl-2  DNA repair protein RAD51 homolog 1  Oxytocin receptor  CDC7/DBF4 (Cell division cycle 7-related protein kinase/Activator of S phase kinase)  C-X-C chemokine receptor type 3  Glucose transporter  G-protein coupled receptor kinase 2  Hypoxia-inducible factor 1 alpha  Solute carrier family 2, facilitated glucose transporter member 3  Sonic hedgehog protein  Transitional endoplasmic reticulum ATPase  Protein Mdm4  Corticotropin releasing factor receptor 1  GABA receptor alpha-3 subunit  GABA receptor alpha-2 subunit  GABA receptor alpha-5 subunit  Dual specificity tyrosine-phosphorylation-regulated kinase 1B  Phosphodiesterase 4C  Prostanoid FP receptor  Phosphodiesterase 11A  Phosphodiesterase 3B  Vanilloid receptor  Potassium channel subfamily K member 2 (by homology)  Cyclin-dependent kinase 4/cyclin D  c-Jun N-terminal kinase 2  Voltage-gated potassium channel subunit Kv1.5  Solute carrier family 2, facilitated glucose transporter member 2  Nicotinamide phosphoribosyltransferase  Carboxypeptidase B  Apelin receptor  Purinergic receptor P2Y12  N-arachidonyl glycine receptor  Neuronal acetylcholine receptor; alpha2/beta4  Neuronal acetylcholine receptor; alpha3/beta2  Putative hydrolase RBBP9  Small conductance calcium-activated potassium channel protein 1  Small conductance calcium-activated potassium channel protein 3  Small conductance calcium-activated potassium channel protein 2  Serine/threonine-protein kinase Sgk1  Dual specificty protein kinase CLK1  Ezrin  17-beta-hydroxysteroid dehydrogenase 14  Pyruvate dehydrogenase kinase isoform 1  Estrogen-related receptor beta  LIM domain kinase 1  Dual specificity protein kinase CLK3 (by homology)  Dual-specificity tyrosine-phosphorylation regulated kinase 2  Histone deacetylase 5  Histone deacetylase 9  Alkaline phosphatase, tissue-nonspecific isozyme  Serine/threonine-protein kinase mTOR  Glucose-6-phosphate translocase  Sialidase 4  Hematopoietic cell protein-tyrosine phosphatase 70Z-PEP  Carboxypeptidase A1  AMP deaminase 1  Muscle glycogen phosphorylase  Endothelin receptor ET-A (by homology)  Angiotensin-converting enzyme 2  Intercellular adhesion molecule (ICAM-1), Integrin alpha-L/beta-2  Integrin alpha-4/beta-1  C-C chemokine receptor type 9  Axin1/beta-catenin  Leukocyte adhesion glycoprotein LFA-1 alpha  DNA polymerase alpha subunit  Transient receptor potential cation channel subfamily V member 4 (by homology)  Protein kinase C beta  Zinc finger protein GLI2  Protein kinase C (PKC)  Bone morphogenetic protein 1  CDK3/Cyclin E  Sodium/glucose cotransporter 1  Matrix metalloproteinase 14  Phenylethanolamine N-methyltransferase  Cyclin-dependent kinase 4  Heat shock protein 75 kDa, mitochondrial  Endoplasmin  ADAM10  Macrophage migration inhibitory factor  Matrix metalloproteinase 16  Serine/threonine-protein kinase AKT2  Ephrin type-A receptor 8  Ephrin type-A receptor 7  Phospholipase A2 group IIA  GABA-B receptor (by homology)  GABA A receptor alpha-2/beta-2/gamma-2  Fatty acid binding protein intestinal  Heat shock factor protein 1  Cytochrome P450 2C9  Cytochrome P450 3A4  DNA-dependent protein kinase  PI4-kinase beta subunit  Bromodomain-containing protein 2  Intercellular adhesion molecule-1  Kininogen-1  Tubulin beta-1 chain  Peptidyl-prolyl cis-trans isomerase FKBP5  Vascular cell adhesion protein 1  Coagulation factor IX  Receptor protein-tyrosine kinase erbB-4  Steroidogenic factor 1  Beta secretase 2  Formyl peptide receptor 1  Lipoxin A4 receptor  Sodium channel protein type X alpha subunit (by homology)  Bromodomain-containing protein 9  MAP kinase-interacting serine/threonine-protein kinase MNK1  CDK9/cyclin T1  Phosphodiesterase 2A  Serine-protein kinase ATM  MAP kinase p38 beta (by homology)  1-acylglycerol-3-phosphate O-acyltransferase beta  Cyclin-dependent kinase 2/cyclin E  Hexokinase type IV  Voltage-gated T-type calcium channel alpha-1H subunit  Legumain (by homology)  Ribosomal protein S6 kinase (P70S6K)  Tubulin--tyrosine ligase  Neurokinin 2 receptor  Heat shock cognate 71 kDa protein  78 kDa glucose-regulated protein  Adenosine kinase  Protein phosphatase 2C alpha  Equilibrative nucleoside transporter 1  P47929  Sodium/nucleoside cotransporter 2  Hexokinase type II  Hexokinase type I  Beta-glucocerebrosidase  Sodium/myo-inositol cotransporter 2  Glyceraldehyde-3-phosphate dehydrogenase liver  Glycine receptor subunit alpha-2  Cytosolic phospholipase A2  Nuclear factor NF-kappa-B p105 subunit  Protein-tyrosine phosphatase 4A3  Multidrug and toxin extrusion protein 1  Synaptic vesicular amine transporter (by homology)  Lysine-specific histone demethylase 1  Acyl coenzyme A:cholesterol acyltransferase  Kallikrein 1  Kallikrein 2  Sodium/glucose cotransporter 2  Phospholipase A2 group V  Group X secretory phospholipase A2  Steryl-sulfatase  6-phosphogluconate dehydrogenase  Retinoid X receptor alpha  DNA (cytosine-5)-methyltransferase 1  Signal transducer and activator of transcription 1-alpha/beta  Lysophosphatidic acid receptor 6  Lysophosphatidic acid receptor 5  Autotaxin  Plasma retinol-binding protein  Sphingosine 1-phosphate receptor Edg-8  Sphingosine 1-phosphate receptor Edg-6  LIM domain kinase 2  Brain adenylate cyclase 1  Inosine-5'-monophosphate dehydrogenase 1  GABA-A receptor; alpha-1/beta-2/gamma-2  Dihydrofolate reductase  GAR transformylase (by homology)  CREB-binding protein/p53  LanC-like protein 2  Purine nucleoside phosphorylase (by homology)  Interleukin-6 receptor subunit beta  Transient receptor potential cation channel subfamily A member 1  Nociceptin receptor  Period circadian protein homolog 2  RAS guanyl-releasing protein 1 (by homology)  Sulfonylurea receptor 2  Mixed lineage kinase 7  Lysosomal Pro-X carboxypeptidase  Adenosine deaminase  Voltage-gated potassium channel, IKs; KCNQ1(Kv7.1)/KCNE1(MinK)  Geranylgeranyl transferase type I beta subunit  C5a anaphylatoxin chemotactic receptor  Dual specificity mitogen-activated protein kinase kinase 4  MAP kinase p38 delta  Dual specificity mitogen-activated protein kinase kinase 7  MAP kinase p38 gamma  Mitogen-activated protein kinase kinase kinase 5  Glutamate carboxypeptidase II  Ras-related C3 botulinum toxin substrate 1  Glutamate receptor ionotropic, AMPA 1  Excitatory amino acid transporter 1  Perforin-1  Branched-chain-amino-acid aminotransferase, mitochondrial  Short transient receptor potential channel 6  Poly [ADP-ribose] polymerase 10  40S ribosomal protein S27  Tyrosine-protein kinase ZAP-70  Isoleucyl-tRNA synthetase  Protein phosphatase 2A regulatory subunit B'  N-lysine methyltransferase SETD8  Cathepsin D  Growth factor receptor-bound protein 2  Motilin receptor  Integrin alpha-4  Integrin alpha-V/beta-3  Integrin alpha-IIb/beta-3  Integrin alpha-V/beta-5  Integrin alpha-V/beta-6  Histone deacetylase 2  Histone deacetylase 3/Nuclear receptor corepressor 2 (HDAC3/NCoR2)  Leukocyte common antigen  Carnitine O-palmitoyltransferase 1, muscle isoform  GABA receptor alpha-1 subunit  Caspase-3  Caspase-8  Caspase-1  Casein kinase II alpha (prime)  Pepsin A  Coagulation factor XI  Lysyl-tRNA synthetase  Diacylglycerol O-acyltransferase 1  Gonadotropin-releasing hormone receptor  Neuropeptide Y receptor type 2  Inhibitor of NF-kappa-B kinase (IKK)  Melanocortin receptor 5  Bcl2-antagonist of cell death (BAD)  Adenosylhomocysteinase  C-C chemokine receptor type 3  Isocitrate dehydrogenase [NADP] cytoplasmic  Baculoviral IAP repeat-containing protein 8  Potassium/sodium hyperpolarization-activated cyclic nucleotide-gated channel 4  Potassium/sodium hyperpolarization-activated cyclic nucleotide-gated channel 1  Acetyl-CoA carboxylase 1  Neuropeptide Y receptor type 1  C-X-C chemokine receptor type 4  C-C chemokine receptor type 4  Melanin-concentrating hormone receptor 1  G protein-coupled receptor kinase 7  Homeodomain-interacting protein kinase 4  Serine/threonine-protein kinase OSR1  STE20/SPS1-related proline-alanine-rich protein kinase  Mitogen-activated protein kinase kinase kinase 13  Serine/threonine-protein kinase ICK  Mitogen-activated protein kinase kinase kinase 15  Microtubule-associated serine/threonine-protein kinase 1  Serine/threonine-protein kinase SBK1  Serine/threonine-protein kinase PRP4 homolog  Dual specificity mitogen-activated protein kinase kinase 3  AMP-activated protein kinase, alpha-2 subunit  Protein phosphatase 2C beta  Serine/threonine protein phosphatase 2A, 56 kDa regulatory subunit, alpha isoform  Transforming growth factor beta-1  Serine/threonine-protein kinase EEF2K |
|  |  |  |
