# Supplementary material for: Network Pharmacology Reveals the Mechanism of Activity of Tongqiao Huoxue Decoction Extract Against Middle Cerebral Artery Occlusion-Induced Cerebral Ischemia-Reperfusion Injury
Source: Front Pharmacol. 2021 Jan 11;11:572624. doi: 10.3389/fphar.2020.572624 (PMC7844429; doi:10.3389/fphar.2020.572624)
Supplement: Supplementary file 2 [file table2.doc]

**Table S2**: The detailed information about compounds of Tongqiao Huoxue Decoction

| **Serial number** | **Ingredients** | **OB** | **DL** |
| --- | --- | --- | --- |
| MOL000359 | sitosterol | 36.91 | 0.75 |
| MOL000433 | FA | 68.96 | 0.71 |
| MOL001494 | Mandenol | 42 | 0.19 |
| MOL002135 | Myricanone | 40.6 | 0.51 |
| MOL002140 | Perlolyrine | 65.95 | 0.27 |
| MOL002151 | senkyunone | 47.66 | 0.24 |
| MOL002157 | wallichilide | 42.31 | 0.71 |
| MOL001002  MOL001918  MOL001921  MOL001924  MOL001925  MOL002714  MOL002776  MOL000358  MOL004355  MOL000449  MOL000492  MOL006990  MOL006992  MOL006994  MOL001323  MOL001328  MOL001329  MOL001339  MOL001340  MOL001342  MOL001343  MOL001344  MOL001348  MOL001349  MOL001350  MOL001351  MOL001352  MOL001353  MOL001355  MOL001358  MOL001360  MOL001361  MOL001368  MOL001371  MOL000296  MOL000493  MOL001771  MOL002680  MOL002694  MOL002695  MOL002698  MOL002706  MOL002707  MOL002710  MOL002712  MOL002717  MOL002719  MOL002721  MOL002757  MOL002773  MOL000422  MOL000006  MOL000953  MOL000098  MOL002464  MOL002501  MOL002514  MOL012921  MOL012940  MOL012946  MOL012961  MOL012976  MOL012980  MOL012981  MOL012986  MOL012989  MOL012992  MOL001454  MOL001522  MOL000211  MOL003410  MOL004350  MOL005360  MOL000627  MOL007213  MOL000783  MOL000787  MOL008034  MOL008647  MOL000096  MOL013357  MOL011319  MOL011628  MOL001749  MOL002122 CID:6443665  CID: 10947 | ellagic acid  paeoniflorgenone  Lactiflorin  paeoniflorin  paeoniflorin_qt  baicalein  Baicalin  beta-sitosterol  Spinasterol  Stigmasterol  (+)-catechin  (1S,2S,4R)-trans-2-hydroxy-1,8-cineole-B-D-glucopyranoside  (2R,3R)-4-methoxyl-distylin  1-o-beta-d-glucopyranosyl-8-o-benzoylpaeonisuffrone_qt  Sitosterol alpha1  2,3-didehydro GA70  2,3-didehydro GA77  GA119  GA120  GA121-isolactone  GA122  GA122-isolactone  gibberellin 17  4a-formyl-7alpha-hydroxy-1-methyl-8-methylidene-4aalpha,4bbeta-gibbane-1alpha,10beta-dicar  GA30  Gibberellin A44  GA54  GA60  GA63  gibberellin 7  GA77  GA87  3-O-p-coumaroylquinic acid  Populoside_qt  hederagenin  campesterol  poriferast-5-en-3beta-ol  Flavoxanthin  4-[(E)-4-(3,5-dimethoxy-4-oxo-1-cyclohexa-2,5-dienylidene)but-2-enylidene]-2,6-dimethoxycycl  lignan  lupeol-palmitate  Phytoene  phytofluene  Pyrethrin II  6-Hydroxykaempferol  qt_carthamone  6-Hydroxynaringenin  quercetagetin  7,8-dimethyl-1H-pyrimido[5,6-g]quinoxaline-2,4-dione  beta-carotene  kaempferol  luteolin  CLR  Quercetin  1-Monolinolein  [(1S)-3-[(E)-but-2-enyl]-2-methyl-4-oxo-1-cyclopent-2-enyl] (1R,3R)-3-[(E)-3-methoxy-2-methyl-3-oxoprop-1-enyl]-2,2-dimethylcyclopropane-1-carboxylate  stepharine  Spiradine A  zizyphus saponin I_qt  jujuboside A_qt  coumestrol  Daechuine S6  Daechuine S7  Jujubasaponin V_qt  Jujuboside C_qt  Mauritine D  berberine  (S)-Coclaurine  Mairin  Ziziphin_qt  Ruvoside_qt  malkangunin  Stepholidine  Nuciferin  Protoporphyrin  Fumarine  21302-79-4  Moupinamide  (-)-catechin  (3S,6R,8S,9S,10R,13R,14S,17R)-17-[(1R,4R)-4-ethyl-1,5-dimethylhexyl]-10,13-dimethyl-2,3,6,7,8,9,11,12,14,15,16,17-dodecahydro-1H-cyclopenta[a]phenanthrene-3,6-diol  Truflex OBP  Kaur-16-ene  ZINC03860434  (Z)-Ligustilide  Hydroxysafflor Yellow A  Muscone | 43.06  87.59  49.12  53.87  68.18  33.52  40.12  36.91  42.98  43.83  54.83  30.25  59.98  36.01  43.28  63.29  88.08  76.36  84.85  72.7  64.79  88.11  94.64  88.6  61.72  101.61  64.21  93.17  65.54  73.8  87.89  68.85  37.63  108.89  36.91  37.58  36.91  60.41  48.47  43.32  33.98  39.56  43.18  48.36  62.13  51.03  33.23  45.01  45.75  37.18  41.88  36.16  37.87  46.43  37.18  62.52  62.86  31.55  113.52  32.69  36.67  32.49  46.48  44.82  36.99  40.26  89.13  36.86  42.35  55.38  66.95  36.12  57.71  33.11  34.43  30.86  59.26  73.52  86.71  49.68  34.37  43.74  33.28  43.59  53.72 | 0.43  0.37  0.8  0.79  0.4  0.21  0.75  0.75  0.76  0.76  0.24  0.27  0.3  0.3  0.78  0.5  0.53  0.49  0.45  0.54  0.5  0.54  0.49  0.46  0.54  0.54  0.53  0.53  0.54  0.5  0.53  0.57  0.29  0.2  0.75  0.71  0.75  0.56  0.36  0.65  0.32  0.5  0.5  0.35  0.27  0.2  0.24  0.31  0.19  0.58  0.24  0.25  0.68  0.28  0.3  0.31  0.3  0.33  0.61  0.62  0.62  0.34  0.79  0.83  0.63  0.62  0.45  0.78  0.24  0.78  0.62  0.76  0.63  0.54  0.4  0.56  0.83  0.77  0.26  0.24  0.78  0.24  0.27  0.35  0.07 |
