# Supplementary material for: Network Pharmacology Reveals the Mechanism of Activity of Tongqiao Huoxue Decoction Extract Against Middle Cerebral Artery Occlusion-Induced Cerebral Ischemia-Reperfusion Injury
Source: Front Pharmacol. 2021 Jan 11;11:572624. doi: 10.3389/fphar.2020.572624 (PMC7844429; doi:10.3389/fphar.2020.572624)
Supplement: Supplementary file 3 [file table3.docx]

**Table. S 3 The methodology results （λ254nm，n=6）**

| **Number** | **Relative retention time** | | | **Relative reserved peak area** | | | |
| --- | --- | --- | --- | --- | --- | --- | --- |
|  | **Precision stability Repeatability** | | | **Precision stability Repeatability** | | | |
| 1 | 0.298±0.0191 | 0.293±0.0025 | 0.293±0.0053 | 2.376±0.0115 | | 2.201±0.0103 | 2.292±0.0231 |
| 2 | 0.335±0.0107 | 0.340±0.0013 | 0.340±0.0021 | 1.145±0.009 | | 1.159±0.0035 | 1.162±0.0032 |
| 3 | 0.501±0.0069 | 0.507±0.0112 | 0.505±0.0101 | 0.814±0.0219 | | 0.846±0.0307 | 0.855±0.009 |
| 4 | 0.615±0.0174 | 0.621±0.0074 | 0.619±0.0038 | 0.763±0.0215 | | 0.792±0.0133 | 0.801±0.0262 |
| 5 | 0.734±0.0212 | 0.741±0.0019 | 0.740±0.0033 | 5.269±0.0149 | | 5.263±0.0193 | 5.263±0.0272 |
| 6 | 0.852±0.0214 | 0.855±0.0015 | 0.861±0.0102 | 2.045±0.0311 | | 2.047±0.0194 | 2.049±0.0258 |
| 8 | 1.325±0.0102 | 1.329±0.0003 | 1.328±0.0015 | 0.876±0.0175 | | 0.797±0.0141 | 0.813±0.0338 |
| 9 | 1.432±0.0035 | 1.438±0.0016 | 1.436±0.0103 | 1.101±0.0037 | | 1.091±0.0042 | 1.087±0.0102 |
| 10 | 1.571±0.0011 | 1.575±0.0103 | 1.574±0.0082 | 2.241±0.0065 | | 2.272±0.0130 | 2.302±0.0229 |
| 11 | 1.683±0.0113 | 1.686±0.0105 | 1.685±0.0077 | 0.993±0.0076 | | 0.898±0.0007 | 0.931±0.0126 |
| **7** | **Reference peak (paeoniflorin)** | | | | **Reference peak (paeoniflorin)** | | |
