# Supplementary figures and images for: Network Pharmacology Reveals the Mechanism of Activity of Tongqiao Huoxue Decoction Extract Against Middle Cerebral Artery Occlusion-Induced Cerebral Ischemia-Reperfusion Injury
Source: Front Pharmacol. 2021 Jan 11;11:572624. doi: 10.3389/fphar.2020.572624 (PMC7844429; doi:10.3389/fphar.2020.572624)

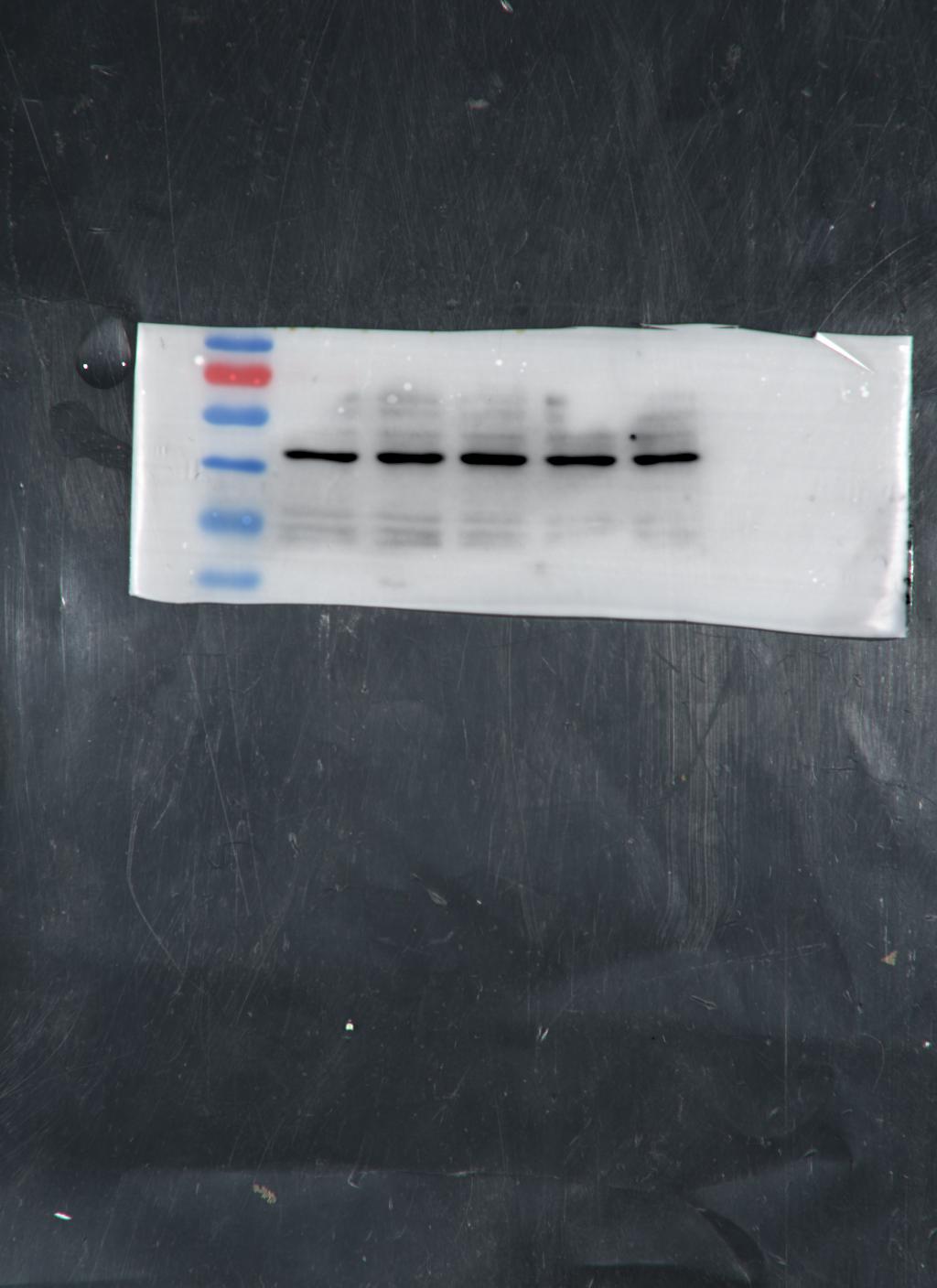

Supplement: Supplementary file 4 [file datasheet1.zip › (2020.8.14) WB image/original image files of the western blots/actin/2017.05.04_05.10.00_Ch+Marker.jpg]

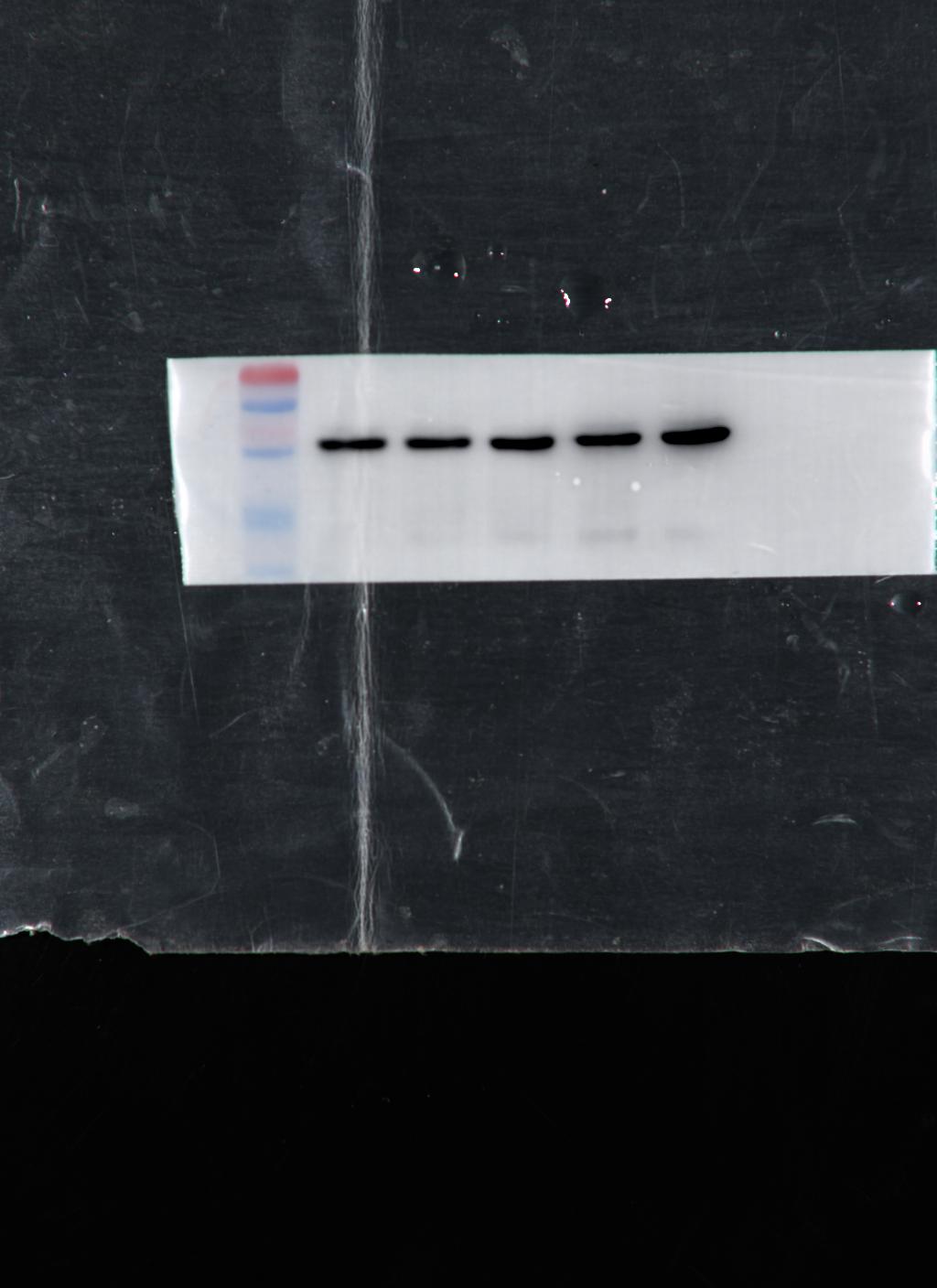

Supplement: Supplementary file 4 [file datasheet1.zip › (2020.8.14) WB image/original image files of the western blots/actin/actin-0 2017.12.01_13.03.06_Ch+Marker.jpg]

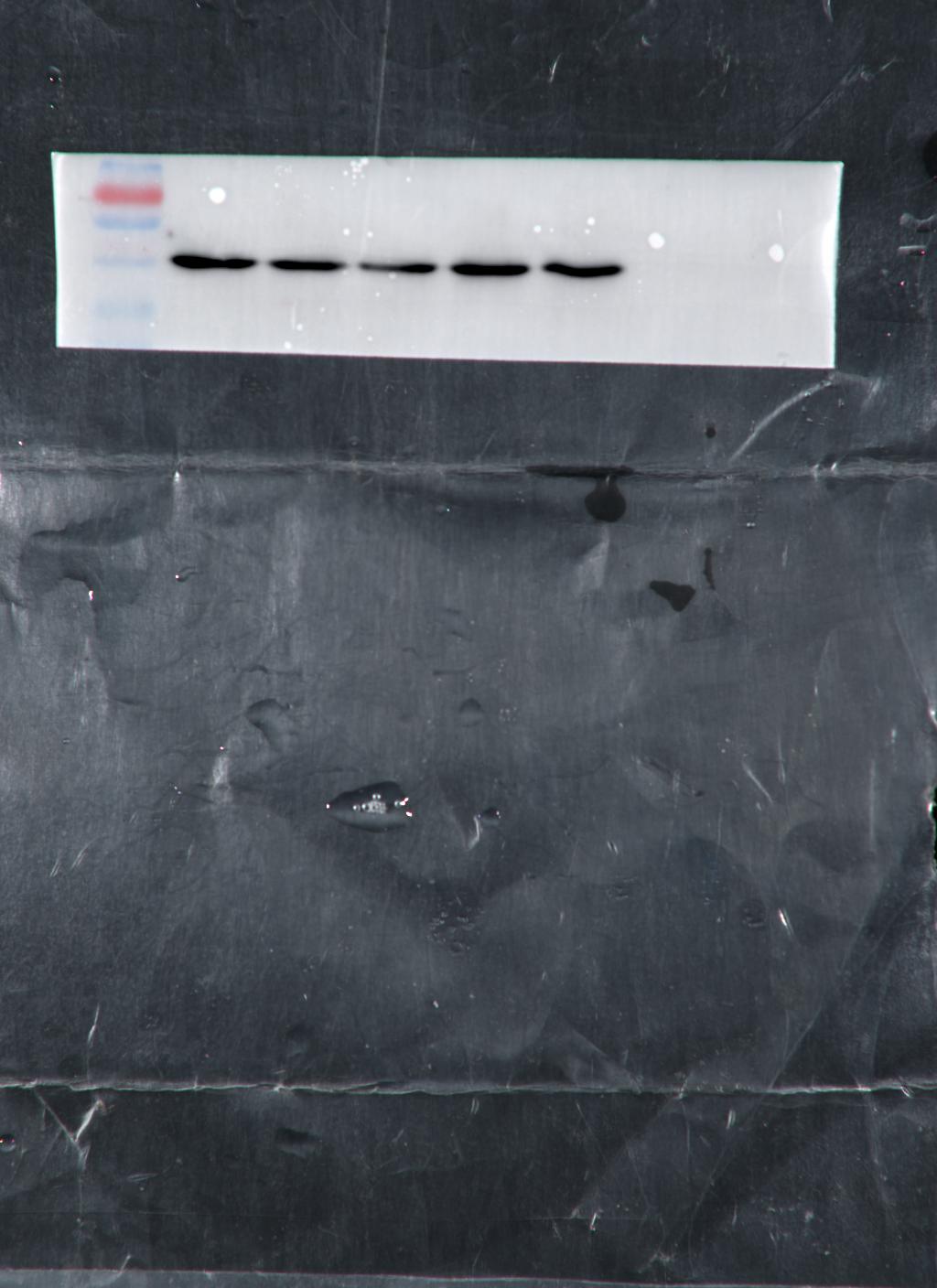

Supplement: Supplementary file 4 [file datasheet1.zip › (2020.8.14) WB image/original image files of the western blots/actin/actin-1 2017.12.03_12.28.29_Ch+Marker.jpg]

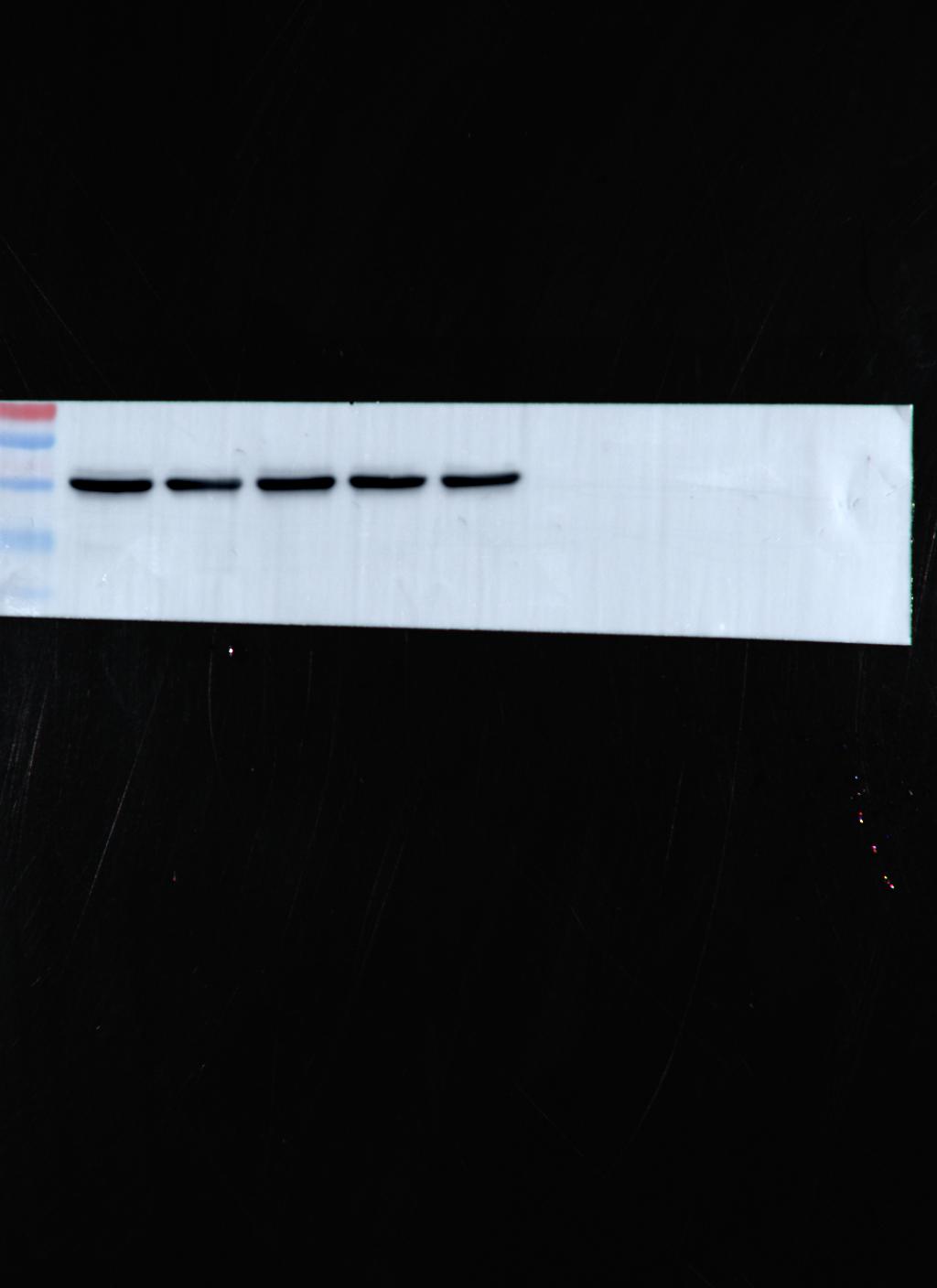

Supplement: Supplementary file 4 [file datasheet1.zip › (2020.8.14) WB image/original image files of the western blots/actin/actin-2 2017.12.04_22.30.32_Ch+Marker.jpg]

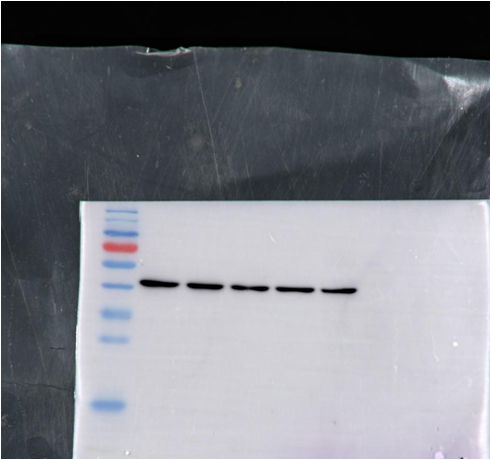

Supplement: Supplementary file 4 [file datasheet1.zip › (2020.8.14) WB image/original image files of the western blots/actin/actin-3 2017.12.04_22.30.32_Ch+Marker.tif]

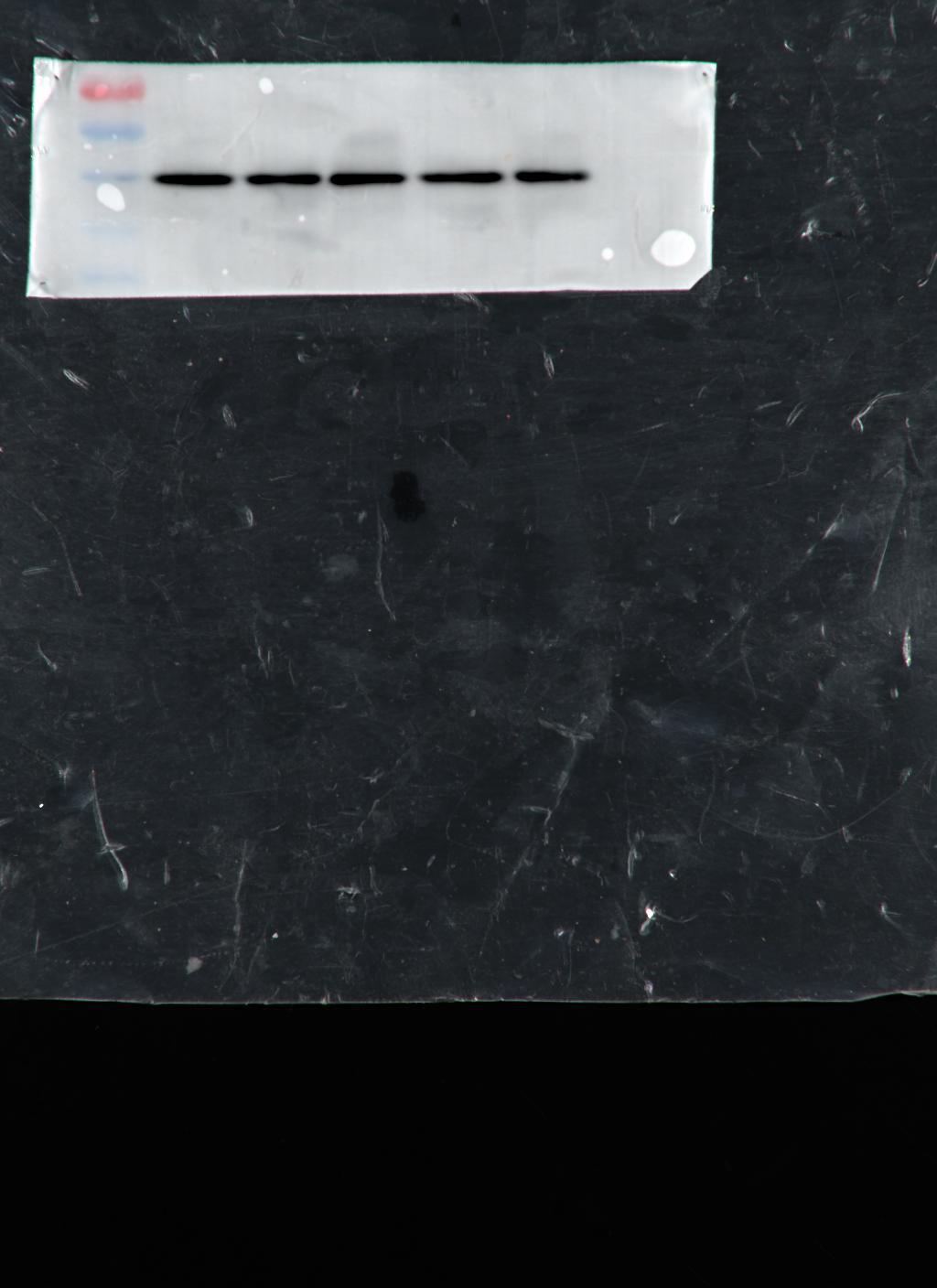

Supplement: Supplementary file 4 [file datasheet1.zip › (2020.8.14) WB image/original image files of the western blots/actin/actin-mechanism 2017.12.04_22.30.32_Ch+Marker.jpg]

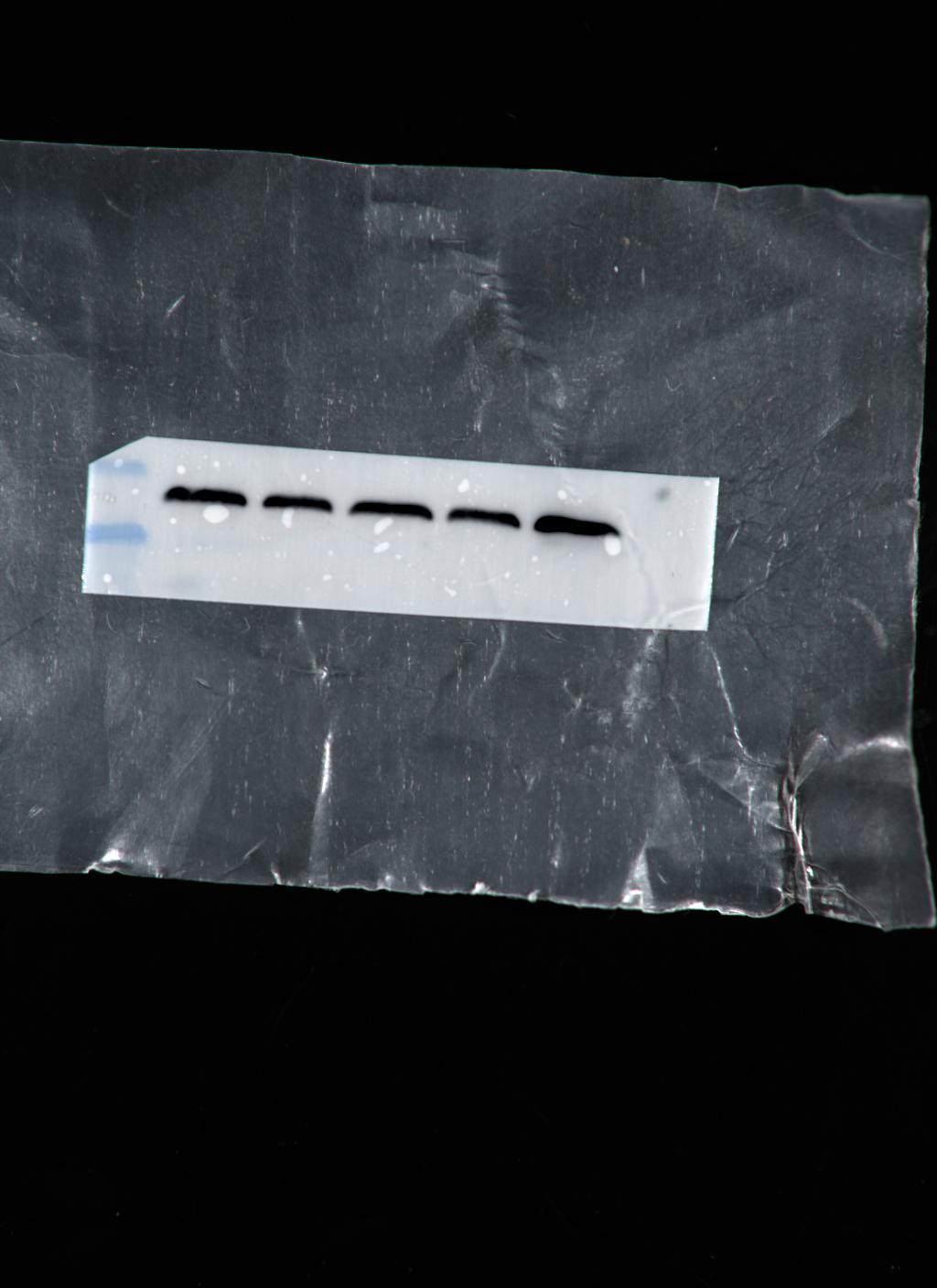

Supplement: Supplementary file 4 [file datasheet1.zip › (2020.8.14) WB image/original image files of the western blots/actin/wsp actin 0810 2018.08.10_15.22.41_Ch+Marker.jpg]

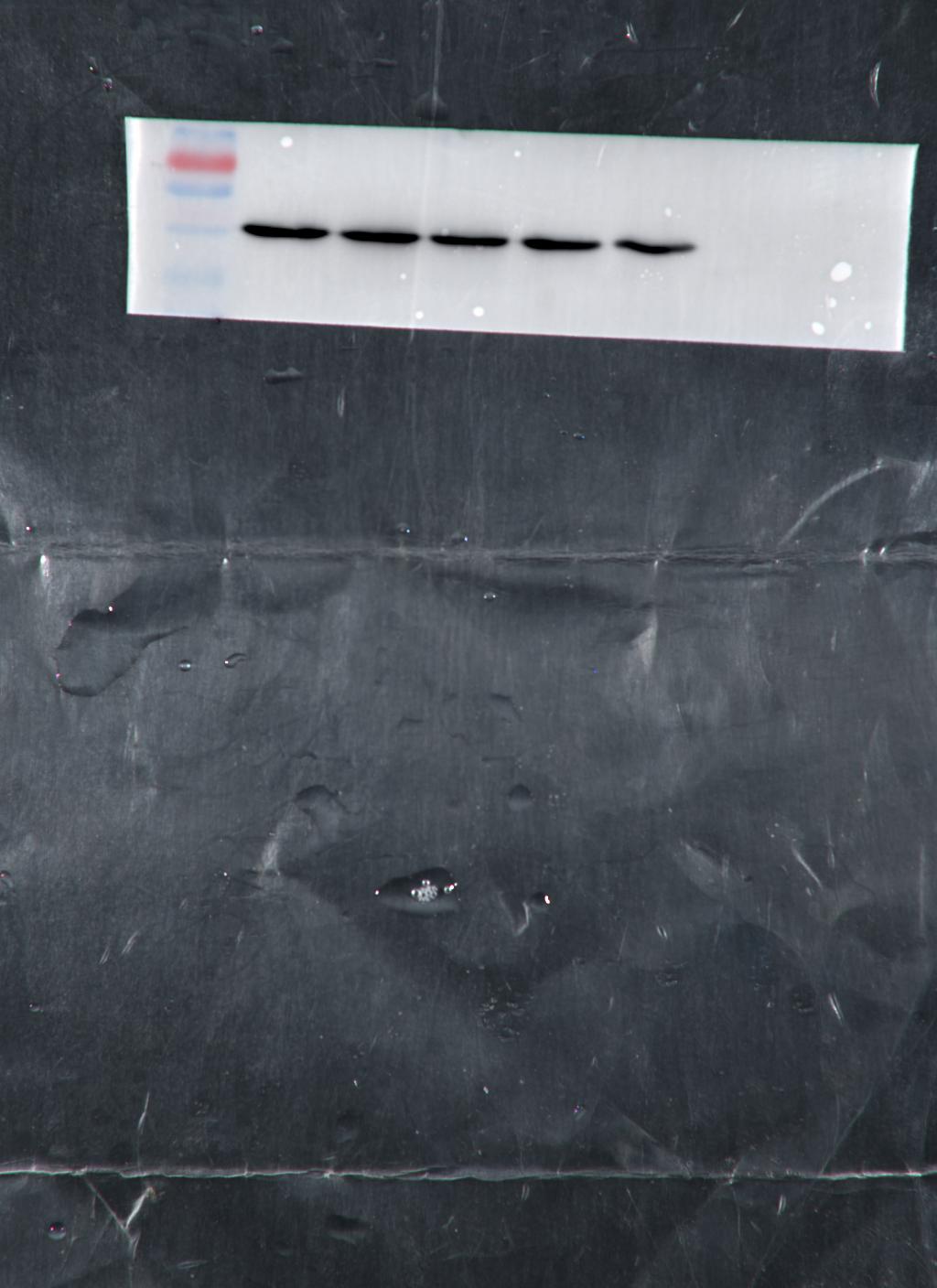

Supplement: Supplementary file 4 [file datasheet1.zip › (2020.8.14) WB image/original image files of the western blots/actin/zactin-0 2017.12.03_12.24.40_Ch+Marker.jpg]

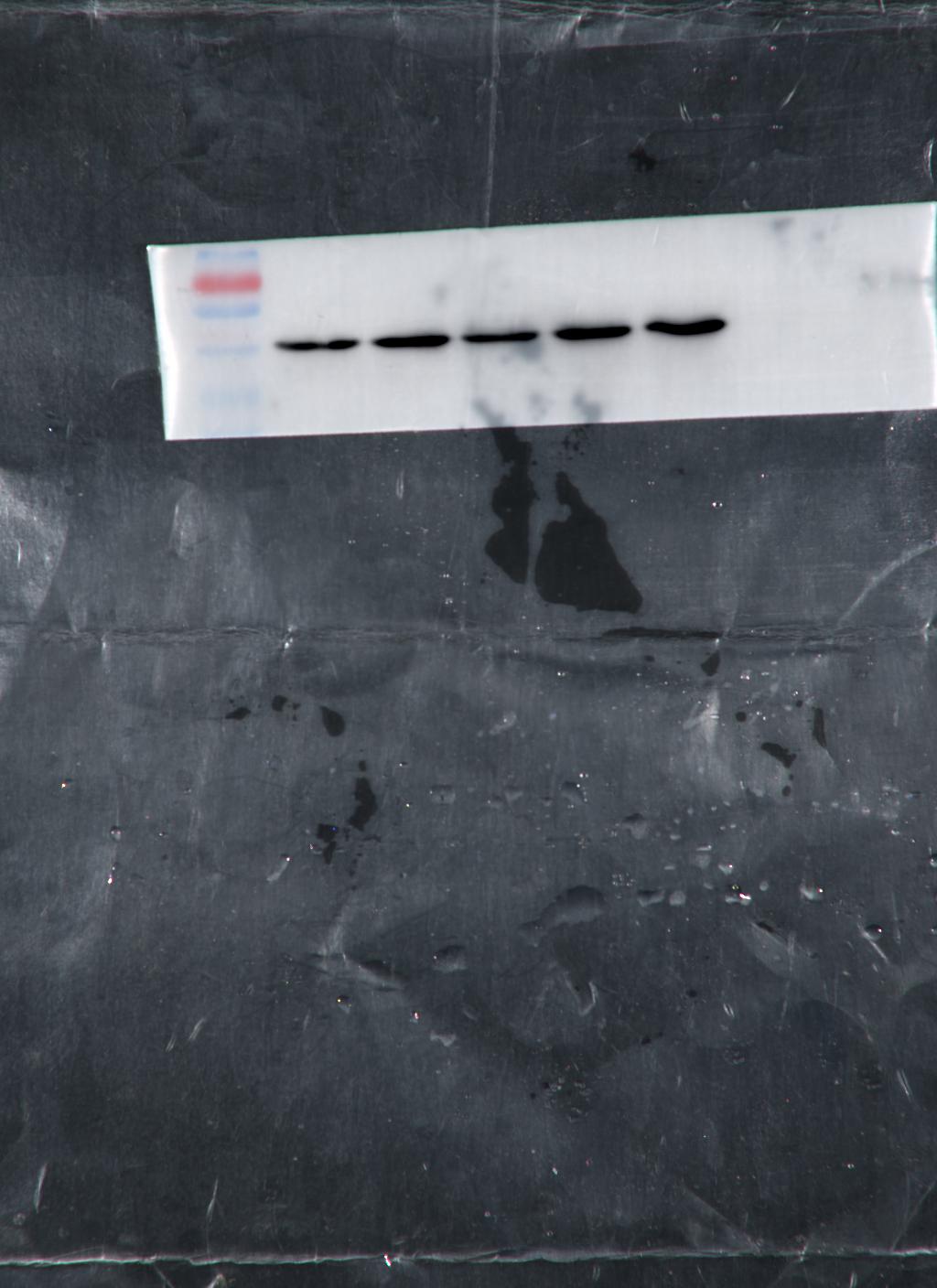

Supplement: Supplementary file 4 [file datasheet1.zip › (2020.8.14) WB image/original image files of the western blots/actin/zactin-2 2017.12.03_12.44.22_Ch+Marker.jpg]

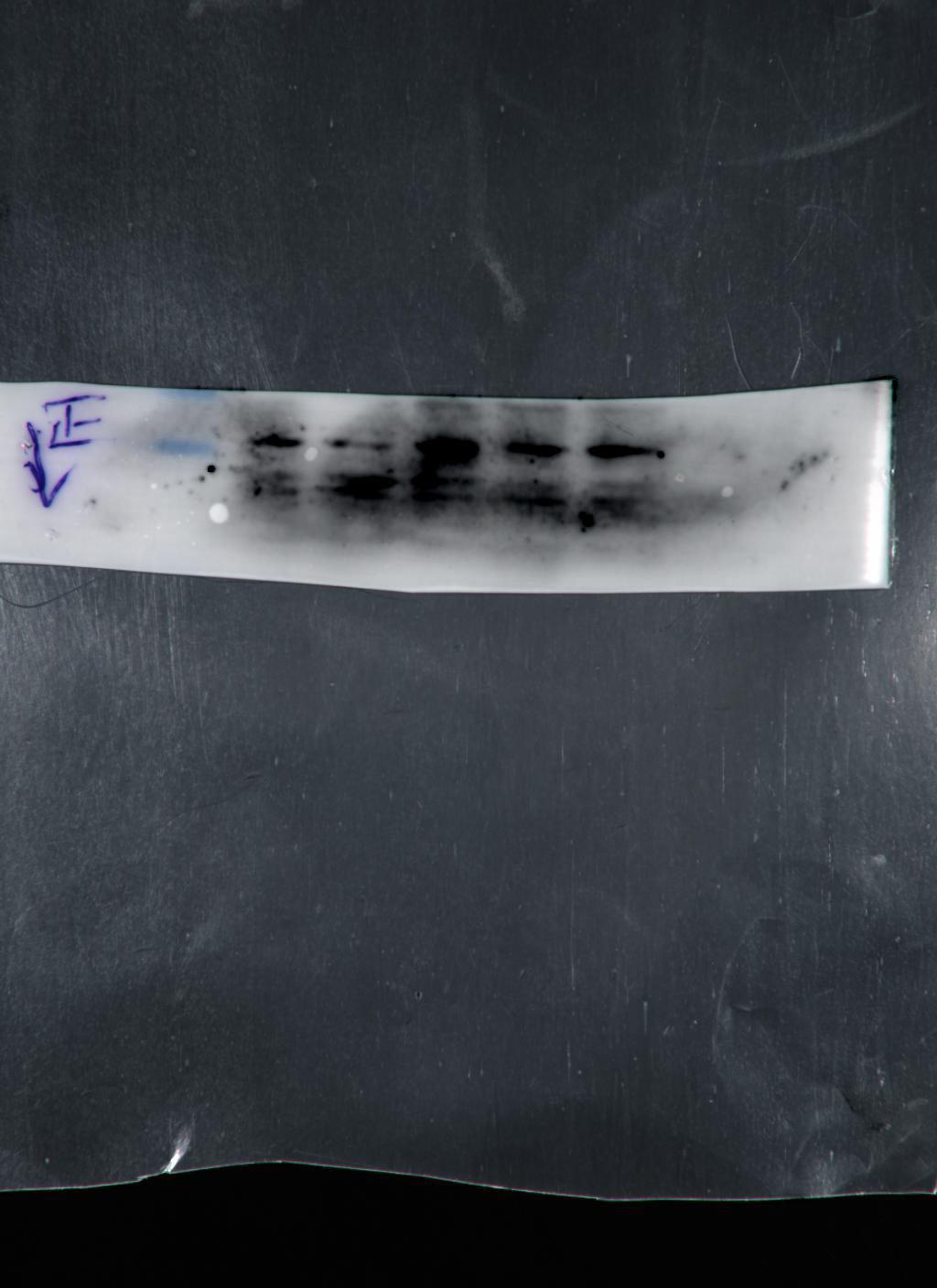

Supplement: Supplementary file 4 [file datasheet1.zip › (2020.8.14) WB image/original image files of the western blots/FAK/2017.05.18_01.16.35_Ch+Marker.jpg]

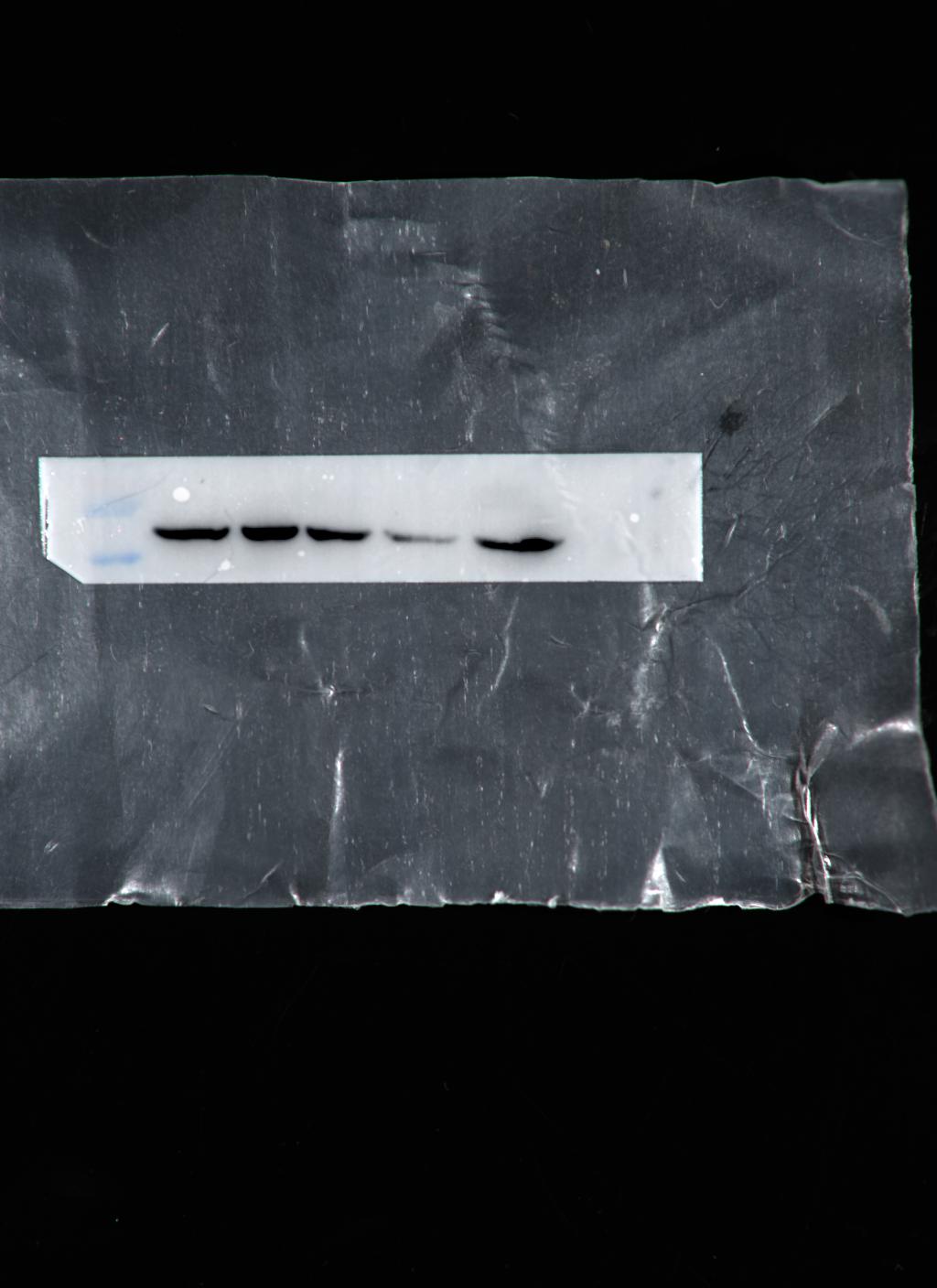

Supplement: Supplementary file 4 [file datasheet1.zip › (2020.8.14) WB image/original image files of the western blots/FAK/FAK (2).jpg]

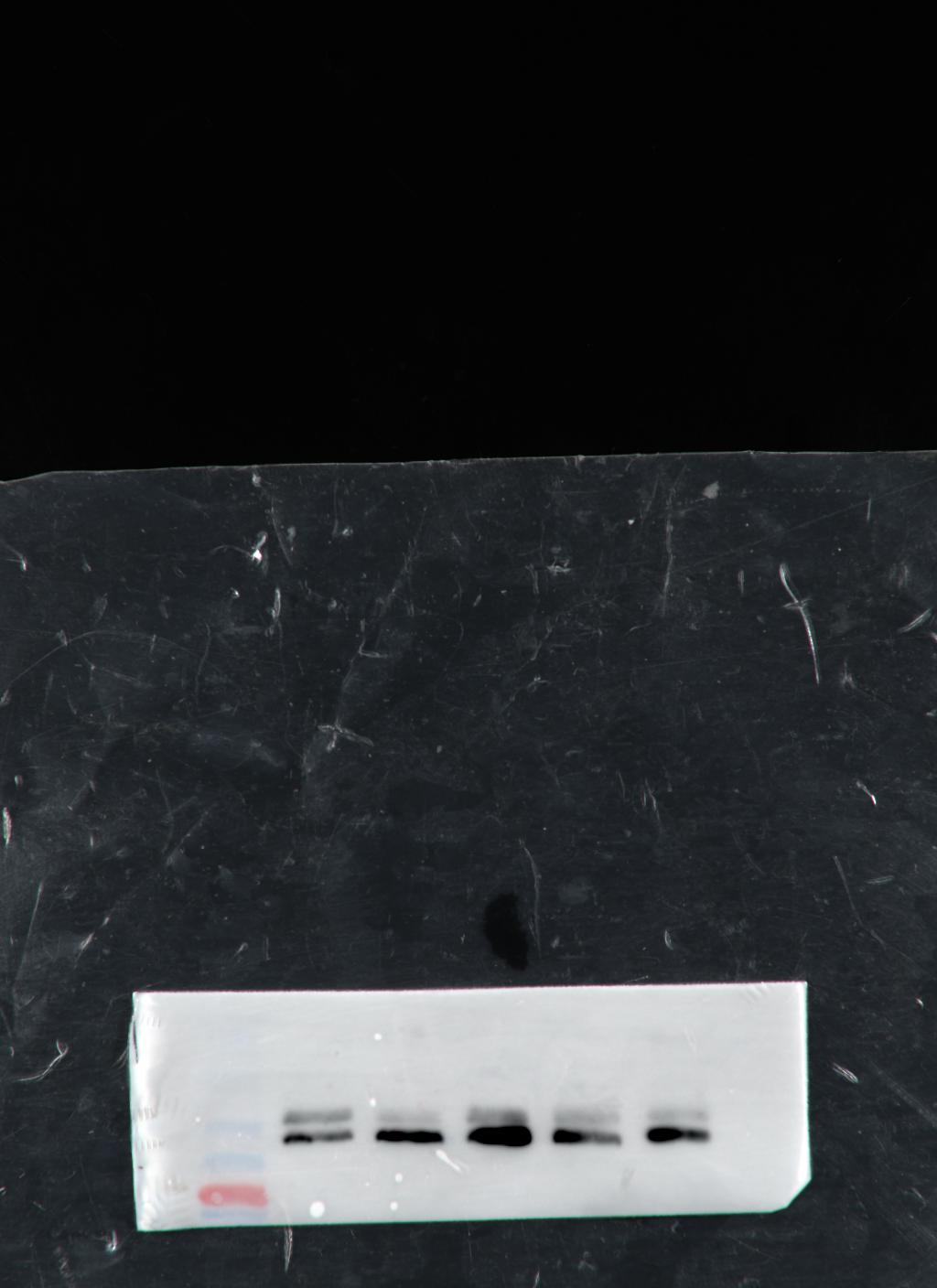

Supplement: Supplementary file 4 [file datasheet1.zip › (2020.8.14) WB image/original image files of the western blots/FAK/FAK.jpg]

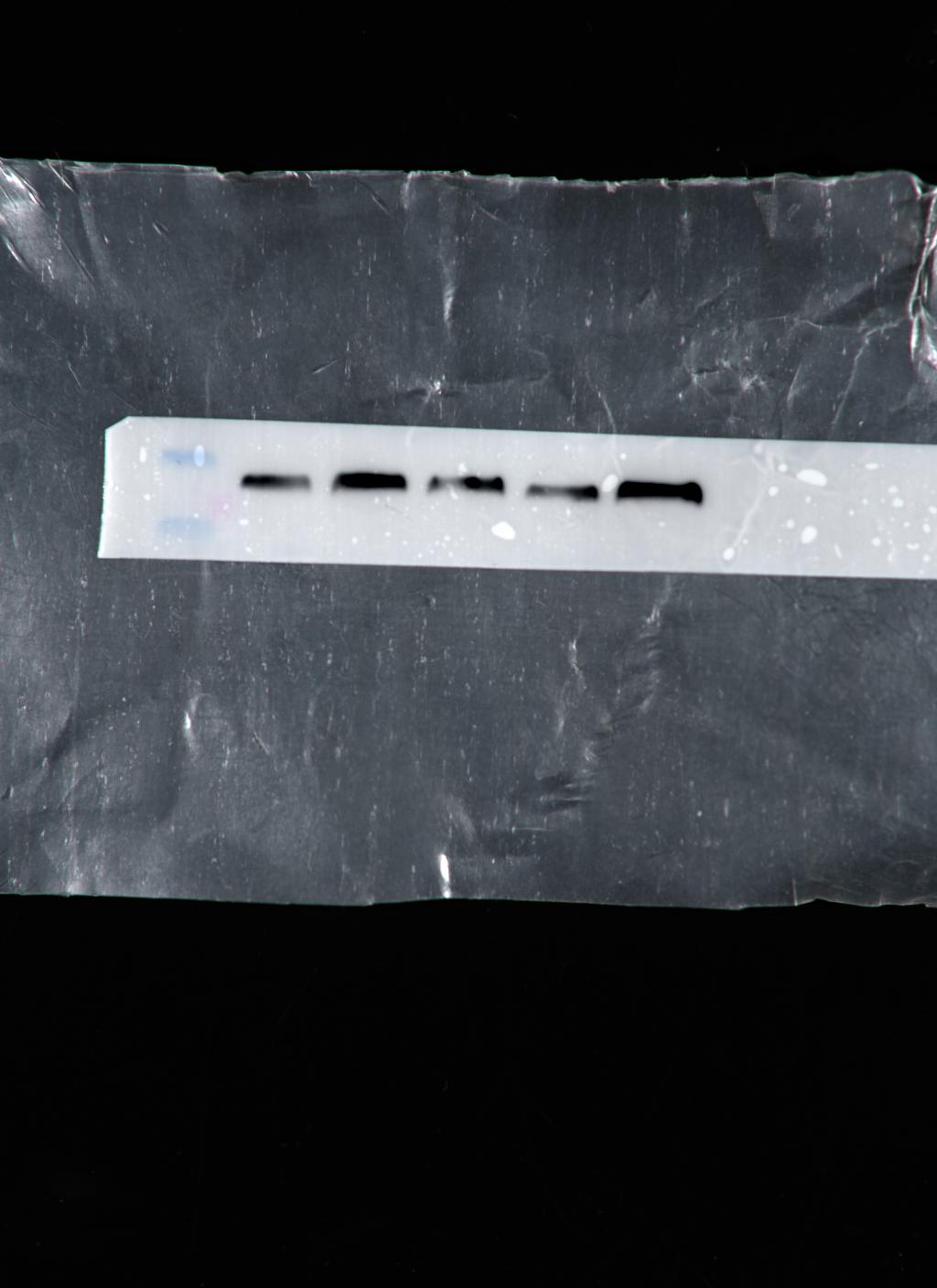

Supplement: Supplementary file 4 [file datasheet1.zip › (2020.8.14) WB image/original image files of the western blots/FAK/wsp fak 0811 2018.08.11_10.11.32_Ch+Marker.jpg]

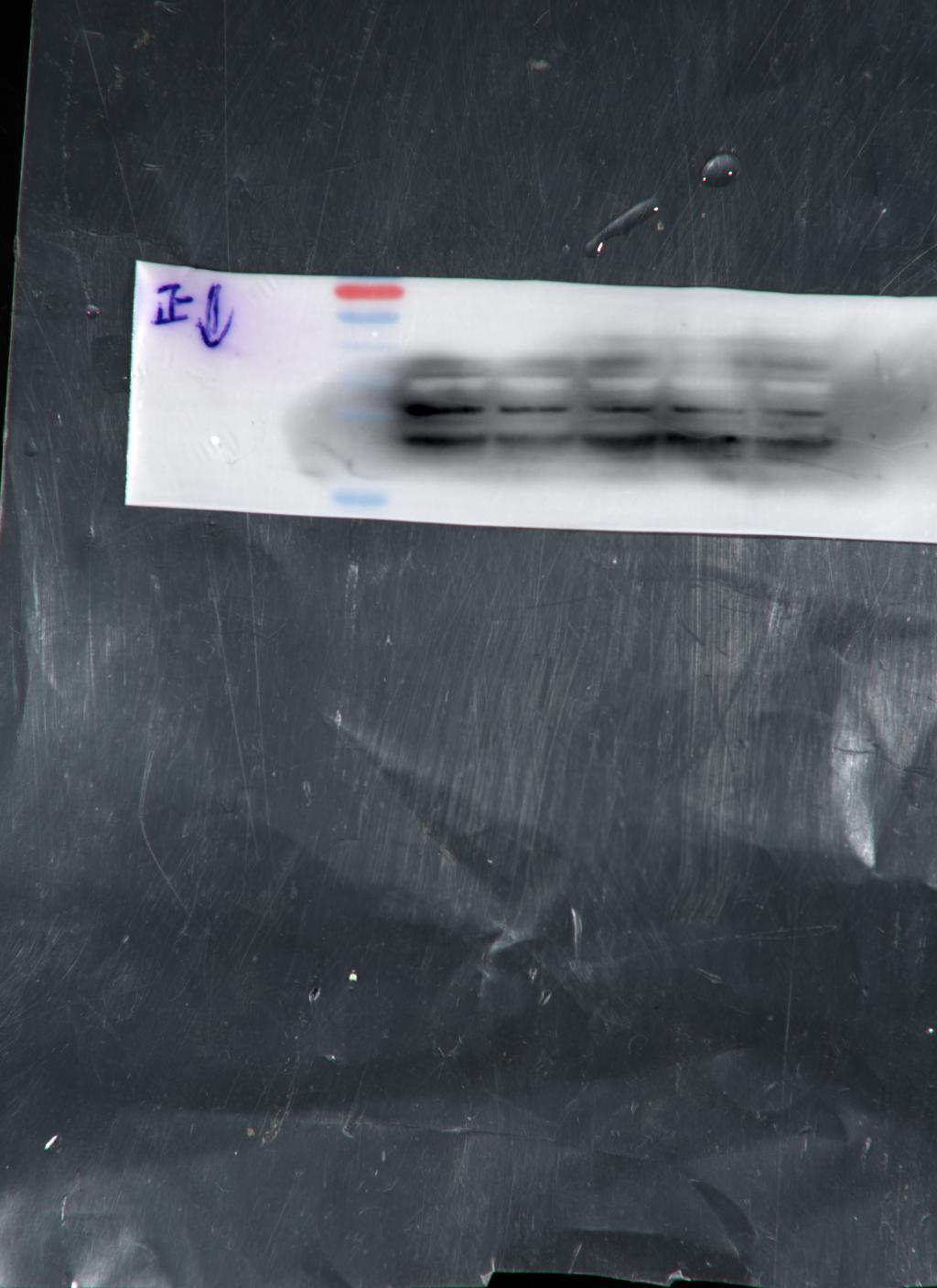

Supplement: Supplementary file 4 [file datasheet1.zip › (2020.8.14) WB image/original image files of the western blots/Paxillin/2017.05.06_08.15.21_Ch+Marker.jpg]

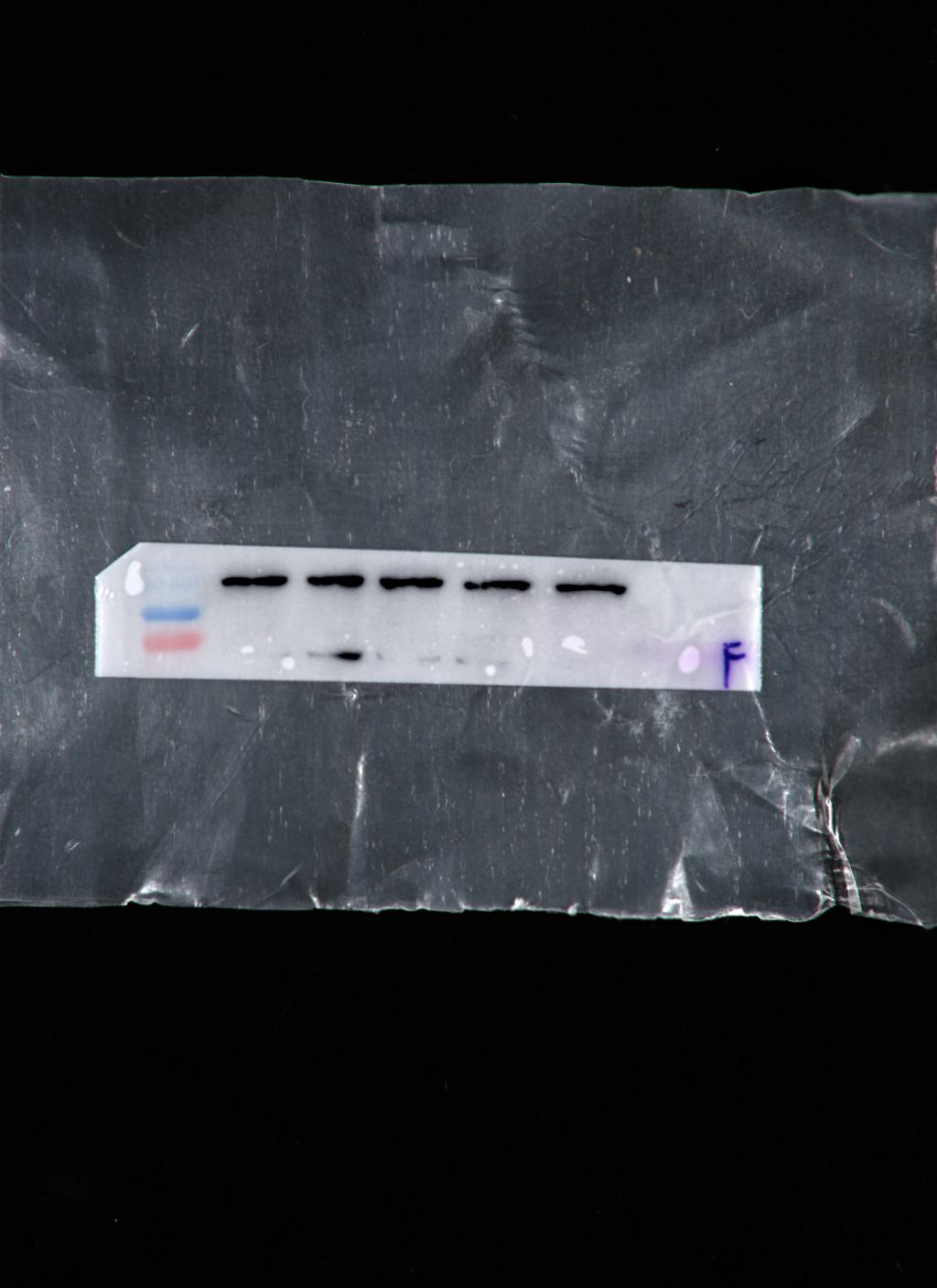

Supplement: Supplementary file 4 [file datasheet1.zip › (2020.8.14) WB image/original image files of the western blots/Paxillin/Paxillin.jpg]

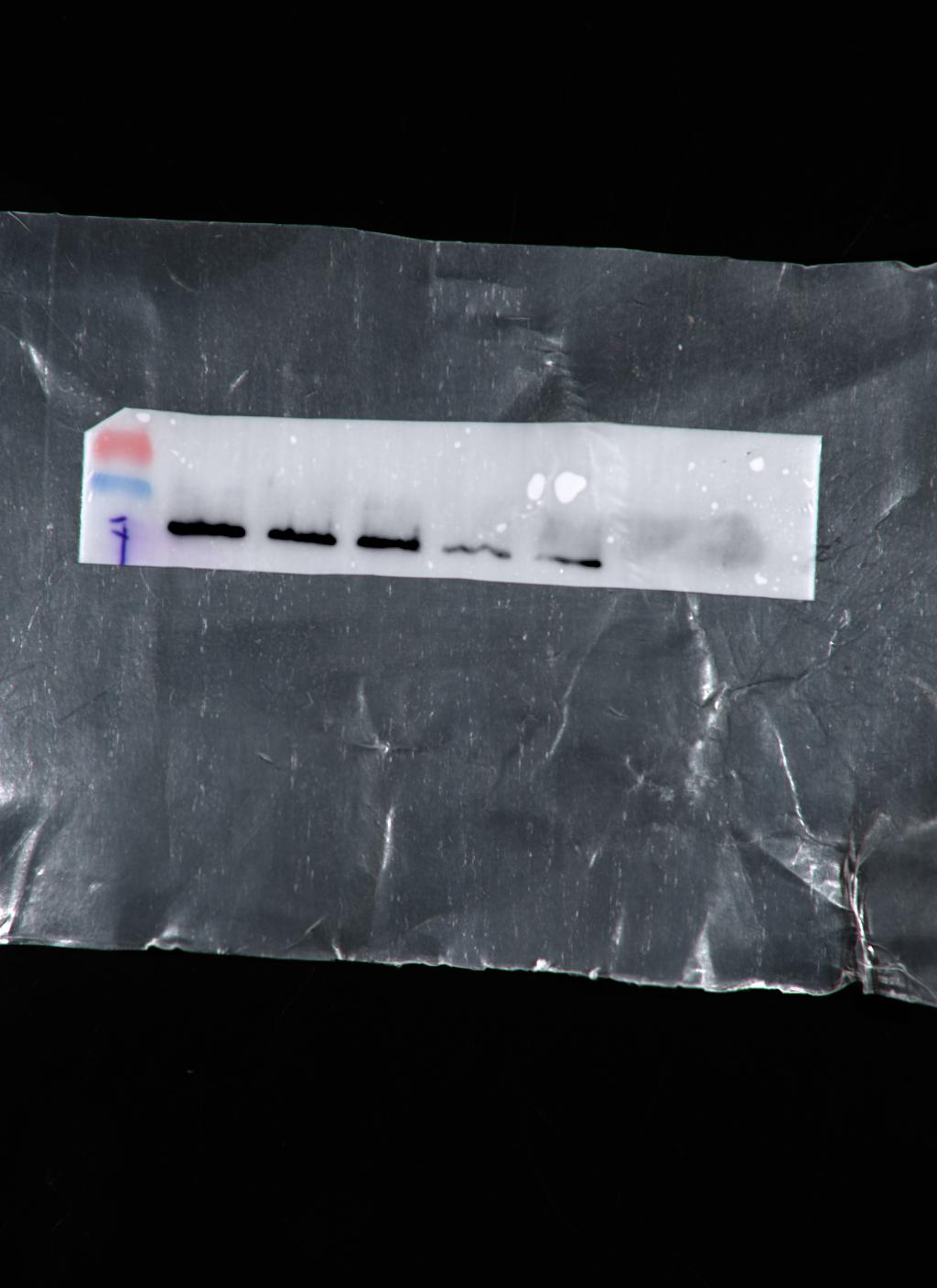

Supplement: Supplementary file 4 [file datasheet1.zip › (2020.8.14) WB image/original image files of the western blots/Paxillin/wsp Paxillin 080904 2018.08.09_13.36.03_Ch+Marker.jpg]

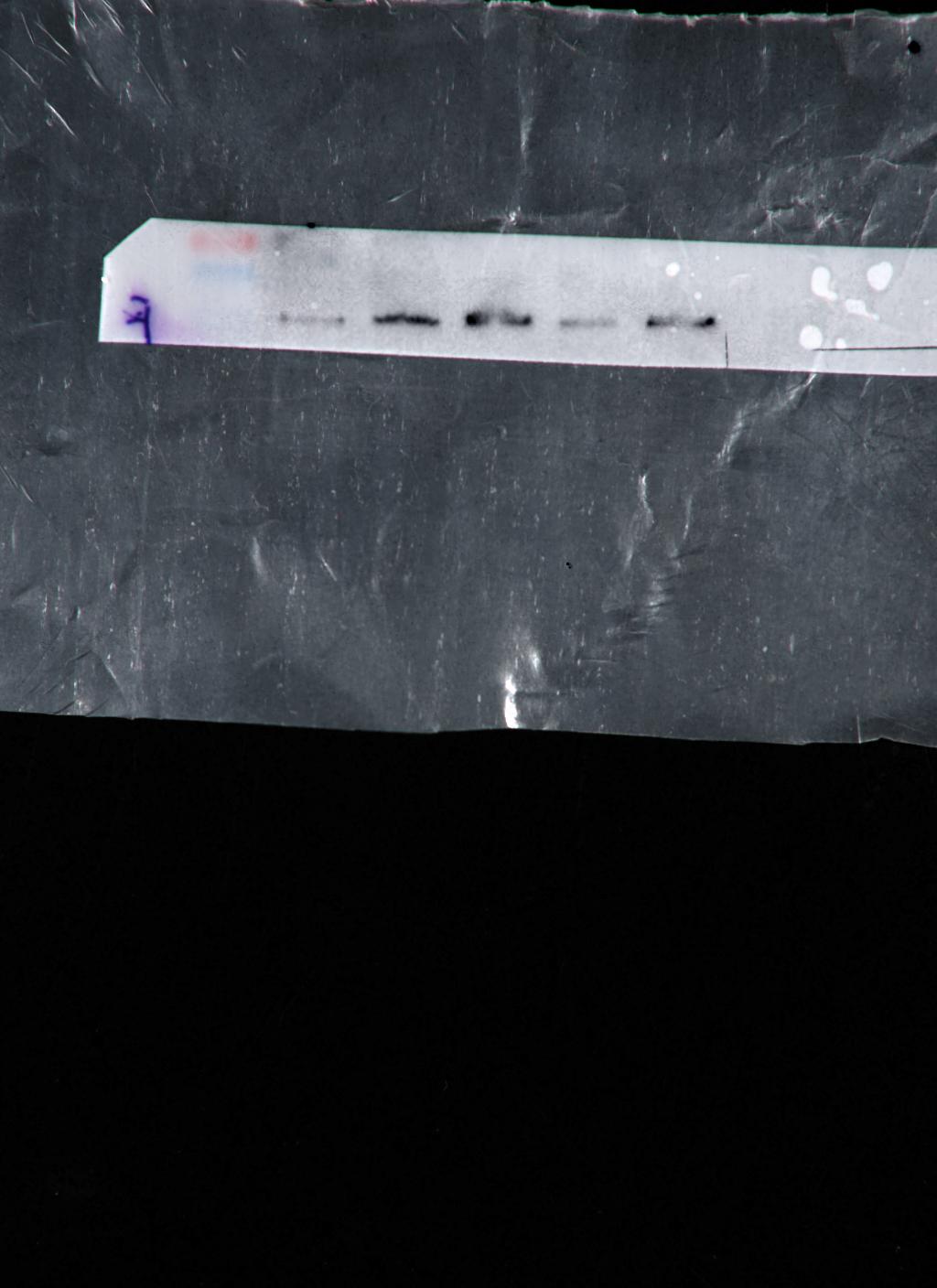

Supplement: Supplementary file 4 [file datasheet1.zip › (2020.8.14) WB image/original image files of the western blots/Paxillin/wsp Paxillin081102 2018.08.11_10.17.53_Ch+Marker.jpg]

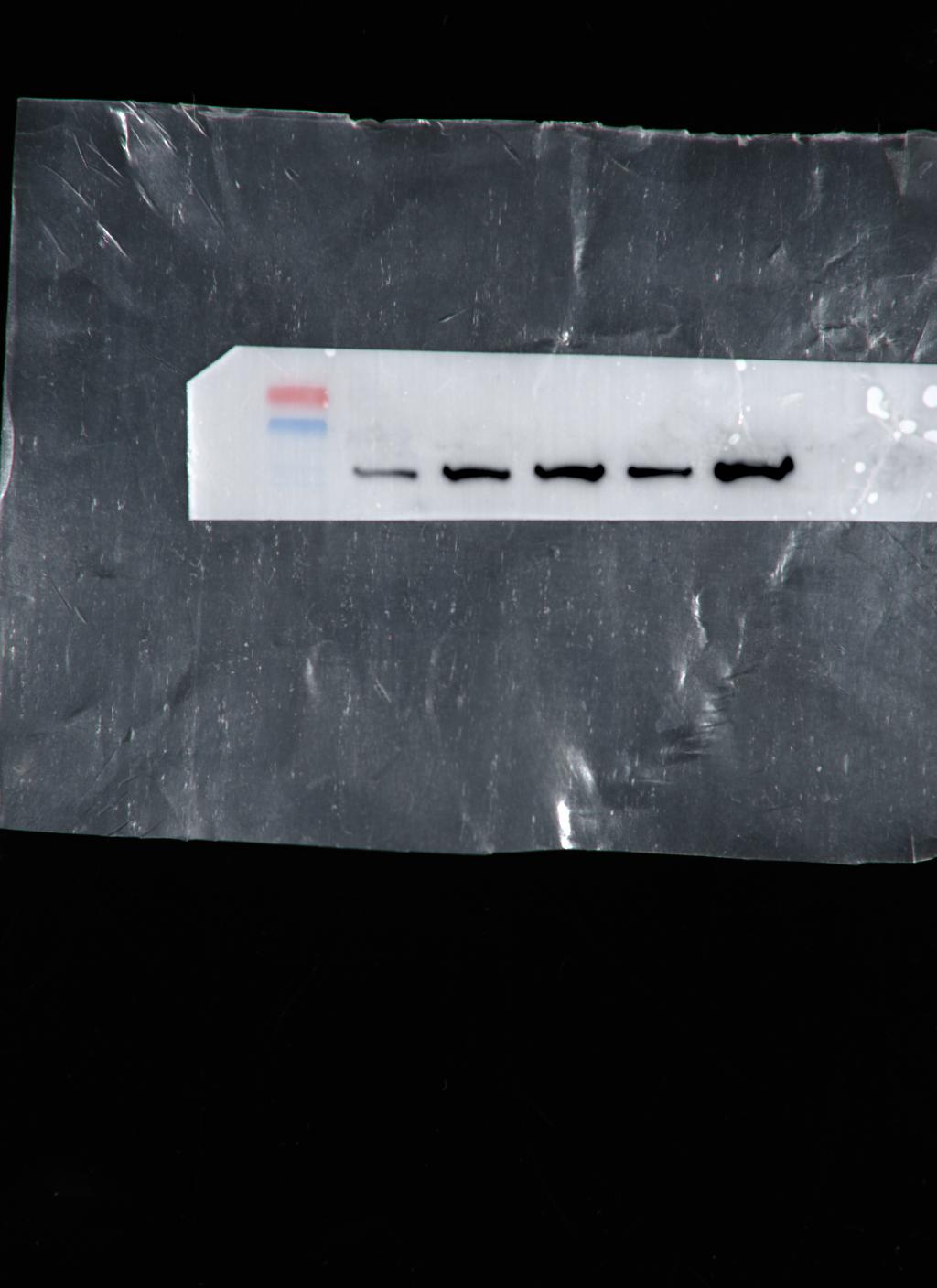

Supplement: Supplementary file 4 [file datasheet1.zip › (2020.8.14) WB image/original image files of the western blots/Paxillin/wsp Paxillin081103 2018.08.11_10.20.51_Ch+Marker.jpg]

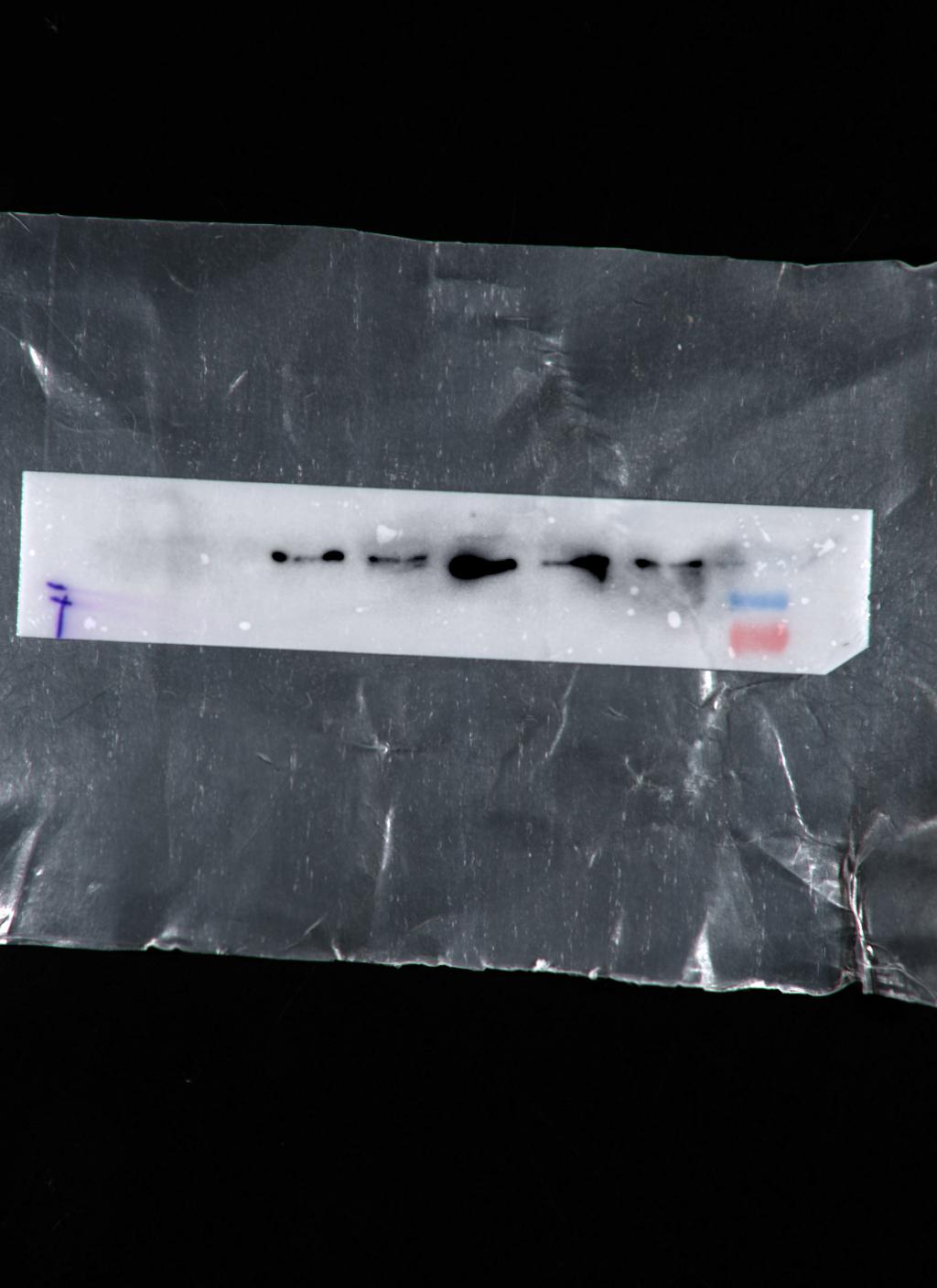

Supplement: Supplementary file 4 [file datasheet1.zip › (2020.8.14) WB image/original image files of the western blots/Paxillin/wspPaxillin 080902 2018.08.09_13.30.17_Ch+Marker.jpg]

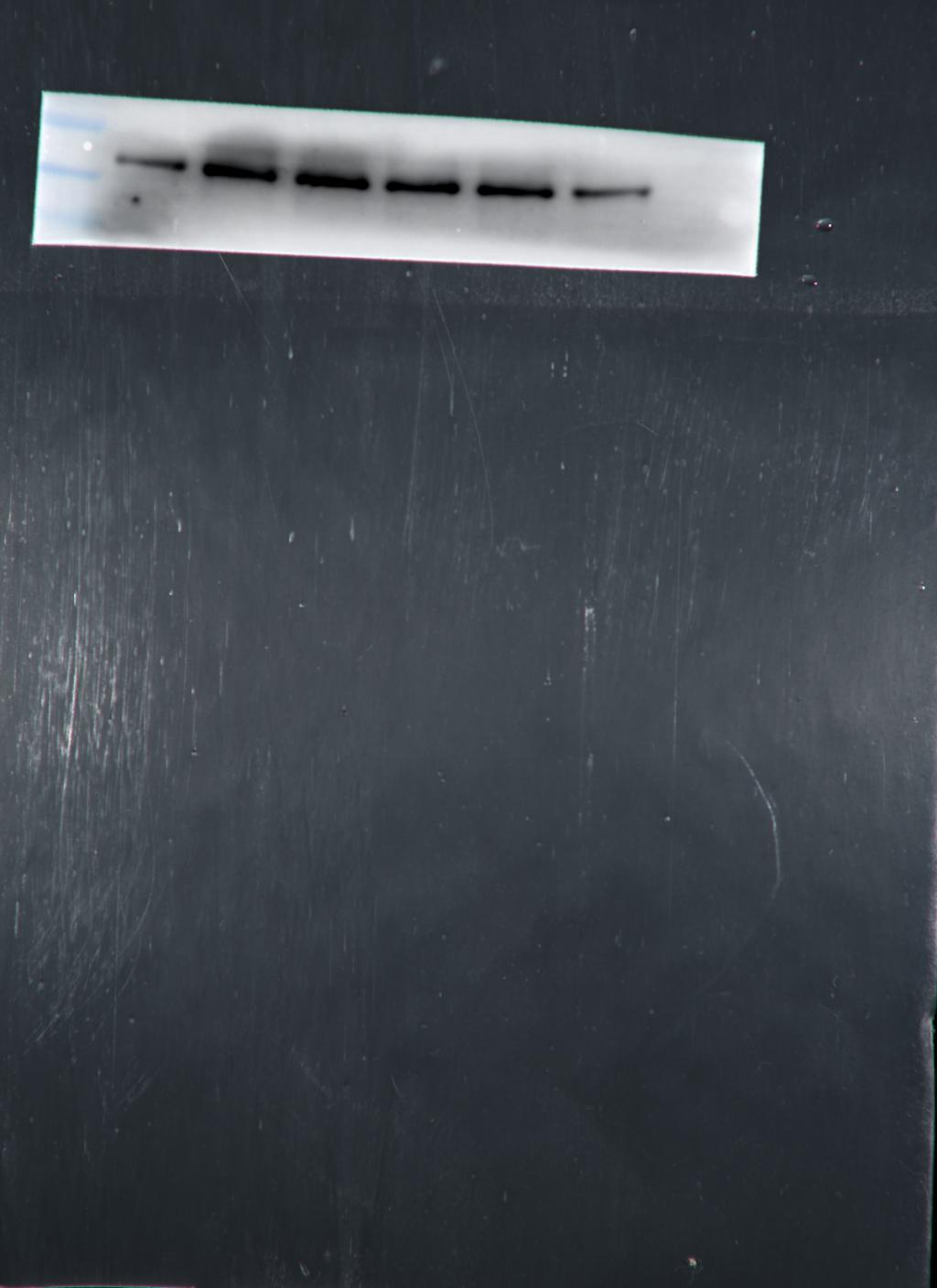

Supplement: Supplementary file 4 [file datasheet1.zip › (2020.8.14) WB image/original image files of the western blots/VEGF/2017.01.09_22.54.41_Ch+Marker.jpg]

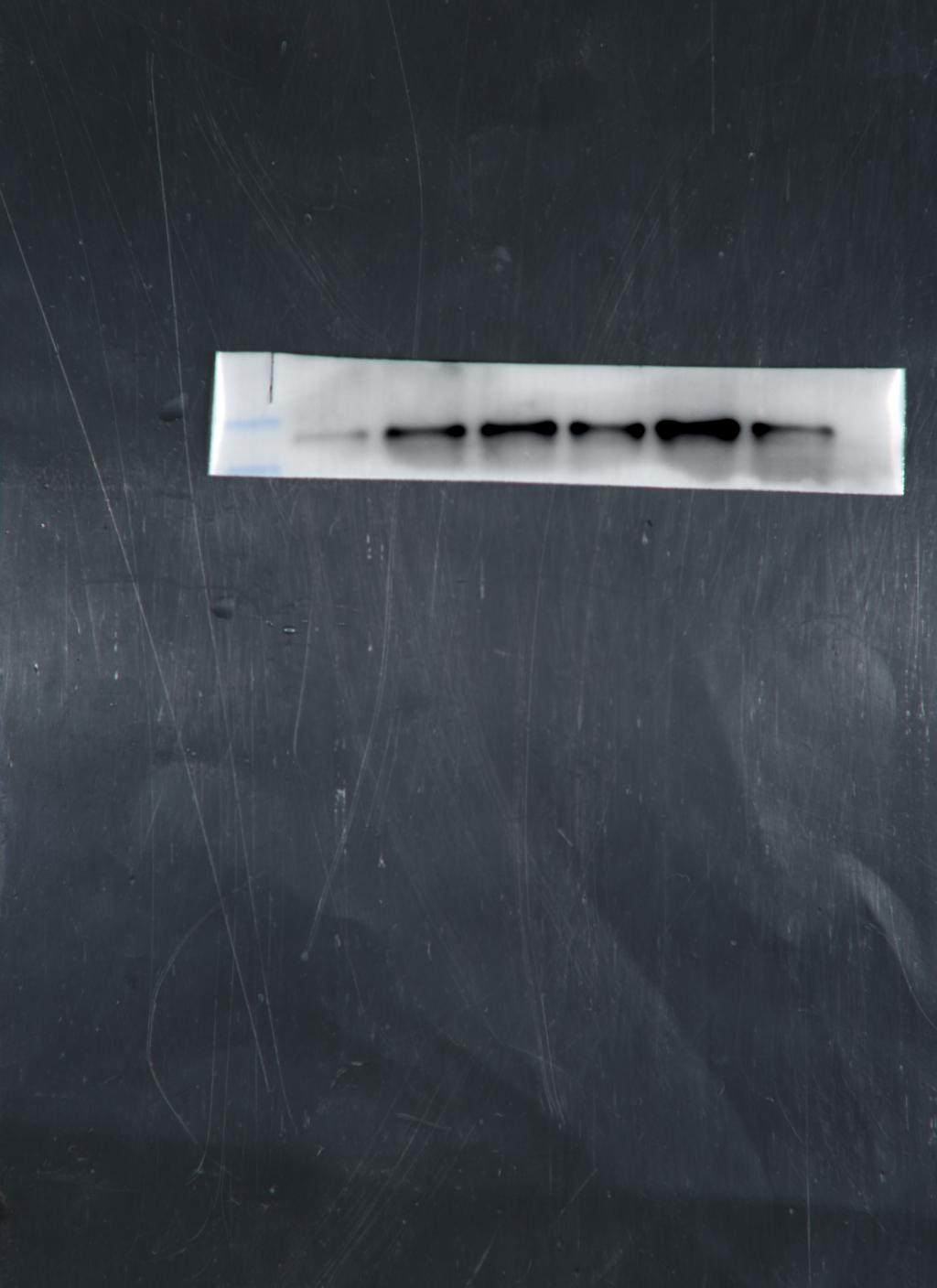

Supplement: Supplementary file 4 [file datasheet1.zip › (2020.8.14) WB image/original image files of the western blots/VEGF/2017.01.14_23.29.26_Ch+Marker.jpg]

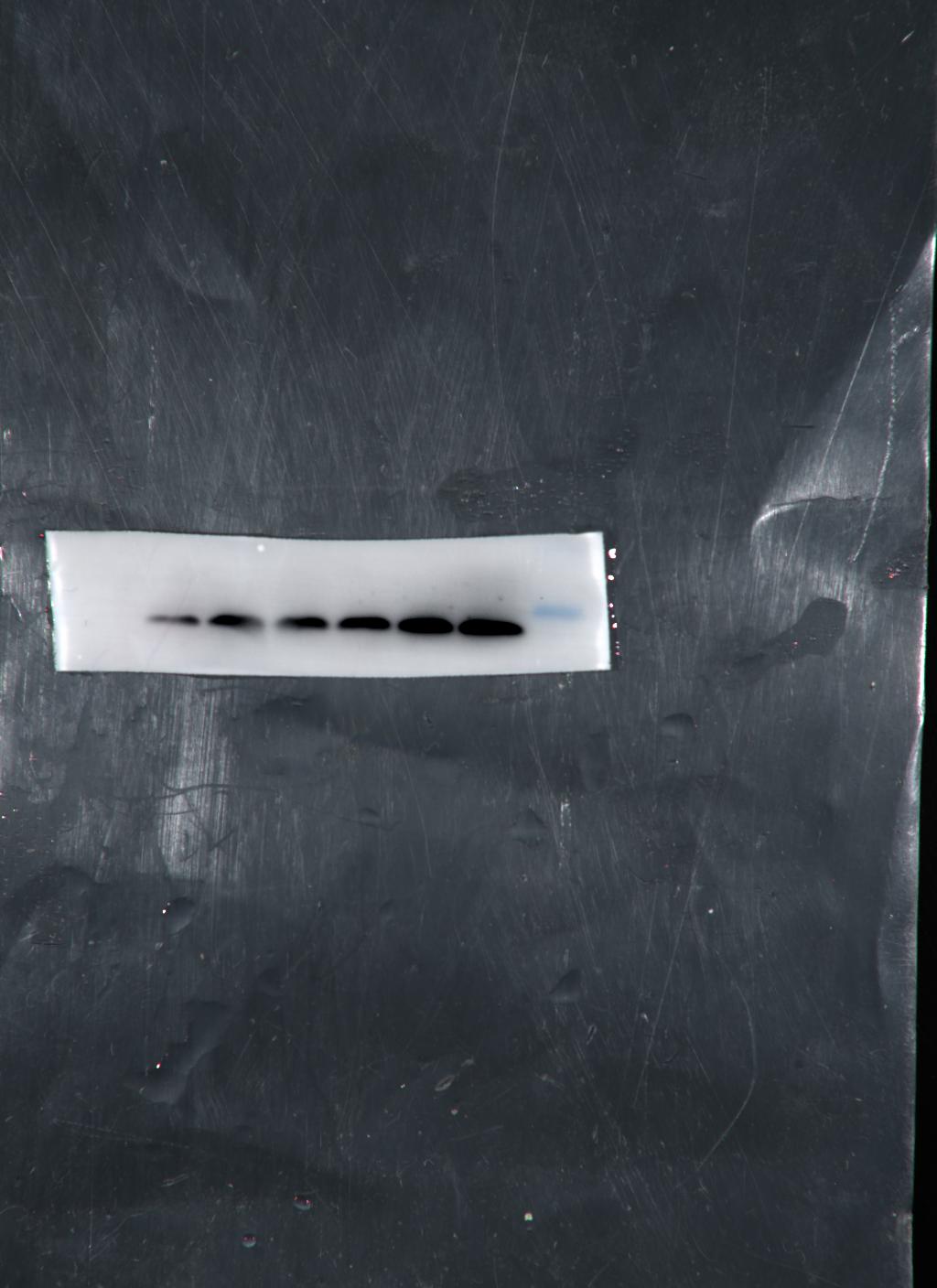

Supplement: Supplementary file 4 [file datasheet1.zip › (2020.8.14) WB image/original image files of the western blots/VEGF/2017.04.27_08.07.37_Ch+Marker.jpg]

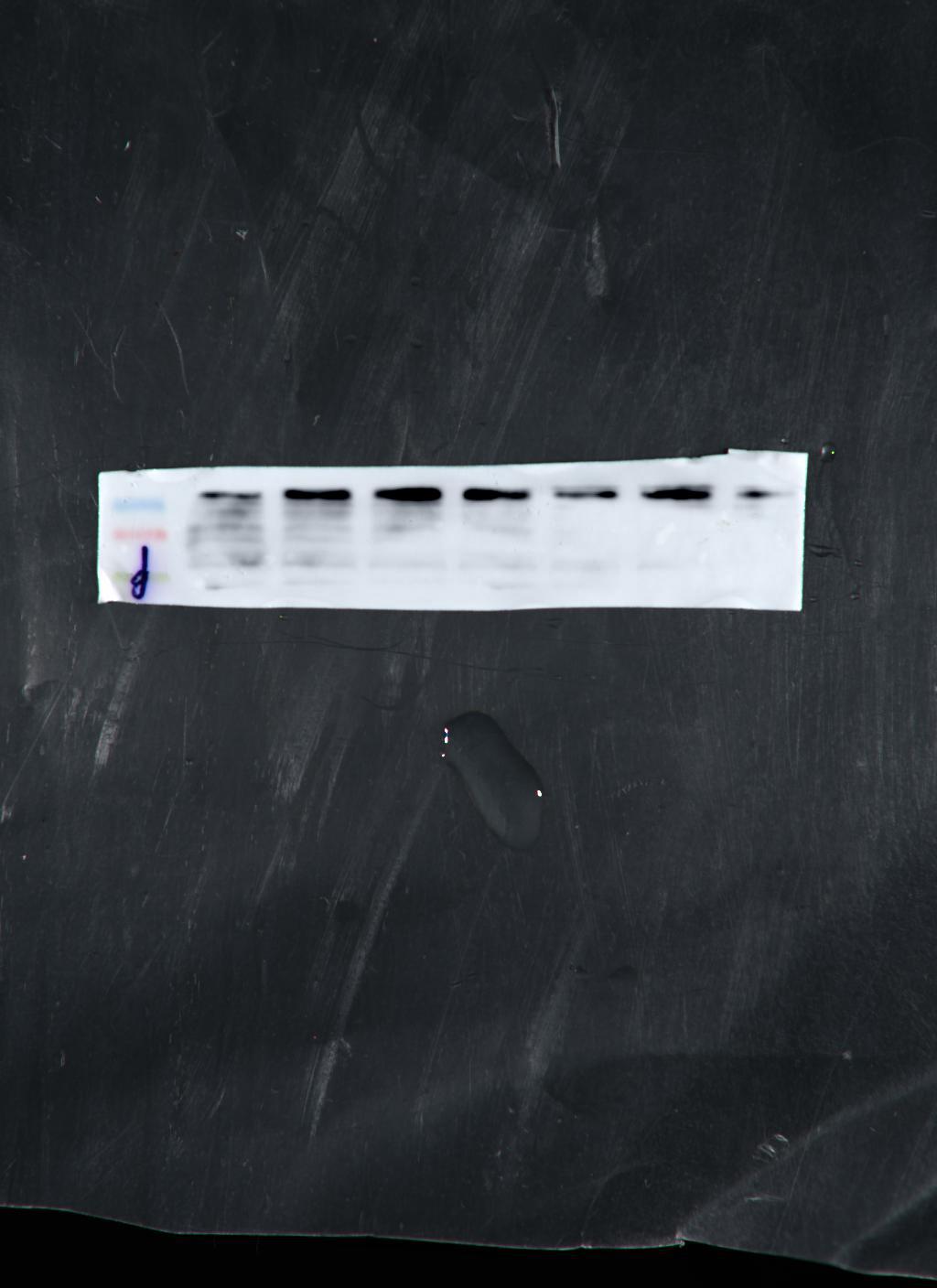

Supplement: Supplementary file 4 [file datasheet1.zip › (2020.8.14) WB image/original image files of the western blots/VEGF/20170520 actin dh 2017.05.19_22.43.52_Ch+Marker.jpg]

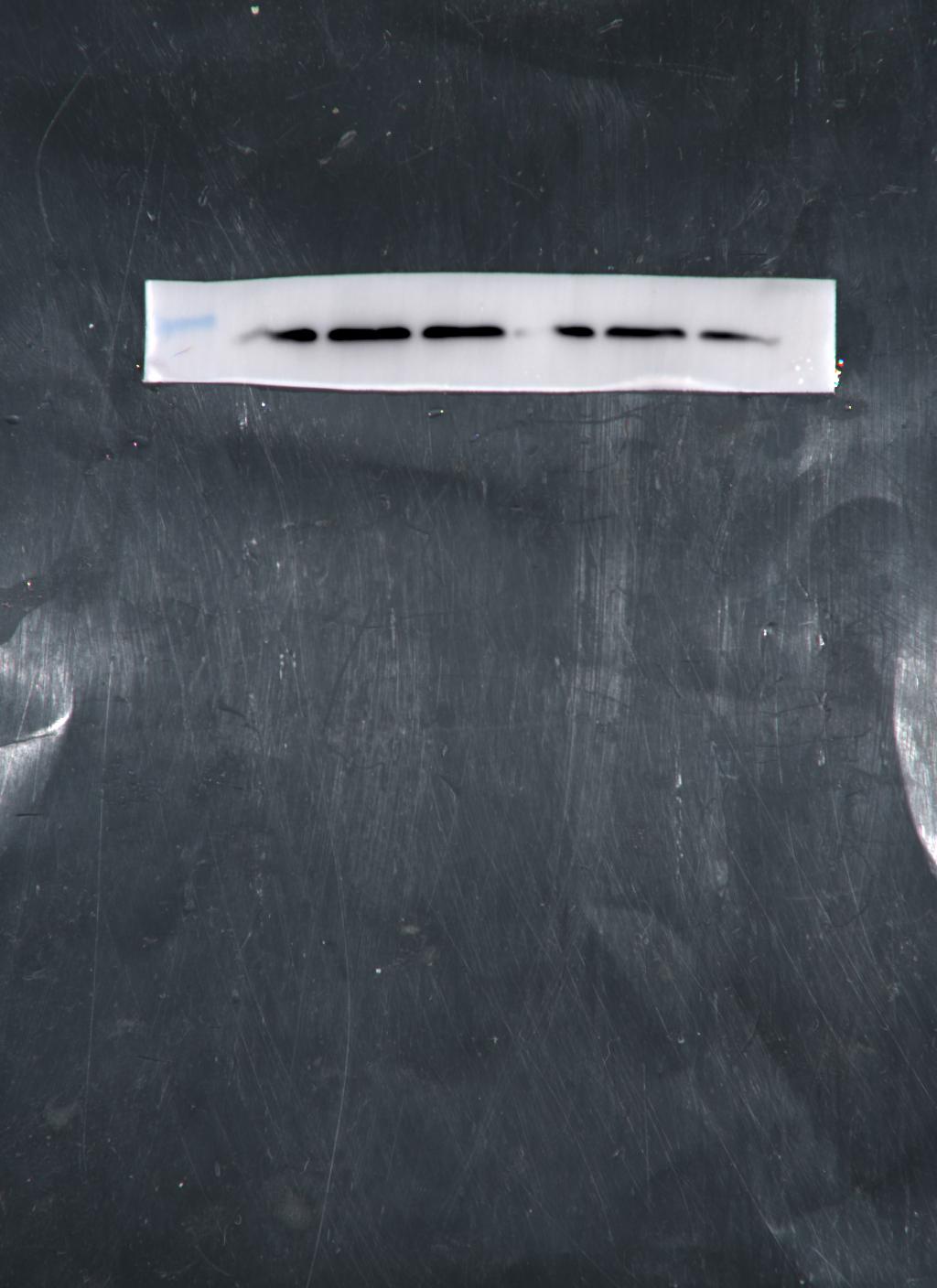

Supplement: Supplementary file 4 [file datasheet1.zip › (2020.8.14) WB image/original image files of the western blots/VEGF/VEGF+Marker.jpg]

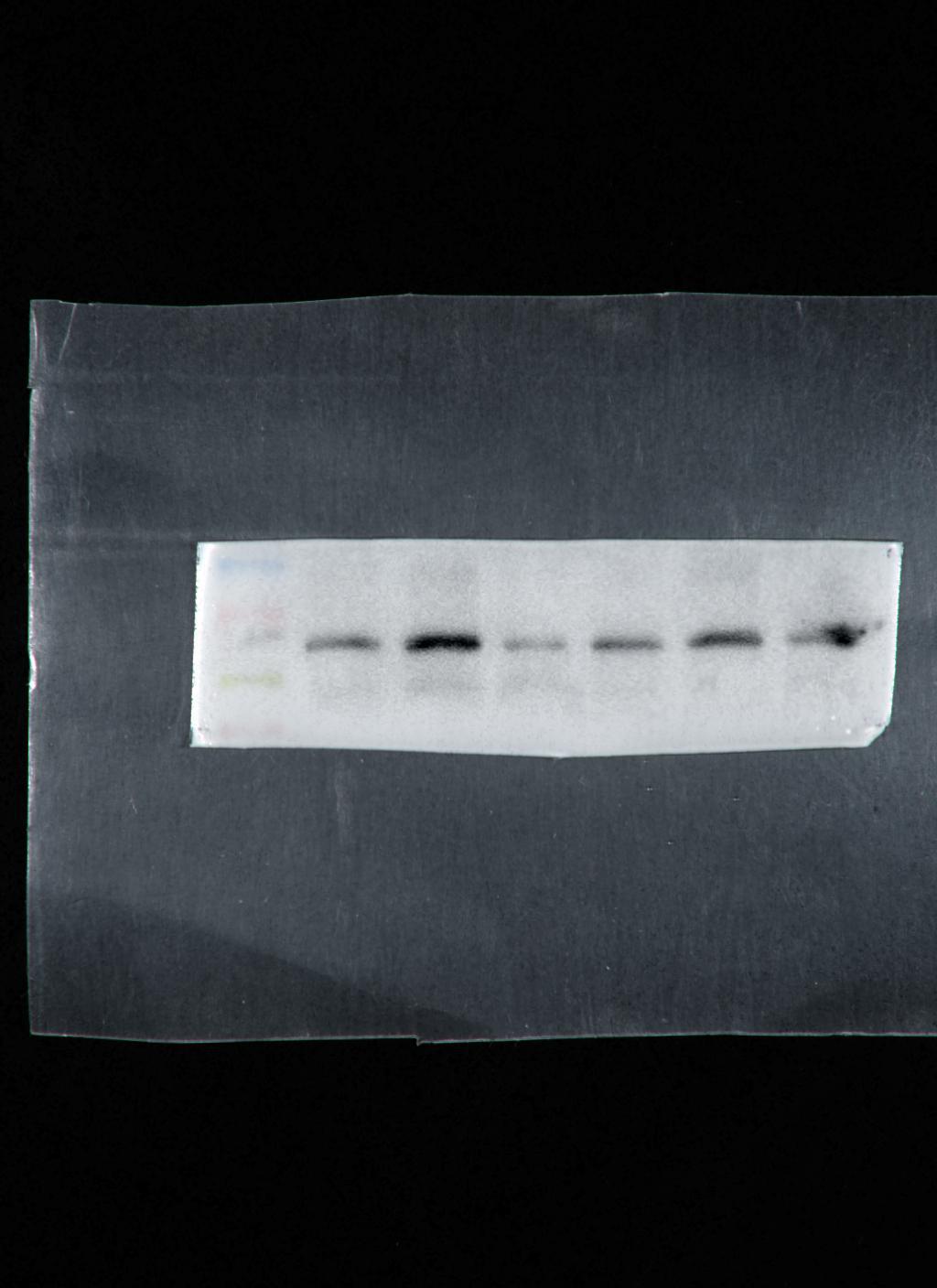

Supplement: Supplementary file 4 [file datasheet1.zip › (2020.8.14) WB image/original image files of the western blots/VEGF/VEGF.jpg]

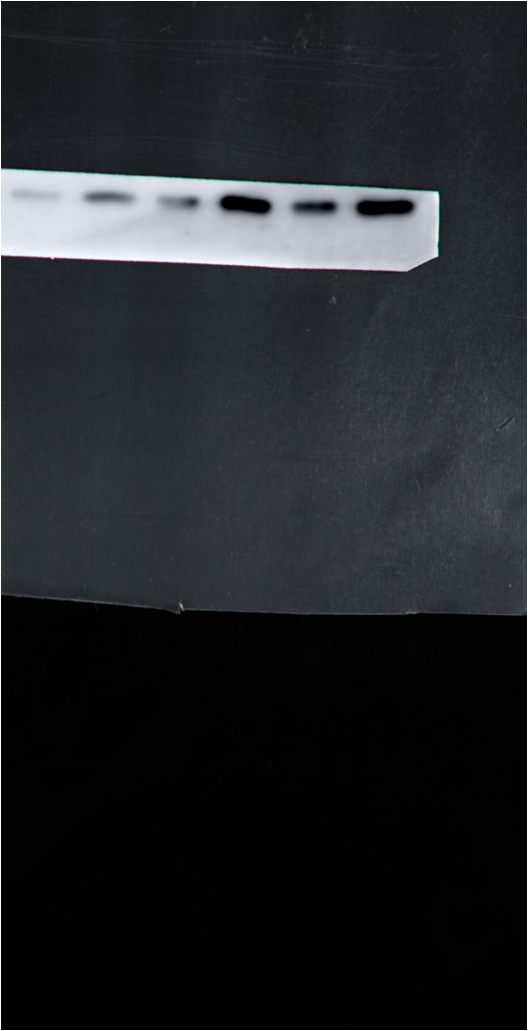

Supplement: Supplementary file 4 [file datasheet1.zip › (2020.8.14) WB image/original image files of the western blots/VEGF/VEGF.tif]

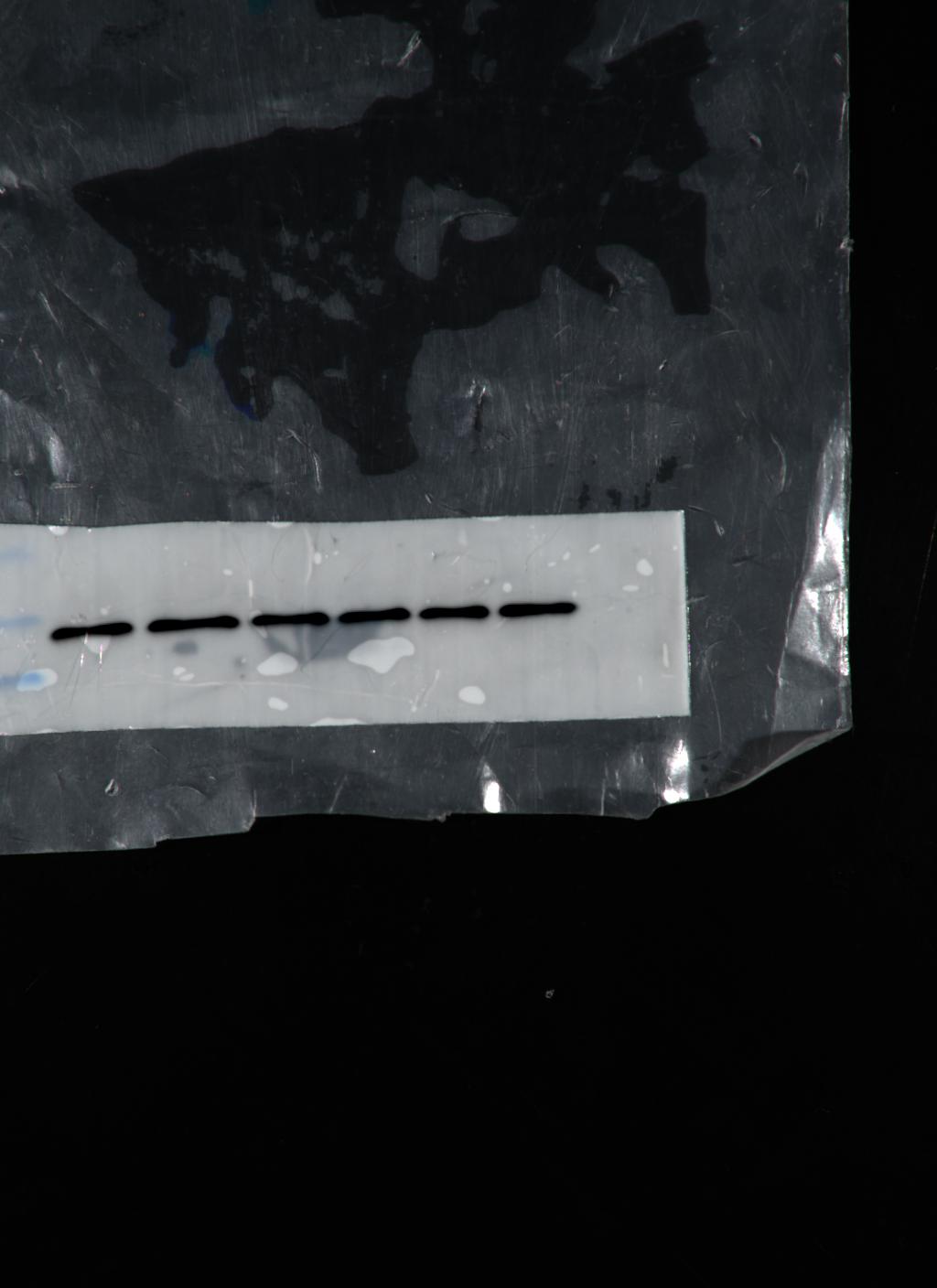

Supplement: Supplementary file 4 [file datasheet1.zip › (2020.8.14) WB image/original image files of the western blots/actin/2017.07.18_22.34.45_Ch+Marker.jpg]

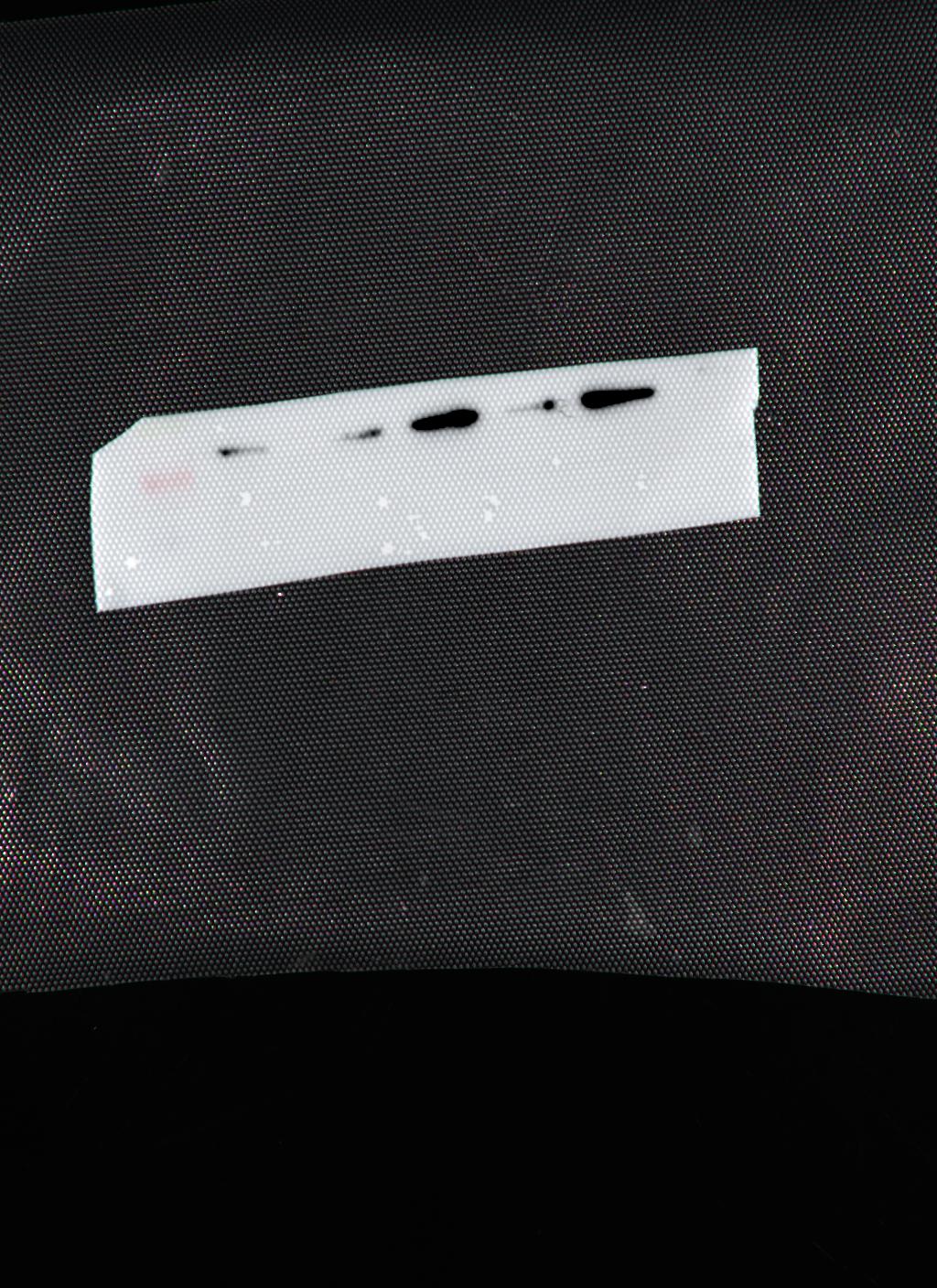

Supplement: Supplementary file 4 [file datasheet1.zip › (2020.8.14) WB image/original image files of the western blots/p-FAk/2017.05.17_23.09.26_Ch+Marker.jpg]

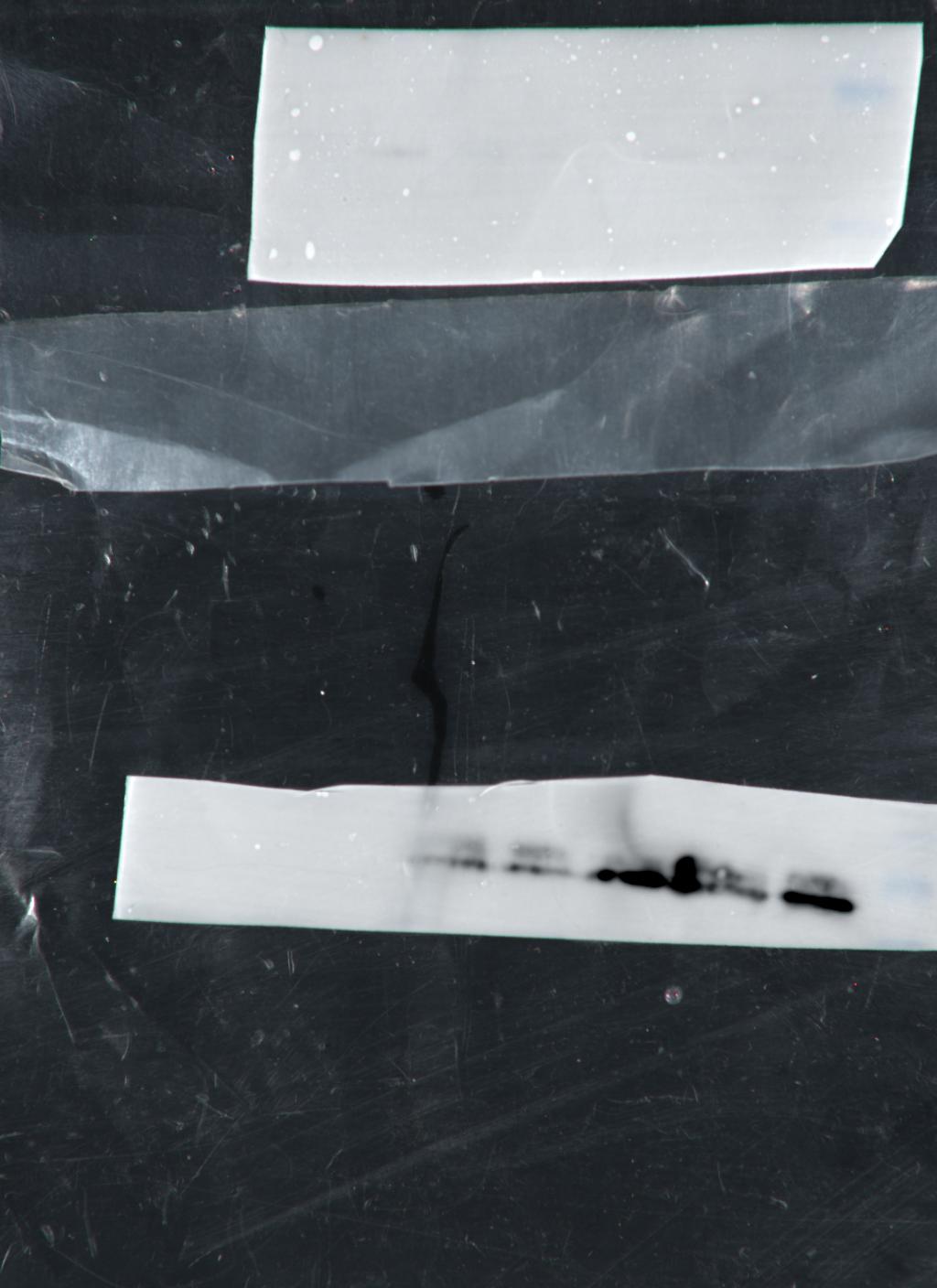

Supplement: Supplementary file 4 [file datasheet1.zip › (2020.8.14) WB image/original image files of the western blots/p-FAk/2017.05.17_23.34.18_Ch+Marker.jpg]

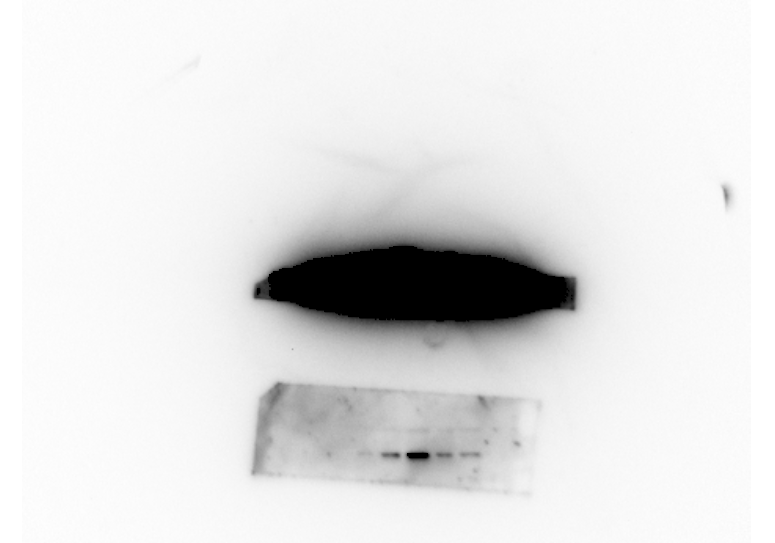

Supplement: Supplementary file 4 [file datasheet1.zip › (2020.8.14) WB image/original image files of the western blots/p-Paxillin/2020_8_8 paxillin.tif]

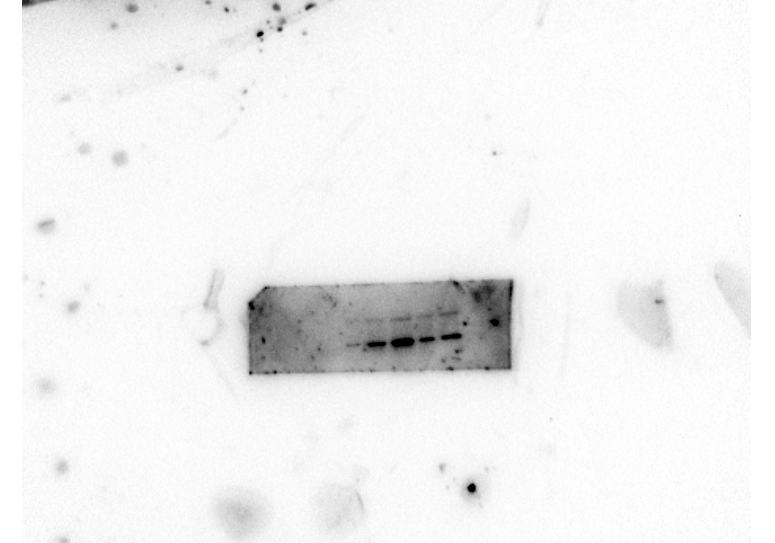

Supplement: Supplementary file 4 [file datasheet1.zip › (2020.8.14) WB image/original image files of the western blots/p-Paxillin/2020_8_8n paxillin 1.tif]

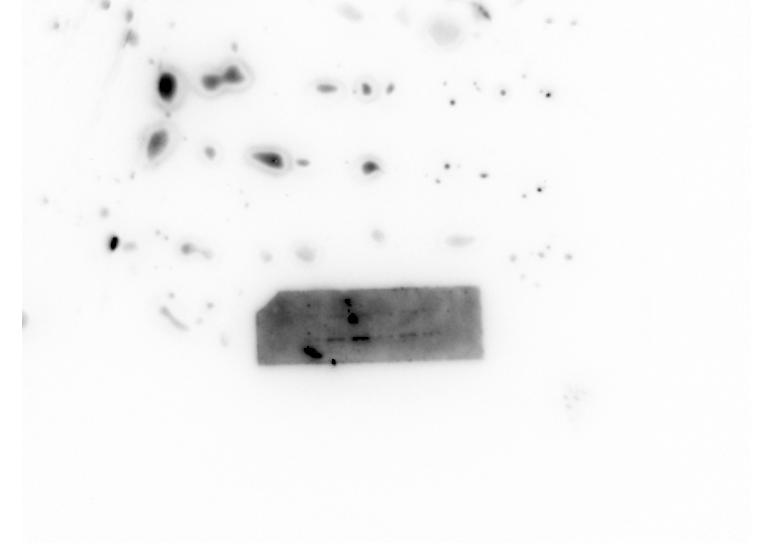

Supplement: Supplementary file 4 [file datasheet1.zip › (2020.8.14) WB image/original image files of the western blots/p-Paxillin/2020_8_11 paxillin.tif]
